# Synergistic inhibition effects of andrographolide and baicalin on coronavirus mechanisms by downregulation of ACE2 protein level

**DOI:** 10.1038/s41598-024-54722-5

**Published:** 2024-02-21

**Authors:** Lina Wan, Yuchen Li, Wenhao Liao, Lizhen Lei, Maoyuan Zhao, Jinhao Zeng, Ziyi Zhao, Jianyuan Tang

**Affiliations:** 1 Hospital of Chengdu University of Traditional Chinese Medicine, Chengdu, 610072 China; 2 Laboratory Medicine, Hospital of Chengdu University of Traditional Chinese Medicine, Chengdu, 610072 China; 3 TCM Regulating Metabolic Diseases Key Laboratory of Si Chuan Province, Hospital of Chengdu University of Traditional Chinese Medicine, Chengdu, 610072 China; 4 Department of Digestive, Hospital of Chengdu University of Traditional Chinese Medicine, Chengdu, 610072 China

**Keywords:** Diseases, Medical research, Pathogenesis

## Abstract

The SARS-CoV-2 virus, belonging to the *Coronavirus* genus, which poses a threat to human health worldwide. Current therapies focus on inhibiting viral replication or using anti-inflammatory/immunomodulatory compounds to enhance host immunity. This makes the active ingredients of traditional Chinese medicine compounds ideal therapies due to their proven safety and minimal toxicity. Previous research suggests that andrographolide and baicalin inhibit coronaviruses; however, their synergistic effects remain unclear. Here, we studied the antiviral mechanisms of their synergistic use in vitro and in vivo. We selected the SARS-CoV-2 pseudovirus for viral studies and found that synergistic andrographolide and baicalein significantly reduced angiotensin-converting enzyme 2 protein level and viral entry of SARS-CoV-2 into cells compared to singal compound individually and inhibited the major protease activity of SARS-CoV-2. This mechanism is essential to reduce the pathogenesis of SARS-CoV-2. In addition, their synergistic use in vivo also inhibited the elevation of pro-inflammatory cytokines, including IL-6 and TNF-α—the primary cytokines in the development of acute respiratory distress syndrome (the main cause of COVID-19 deaths). In conclusion, this study shows that synergistic andrographolide and baicalein treatment acts as potent inhibitors of coronavirus mechanisms in vitro and in vivo—and is more effective together than in isolation.

## Introduction

Since 2002, a zoonotic coronavirus that causes respiratory disease, including the severe acute respiratory syndromes coronavirus (SARS-CoV), Middle East respiratory syndrome coronavirus, and the recent 2019 SARS-CoV-2 has caused three outbreaks^1^. The coronavirus disease 2019 (COVID-19) caused by SARS-CoV-2 is the most significant global public health event with high pathogenicity and infectivity^2^. So far, hundreds of millions of test-positive cases and tens of thousands of deaths have been confirmed worldwide (https://covid19.who.int/)^3^. The *Coronavirus* genus includes SARS-CoV-2, which is an enveloped single-stranded positive-sense RNA virus with high pathogenicity. Its spike (S) protein mediates viral entry into host cells^2,4,5^, infecting human bronchial epithelial cells, upper respiratory tract cells, and lung cells, leading to irreversible lung damage, life-threatening respiratory diseases, and multi-organ failure^6^. Currently, there are no specific prevention or treatment methods available^4^.

The genome RNA of SARS-CoV encodes a non-structural replicase polyprotein and structural proteins, including the S protein, nucleocapsid (N) protein, ion channel (E), and integral membrane (M) protein. The S protein is the most immunogenic of these proteins, and therefore related to vaccine development, diagnosis, and treatment^3,7^. The S protein consists of two subunits: the S1 subunit that binds to the host entry receptor angiotensin-converting enzyme 2 (ACE2) and the S2 subunit that mediates membrane fusion^7^. The ACE2 protein is the receptor for coronavirus entry into human cells^3,4,8^, and is widely distributed in human lung and airway epithelial cells, intestinal epithelial cells, myocardial cells, proximal tubular cells of the kidney, and nerve cells^6,9^. After viral infection, the coronavirus S protein forms surface protrusions that bind to the ACE2 receptor, followed by virus particle-host membrane fusion, mediating viral entry into host cells that causes viral infection^3,4,8,10,11^. The SARS-CoV-2 major protease (MPRO), also known as 3C-like protease (3CLPro)^12^, is the main protease involved in viral replication. During viral replication, MPRO processes the viral polyprotein synthesized using the host cell translation machinery, producing a functional and active viral replication complex in the host cell that triggers various reactions throughout the body^13^. Therefore, MPRO provides an attractive target for coronavirus inhibitors^14^.

Due to the lack of specific antiviral therapy, the main treatment strategy for coronaviruses is symptomatic supportive treatment^15^. Traditional Chinese medicine (TCM) was widely used with good efficacy in the 2003 SARS outbreak and the 2019 coronavirus disease^16–18^. Andrographolide and baicalin are the active ingredients of the TCMs *andrographis paniculata* and *scutellaria baicalensis*. Many studies have explored their mechanisms in preventing and treating coronaviruses, including the ability to inhibit the activity of the SARS 3CLPro enzyme, inhibit coronavirus replication, inhibit the interaction between coronavirus S protein and ACE2, and inhibit virus cell entry, adsorption, and penetration^19^; however, there are currently no studies on their synergistic use. Therefore, this study investigated the SARS-CoV-2 antiviral mechanisms of synergistic andrographolide and baicalin treatment in vitro and in vivo.

## Results

### Andrographolide and baicalin synergistic treatment reduced ACE2 protein levels in the body

**Figure 1:**
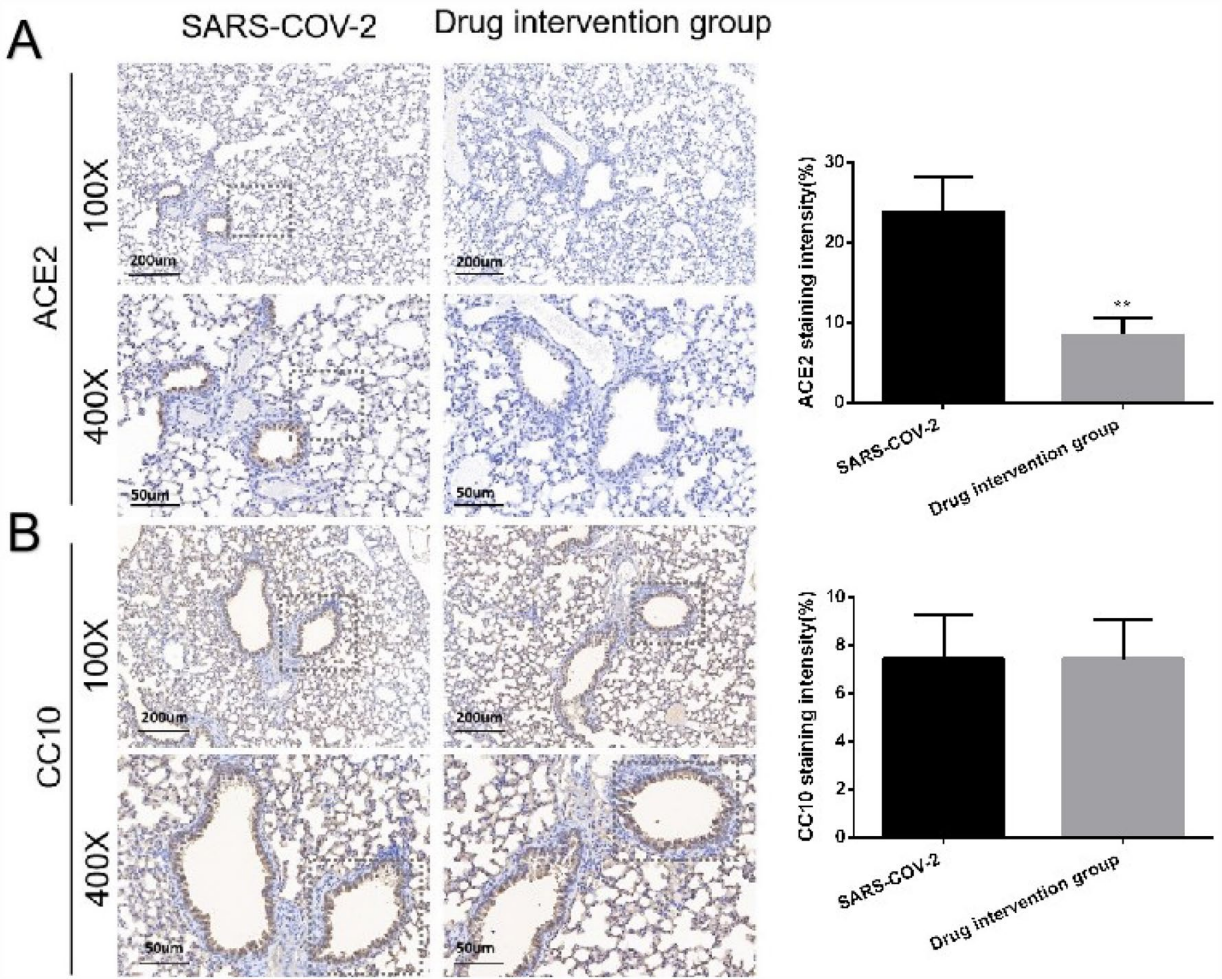
Staining of pathological tissues of mouse lung tissues ACE2 and CC10. SARS-COV-2: mice infection with a pseudovirus; Drug intervention group: Andrographolide and baicalein intervene in infected mice. The scale bars of 100 × and 400 × magnification in the panels represent 200 μm and 50 μm, respectively. *P < 0.05, **P < 0.01 SARS-COV-2 compared with Drug intervention group.

Positive staining for human ACE2, as the cellular receptor of SARS-CoV-2 virus, was mainly present in the lung epithelium, whereas CC10 protein was present in the secretory granules of non-cilia, non-serous, non-mucosal columnar cells of bronchial trees. CC10 is an endogenous anti-inflammatory factor expressed and secreted by Clara cells, which has a protective effect in inflammatory airway diseases. We examined the effect of andrographolide treatment in combination with baicalin on ACE2 and CC10 levels in lung tissue after infection with SARS-COV-2 from in vivo assays. After transgenic mice were infected with SARS-CoV-2 for 24 h, andrographolide was combined with baicalin for gavage, and immunohistochemical analysis of lung tissue was performed after 48 h. The results showed that the epithelial cells of lung tissue in the SARS-CoV-2 group exhibited with cell depletion, solidification, thickening of alveolar walls, collapse of alveolar space, and loss of airway structure (Fig. 1). Before and after drug administration, the ACE2 protein level decreased while no significant changes were observed in CC10 (Fig. 1). Compared with the SARS-CoV-2 group, the expression of ACE2 in the lung tissue of the drug intervention group mice was significantly reduced (**P < 0.01). However, there was no statistically significant difference in CC10 expression in lung tissue between the two groups.

### Andrographolide and baicalin inhibited the binding of ACE2 to Sprotein

**Figure 2:**
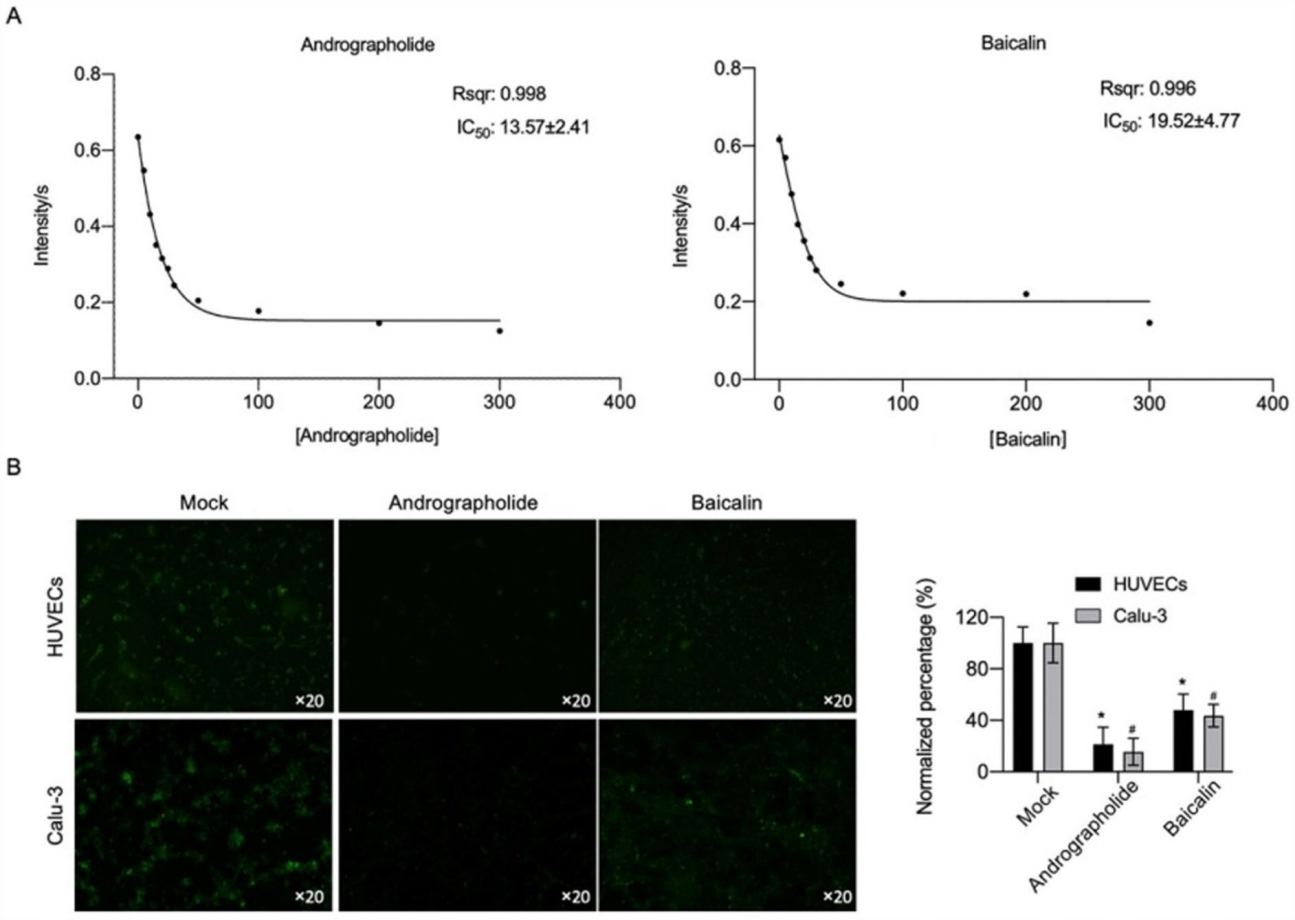
Andrographolide and baicalin inhibited the binding of ACE2 to Sprotein. (**A**) Dissociation constants of andrographolide and baicalin on ACE2 receptor and S protein binding, respectively. The coefficients of determination in this regression model were 0.998 and 0.996, with IC50s of 13.57 ± 2.41 and 19.52 ± 4.77 µg/mL. (**B**) Immunofluorescence detection: HUVECs expressed SARS-COV-2 S protein and Calu-3 expressed ACE2 receptor. Comparing the microscopic expression of S protein and ACE2 in cells infected with the virus before and after drug treatment, P < 0.05.

We investigated the effects of andrographolide and baicalein on the binding activity of the S protein in HUVEC cells and the ACE2 receptor in calu-3 cells. The IC50s of andrographolide and baicalin were 13.57 ± 2.41 and 19.52 ± 4.77 μg/mL, respectively, and we selected integer concentrations of 10 ug/mL and 25 ug/mL for follow-up experiments (Fig. 2A). The results showed that andrographolide and baicalein inhibited binding between ACE2 receptors and S proteins (Fig. 2A). It was also observed that the addition of andrographolide and baicalin decreased the levels of S protein and ACE2 protein in cells compared to the Mock group (Fig. 2B). These results suggested that andrographolide and baicalein may inhibit the levels of S proteins and ACE2 receptors, thereby affecting their binding to play an antiviral role.

### Andrographolide and baicalin synergistic therapy reduced ACE2 protein levels in vitro

**Figure 3:**
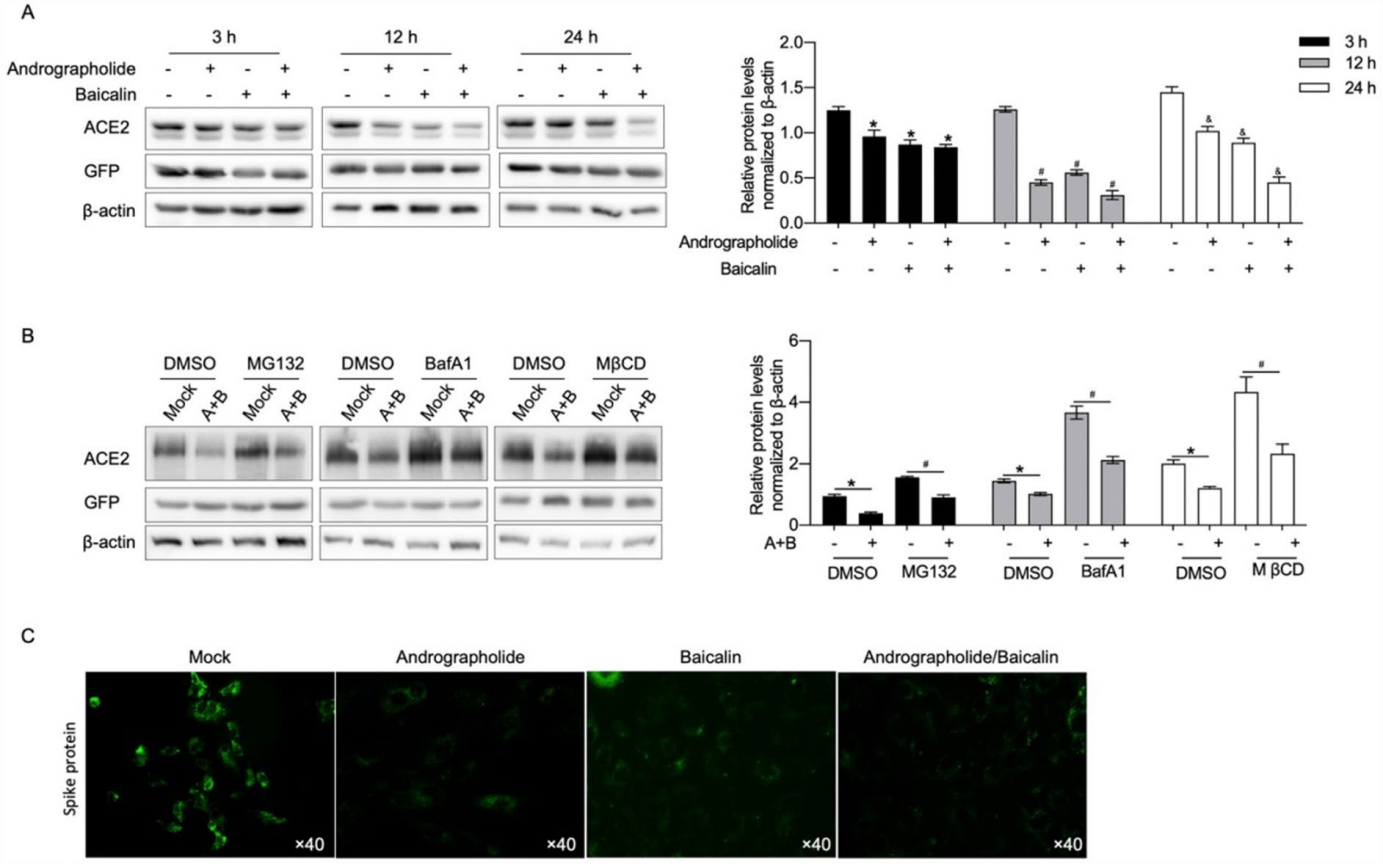
Andrographolide and baicalin decreased the expression of ACE2 protein. (**A**). ACE2 expression after 3 h, 12 h and 24 h of combined intervention of andrographolide and baicalin synergistically in cells infected with SARA-COV-2, *P < 0.05. (**B**) Drug intervention in the expression of ACE2 in cells infected with SARA-COV-2 under the conditions of adding endocytosis inhibitors, proteasome inhibitors, *P < 0.05. (**C**). Immunofluorescence assay. Comparison of microscopic expression of Spike protein before and after drug administration by immunofluorescence. A: andrographolide; B: bacalin; GFP: Green Fluorescent Protein, label cells; β-actin: internal reference.

Next, the effects of andrographolide and baicalein on ACE2 protein level in cells infected with SARS-CoV-2 were measured.. We used 10 μg/mL andrographolide and 25 μg/mL baicalin to intervene in the cells, and observed the expression of ACE2 in the cells after 3 h, 12 h, and 24 h. The researchers observed a decrease in ACE2 expression after intervening cells with andrographolide or baicalin alone; However, ACE2 expression decreased significantly after the combination of the two (Fig. 3A). This may indicate that the synergistic effect of the two is better than that of the two alone. In addition, cells were pretreated with DMSO, MG132 (proteasome inhibitor), bafilomycin A1 (BafA1, lysosomal inhibitor), and methyl-β-cyclodextrin (MβCD, lipid raft inhibitor) to maintain cell stability, and drugs were added after infection with SARS-CoV-2. The results showed that andrographolide and baicalin combined intervention reduced the ACE2 content compared with the Mock group in the presence of inhibitors (Fig. 3B)—indicating a decrease in ACE2 synthesis. Subsequently, green fluorescent protein was used for cell labeling and immunofluorescence to detect the S protein. The results showed that compared with the Mock group, the S protein level was significantly reduced after intervention with andrographolide and baicalein, but their synergistic effect was more pronounced. We suggest a potential mechanism by which these two compounds reduce the adhesion of pseudoviruses to cells (Fig. 3C).

### Synergistic andrographolide and baicalin treatment reduced S protein-induced inflammatory responses in mice

Interleukin-6 (IL-6) and tumor necrosis factor alpha (TNF-α) are cytokines that promote the progression of inflammation during the onset of infection^20,21^. These two are the most abundant cytokines detected in the plasma of patients with COVID-19, especially in the acute phase^22^, and is thought to be a host defense response to viral infection^23^. However, the presence of high levels of pro-inflammatory cytokines damages the energy metabolism of mitochondria, leading to cell dysfunction and organ failure^24^. Furthermore, lipopolysaccharides (LPSes) are bacterial endotoxins that cause cytokine storms by activating monocytes and macrophages to produce high levels of pro-inflammatory cytokines, often used for inflammatory modeling. Here, andrographolide and baicalein were given to mice by intragastric administration for one week, followed by nasal drops with LPS or pseudovirus, and then continued intragastric administration for one week. Finally, bronchoalveolar lavage fluid was obtained to detect IL-6 and TNF-a by enzyme-linked immunosorbent assay.

**Figure 4:**
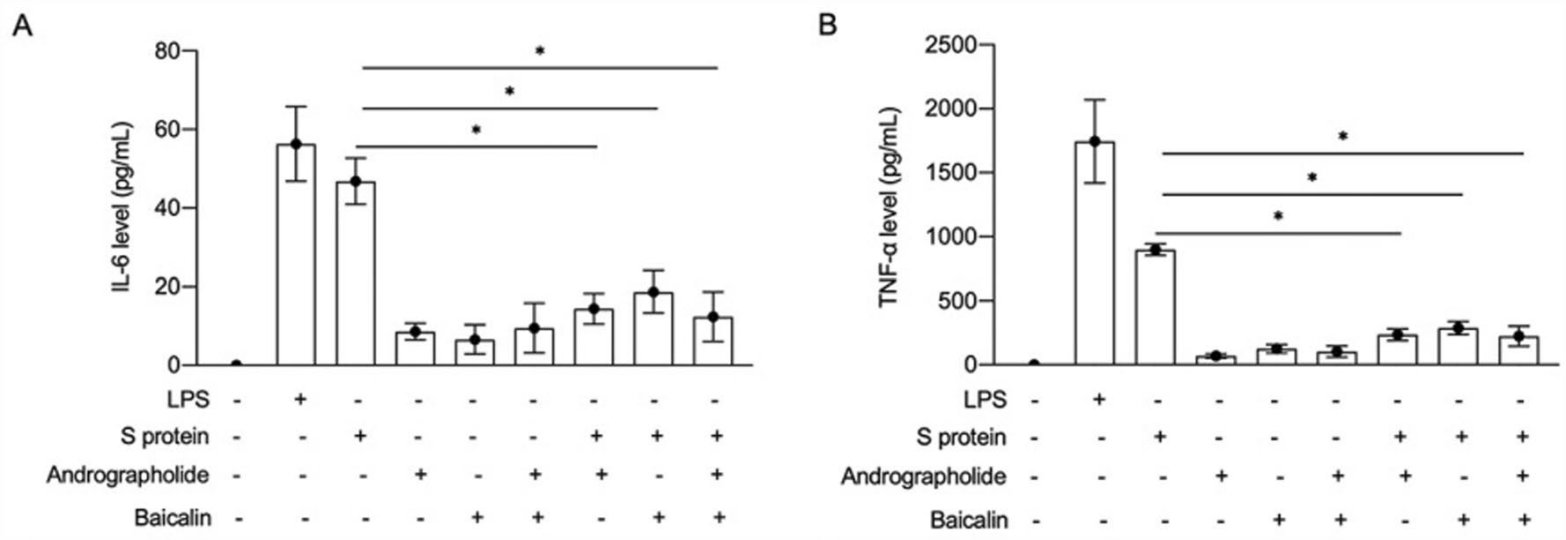
Andrographolide combined with baicalin reduced S protein-induced inflammatory response in mice. Detection of IL-6 (**A**) and TNF-α (**B**) expression by ELISA on mouse alveolar lavage fluid under different conditions of LPS, S protein, andrographolide and baicalin, *P < 0.05.

The results showed that LPS, S protein, andrographolide and baicalin all increased the expression of cytokines IL-6 and TNF-α, while the expression induced by LPS and Sprotein protein increased significantly. Moreover, compared to the two drugs alone, andrographolide in combination with baicalin significantly inhibited the levels of IL-6 and TNF-α in S-protein-induced inflammatory mice (Fig. 4). These results suggest that andrographolide and baicalin inhibited cytokine storms and reduced inflammatory responses in mice by reducing the release of IL-6 or TNF-α.

The results showed that andrographolide or baicalin alone reduced the levels of IL-6 and TNF-α in mice with S protein-induced inflammation. In addition, andrographolide in combination with baicalin significantly inhibited the levels of IL-6 and TNF-α in mice with S protein-induced inflammation (Fig. 4). These results suggested that andrographolide and baicalin had a synergistic effect that inhibited the cytokine storm in mice and reduced the inflammatory response by reducing the release of IL-6 or TNF-α.

### Andrographolide, but not baicalin, inhibited the activity of SARS-CoV-2 MPRO

**Figure 5:**
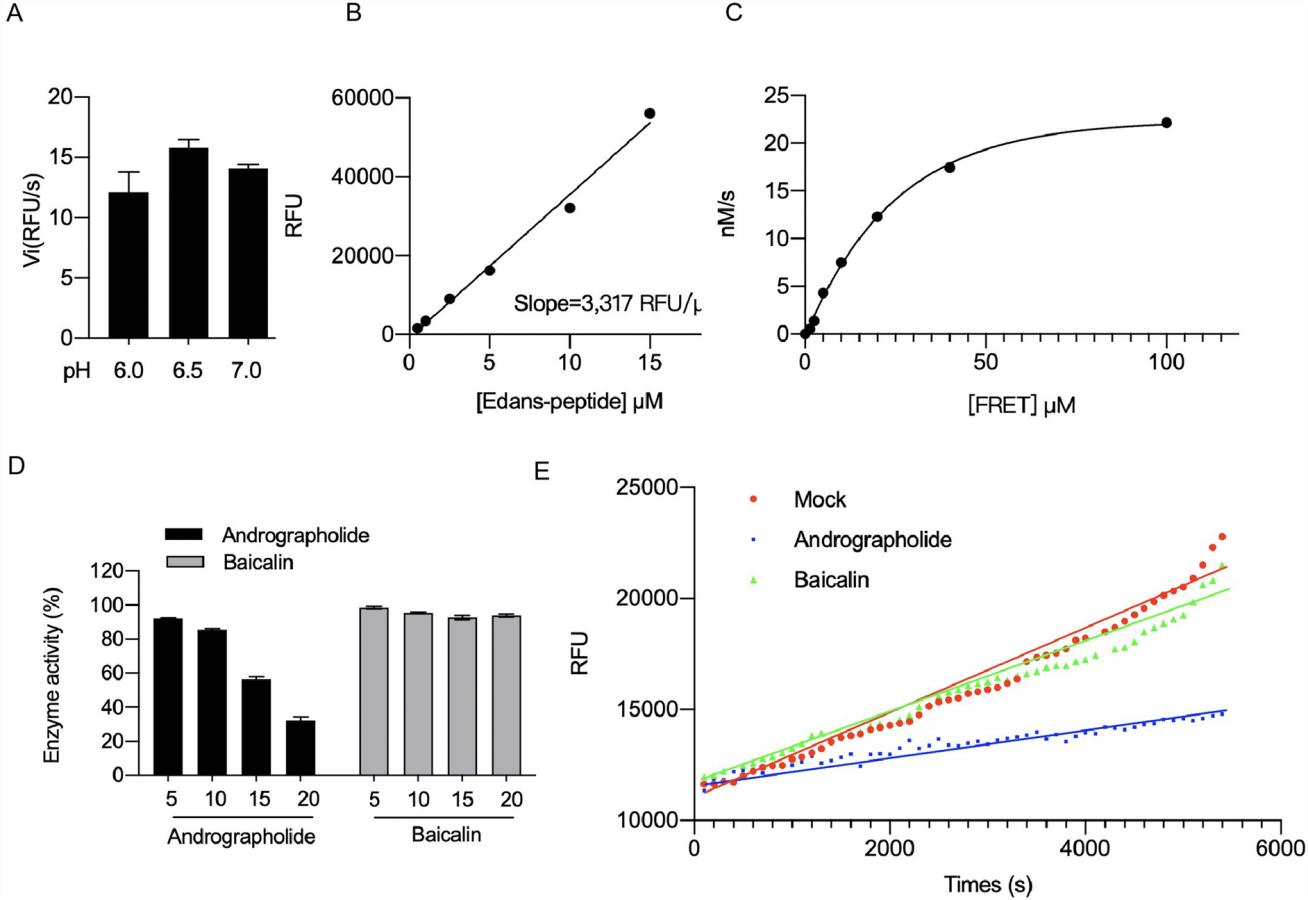
Andrographolide inhibited the activity of SARS-CoV-2 Mpro. (**A**) PH value suitable for the growth of the Mpro. (**B**) The Mpro decomposition kinetic curve was observed at pH 6. (**C**) The Mpro synthesis kinetic curve was observed at a pH of 6. (**D**) Change in the Mpro activity after addition of drug. (**E**) Drug and time dependence of the Mpro activity.

The effects of andrographolide and baicalin on MPRO activity was investigated. MPRO is a crucial protein that mediates viral replication and transcription, making it a promising target for COVID-19 treatment. A proteinase activity assay was used to measure MPRO activity at an optimal pH of 6.5 (Fig. 5A). A linear increase in enzyme activity was observed with an edans-peptide concentration up to 100 uM at pH 6.5 (Fig. 5B, C). Next, the effects of andrographolide and baicalin on MPRO activity and substrate degradation was investigated. The results showed a gradual decrease in enzyme activity with increasing concentrations of andrographolide, while baicalin had no significant effect (Fig. 5D). Furthermore, andrographolide showed time- and dose-dependent inhibition of enzyme activity compared with the control group, while baicalin had no significant effect over time (Fig. 5E). These findings suggest that andrographolide reduced MPRO activity in a time- and dose-dependent manner, potentially preventing viral entry, while baicalin had little to no effect on MPRO activity.

## Discussion

Coronaviruses has caused significant public health crises, including that of the 2003 SARS outbreak, 2012 MERS, and the ongoing COVID-19 pandemic caused by SARS-CoV-2^18^. The COVID-19 pandemic has resulted in the devastating loss of life and a profound economic impact globally, with over 1.9 billion confirmed cases and over 190,000 deaths as of February 6, 2023^25^. Although the global situation has improved significantly, patients with asymptomatic COVID-19 can still transmit the virus for over 14 days, and up to 80% of patients may experience long-term symptoms^25^. Complications of neo-coronary pneumonia have been described in roughly 50 different ways, occurring in the heart, lungs, nervous system, and metabolic system^26^.

Patients with severe COVID-19 may develop cytokine storm syndrome, which increases the risk of developing fatal acute respiratory distress syndrome^27^. With new variants of coronavirus still possible, it is essential to prevent its spread, and there are currently no effective antiviral drugs targeting SARS-CoV^1^. This global health crisis has led people to explore the use of TCM as antiviral treatment^28^.

Andrographolide and baicalin are compounds derived from TCM, and have been used in clinical practice for their anti-inflammatory and antiviral properties^23,28^. Nevertheless, no studies have investigated their synergistic effects. The ACE2 receptor promotes the entry of SARS-CoV and SARS-CoV-2 into host cells, making it a crucial target for antiviral therapy^29,30^. Here, both andrographolide and baicalin could effectively reduce the expression of ACE2 protein levels in S protein-induced mouse models. This decrease in ACE2 levels may be related to the decrease in viral cell adhesion following drug intervention.

LPSes are a type of bacterial endotoxin that can cause a cytokine storm by activating monocytes and macrophages to produce high levels of pro-inflammatory cytokines (such as TNF-α, IL-6, and IL-1β), leading to systemic inflammation and even death^31,32^. LPS is commonly used in constructing classic inflammation research models, including acute lung injury, sepsis, and acute respiratory distress syndrome. Therefore, LPS is also widely used in the modeling of COVID-19-related cytokine storms^31,33^. Rapid viral replication, cell damage, and virus-induced ACE2 reduction or shedding can all trigger an inflammatory response in the body^34^. IL-6 and TNF-α are elevated in the blood and diseased tissue of patients with severe COVID-19 and are key cytokines for the development of cytokine storms^35,36^. In this study, LPS was used to establish an inflammation-related model, and compared with the S protein-induced inflammatory model, it was found that synergistic andrographolide and baicalin treatment could effectively inhibit the increase of the S protein-induced inflammatory factors IL-6 and TNF-α, reduce lung inflammation, and inhibit cytokine storm.

The invasion of host cells by SARS-CoV-2 begins with the binding of the viral S protein to the human ACE2 receptor, which is achieved by cleaving the receptor-binding domain on S1 during the process of viral binding and entry^37^. This process of entering host cells depends on the interaction between complex sugars (polysaccharides) on the viral surface and host cells through glycans, which is necessary for SARS-CoV-2 replication^38^. In this study, baicalin and andrographolide prevented the attachment of the viral S protein and ACE2 receptor. This suggests that baicalin and andrographolide reduce the docking of the virus on host ACE2 and viral entry. In addition, andrographolide dose-dependently inhibited the activity of MPRO—indicative that andrographolide may inhibit SARS-CoV-2 replication^39,40^.

In summary, the study found that synergistic andrographolide and baicalin treatment reduced the content of ACE2, showed significant binding affinity to the S protein of SARS-CoV-2 and ACE2 receptor, reduced the adhesion of the virus to ACE2, inhibited their complex-formation, prevented viral entry, and reduced pro-inflammatory cytokines such as IL-6 and TNF-α to reduce the inflammatory response caused by the virus—thus reducing cytokine storms. Simultaneously, andrographolide inhibits the activity of MPRO to inhibit viral replication, consistent with previous research findings^23,28,39,41,42^. All evidence suggests that synergistic baicalin and andrographolide treatment exhibits various anti-SARS-CoV-2 which provide important insight for the future development of anti-coronavirus therapies.

## Methods

The experiment was conducted in the experimental animal center of Chengdu University of traditional Chinese medicine and submitted to the Institutional Ethics Review Committee for approval (grant no.: 2020QKL-001). This study was conducted in strict accordance with the recommendations of the guide for the care and use of laboratory animals issued by the Ministry of science and technology of China. All experiments complied with the ARRIVE.

### Packaging of shRNA lentivirus targeting ACE2 protein

Culture 293 T cells in a 10 cm culture dish. After the cells grow to confluence, digest them, count and transfer about 600,000–800,000 cells to each well of a 6-well plate and incubate in a cell culture incubator. When the cells attach and grow to more than 60% confluence, replace the medium with DMEM containing no antibiotics and 10% FBS. Prepare the target plasmid ACE2, helper plasmid, lip3000 and p3000, and opti-MEM medium. Take two sterile 1.5 ml EP tubes: add 125 μl of opti-MEM and 3.75 μl of lip3000 to tube 1. Add 125 μl of opti-MEM and 5 μl of p3000 to tube 2. Add the plasmid DNA to tube 2 according to the ratio of target plasmid: helper plasmid (PSPAX2:PMD2G) = 4:3:1, with a total amount of 2.5 μg. Mix the liquid in tube 1 and tube 2, let it stand for 15 min, and then drip it into the 6-well plate. Discard the first culture medium after 12 h, record this time as 0 h, and collect the medium at 24 h, which is the first viral solution. Collect the second viral solution after 48 h. Mix the two viral solutions collected and filter through a 0.45 μm filter to obtain packaged lentivirus. Store it at -80 °C after dividing into aliquots.

### Preparation of alveolar lavage fluid induced by andrographolide and baicalin for the treatment of LPS or pseudovirus nasal drops

Specific pathogen Free grade Sprague Dawley adult mice. Under optimal conditions of 22 ± 2 °C and 50–70% humidity, animals are housed in specific pathogen-free animal chambers for 12 h with a light–dark cycle for 12 h and fed standard mouse chow and water. Ten mice are randomly divided into two subgroups. This is followed by a model group induced with LPS or pseudovirus nasal drops and a drug intervention group treated with andrographolide and baicalin. The model group was induced by LPS or pseudovirus nasal drops for one week; in the experimental group, andrographolide and baicalin 10 mg/d were gavaged for 1 week. Finally, alveolar lavage fluid was obtained for Elisa testing.

### Transgenic mice production of transgenic mice using DNA protoplast microinjection

Exogenous DNA is injected into the nucleus of the fertilized eggs by microinjection, and the injected DNA is integrated into the genome of the fertilized eggs of the mice and stably passed on to the offspring(Guangzhou Saiye Company). Mice were divided into 2 groups, including control and drug groups, with 4 mice in each group. Staining for the lung marker ACE2 and the lung epithelial cell injury marker CC10 in transgenic mice was performed. Immunohistochemical analysis of paraffin-embedded mouse lung tissue slides was performed using ACE2 (Affinty, AF5165) and CC10 antibody (Proteintech, 10,490–1-AP) under 20 × and 40 × lenses.

### Packaging of Omicron pseudovirus

Omicron pseudovirus was purchased from Nanjing jinsirui Science & Technology Biology Corp[(lentivirus packaging (SC1394-VP)].

### Enzyme-linked immunosorbent assay ( ELISA) detection protocol

Take 2 mL of lung lavage solution from each group of mice, centrifuge at 710 rcf for 5 min with a centrifugal radius of 10 cm, and collect the supernatant. Detect the levels of IL-6(Beyotime, Mouse IL-6 ELISA Kit), and TNF-a(Beyotime, Mouse TNF-α ELISA Kit) according to the instructions of the kit. Add 100 μl working solution to the corresponding wells of the plate, incubate for 1.5 h, remove the liquid from the plate and add 100 μl biotinylated antibody working solution, incubate for 1 h, remove the liquid from the plate and wash with PBS. Then add 100 μl HRP conjugate working solution, incubate for 0.5 h, remove the liquid from the plate, wash with PBS, add 90 μl substrate solution, and after 15 min, add 50 μl stop solution. Detect the OD value at 400 nm wavelength and calculate the levels of IL-6, and TNF-a.

### Synthesis and expression of SARS-CoV-2 S-RBD protein (Nanjing GenScript)

The synthetic DNA sequence of S-RBD fusion protein gene was constructed into an Escherichia coli expression vector to obtain the S-RBD protein expression plasmid. The expression plasmid was transformed into E. coli competent cells for cultivation, and single colonies were selected for induction and then collected by centrifugation. The collected bacteria were sonicated in a buffer, centrifuged, washed twice in a buffer, and the precipitate inclusion bodies were obtained. The precipitated inclusion bodies were dissolved, sonicated, and centrifuged, and the supernatant was purified using a chromatography column to obtain the eluted purified protein. After adding a reducing agent, it was loaded into a dialysis bag for dialysis, and the collected supernatant after centrifugation was then the recombinant SARS-CoV-2 COVID-19 S-RBD protein.

### Immunoprecipitation

After transfection for 24–48 h, collect the cells and add an appropriate amount of cell lysis buffer (containing protease inhibitors). Place on ice for 30 min to lyse the cells. Centrifuge the cell lysate at maximum speed for 30 min at 4 °C and collect the supernatant. Take a small amount of the lysate for Western blot analysis, and add 1 μg of the corresponding antibody to the remaining cell lysate. Incubate overnight at 4 °C with slow shaking. Take 10 μl of protein A agarose beads and wash them three times with an appropriate amount of lysis buffer each time, centrifuging at 3000 rpm for 3 min. Add the pre-treated 10 μl protein A agarose beads to the cell lysate incubated with the antibody overnight and incubate at 4 °C with slow shaking for 2–4 h to couple the antibody with the protein A agarose beads. After the immunoprecipitation reaction, centrifuge the beads at 3000 rpm for 3 min at 4 °C and remove the supernatant carefully. Wash the beads with 1 ml of lysis buffer 3–4 times. Finally, add 15 μl of 2 × SDS loading buffer and boil in water for 5 min. Analyze using SDS-PAGE, Western blotting or mass spectrometry.

### Western blot

After lysis, the cell lysate was centrifuged at 12,000*g* for 10 min at 4 °C to collect protein. The protein concentration of each group was determined using a BCA protein assay kit. The extracted protein was denatured and then loaded onto an SDS-PAGE gel with equal amounts of protein. After separation by SDS-PAGE, the proteins were transferred to a PVDF membrane (PVDF Transter Membrane,Thermo). The PVDF membrane was blocked with 5% BSA solution on a shaker at room temperature for 1 h. After blocking was completed, protein bands were cut strictly according to the molecular weight range. The cut bands were added to primary antibodies ACE2 (Affinty, AF5165, 92kDa), GFP (abcam, ab84191, 27 kDa) and β-actin (abcam, ab8226, 42 kDa) according to the molecular weight range, and incubated at 4 °C overnight. The membrane was washed three times with PBS and then incubated with HRP-conjugated secondary antibody (Affinity, S0002) on a shaker at room temperature for 2 h. The membrane was then washed five times with PBS-T. Finally, protein bands were detected under an exposure meter (minichemi chemiluminescent imaging system) using ECL substrate. The images were quantitatively analyzed using Image J software.

### Real-time fluorescence quantitative PCR

Cell RNA was extracted using Trizol, and reverse transcription was performed to obtain cDNA. Primers were designed using Primer 3.0 and Oligo 6.0 software. The cDNA and primers were added to the real-time fluorescence quantitative PCR reaction system. The reaction conditions were set according to the Real-time PCR protocol: 10 min at 95 °C for pre-denaturation, 15 s at 95 °C for denaturation, 60 s at 60 °C for annealing/extension, repeated for 40 cycles. Calculate the relative levels of ACE2 gene relative to Ractin.

### Cell cycle (PI staining)

Cells were digested with trypsin containing EDTA and collected in a centrifuge tube. After centrifugation at 300 g for 5 min, the supernatant was removed and the cells were resuspended in 1 ml of pre-chilled PBS. The cells were then centrifuged again and resuspended in 750 μl of pre-chilled absolute ethanol for fixation. After washing with PBS, the cells were resuspended and treated with RNase A solution at 37 °C for 30 min. After centrifugation to remove RNA, the cells were stained with 400 μl propidium iodide staining solution. The cells were mixed slowly and thoroughly and incubated at 4 °C in the dark for 30 min. Finally, flow cytometry was used to detect red fluorescence at an excitation wavelength of 488 nm and light scattering.

### Culture of HUVEC and Calu-3 cells

The frozen cells [human lung adenocarcinnma cells(HUVEC,iCell-h110, iCell)and human lung adenocarcinoma cells,Calu-3(calu-3,CL-0054,Pricella)] were taken out from the cryotank and quickly placed into a 36–37 °C water bath, slowly shaken to allow rapid thawing within 30–60 s. After that, the cells were centrifuged at 600–800 g rcf/5 min to remove the supernatant, then resuspended in 1 ml fresh culture medium. The cells were placed in a new culture dish and added with 90% culture medium (special culture medium for HUVEC,iCell-h110-001b), 10% FBS, and 1% antibiotics. Negative for bacteria, fungi, mycoplasmas. The mixture was shaken evenly and incubated in a 37 °C 5%CO2 incubator.

### Immunohistochemical detection of ACE2, CC10 protein level

Samples were taken from mouse tissues. The tissues were embedded in paraffin and sliced (thickness of 3um), deparaffinized, dehydrated, then antigen retrieval was performed, followed by adding hydrogen peroxide positive blocking solution (Shanghai Aiyen Biotechnology Co., Ltd.), incubated for 15–20 min, then adding phosphate buffer solution (China Thermo Fisher Scientific Technology Co., Ltd.) washing 3–4 times, 3–5 min each time. Then, primary antibody ACE2 and CC10 was added, and the antibody was incubated overnight. After that, goat anti-secondary antibody was added and labeled with horseradish peroxidase (EMJ-9999, Amjay Technologies, Inc.), followed by incubation at room temperature for 60 min. Then, aminoethyl carbazole (Shanghai Jichun Industry Co., Ltd.) was added for incubation, and the cells were stained with hematoxylin–eosin, dehydrated after slicing, and finally sealed. Positive cell expression was selected in regions with higher expression levels of ACE2 and CC10 protein under a high-power microscope, and statistical analysis was performed. At some stage of the cell experiment, cells were pretreated with DMSO (the final concentration is less than 0.1%), MG132 (proteasome inhibitor, concentration 25 μM, Selleck), bafilomycin A1 (BafA1, lysosomal inhibitor, concentration 0.4 uM, Selleck), and methyl-β-cyclodextrin (MβCD, lipid raft inhibitor, concentration 4 mM, MCE). All should be used at the usual recommended concentrations in the instructions.

### Statistical analysis

Statistical analyses were carried out using Prism software (GraphPad Prism 7.0), and results were presented as mean ± standard error of mean (SEM) and single comparisons were made using unpaired two-tailed Student's t-tests. Statistical details of experiments and animal replication numbers (n) are stated in the relevant figure legends and method details. P value less than 0.05 is considered statistically significant.

### Supplementary Information

Supplementary Information 1.

Supplementary Information 2.

Supplementary Information 3.

## Data Availability

The datasets generated during and/or analysed during the current study are available from the corresponding author on reasonable request.
