# Supplementary figures and images for: Synergistic inhibition effects of andrographolide and baicalin on coronavirus mechanisms by downregulation of ACE2 protein level
Source: Sci Rep. 2024 Feb 21;14:4287. doi: 10.1038/s41598-024-54722-5 (PMC10882053; doi:10.1038/s41598-024-54722-5)

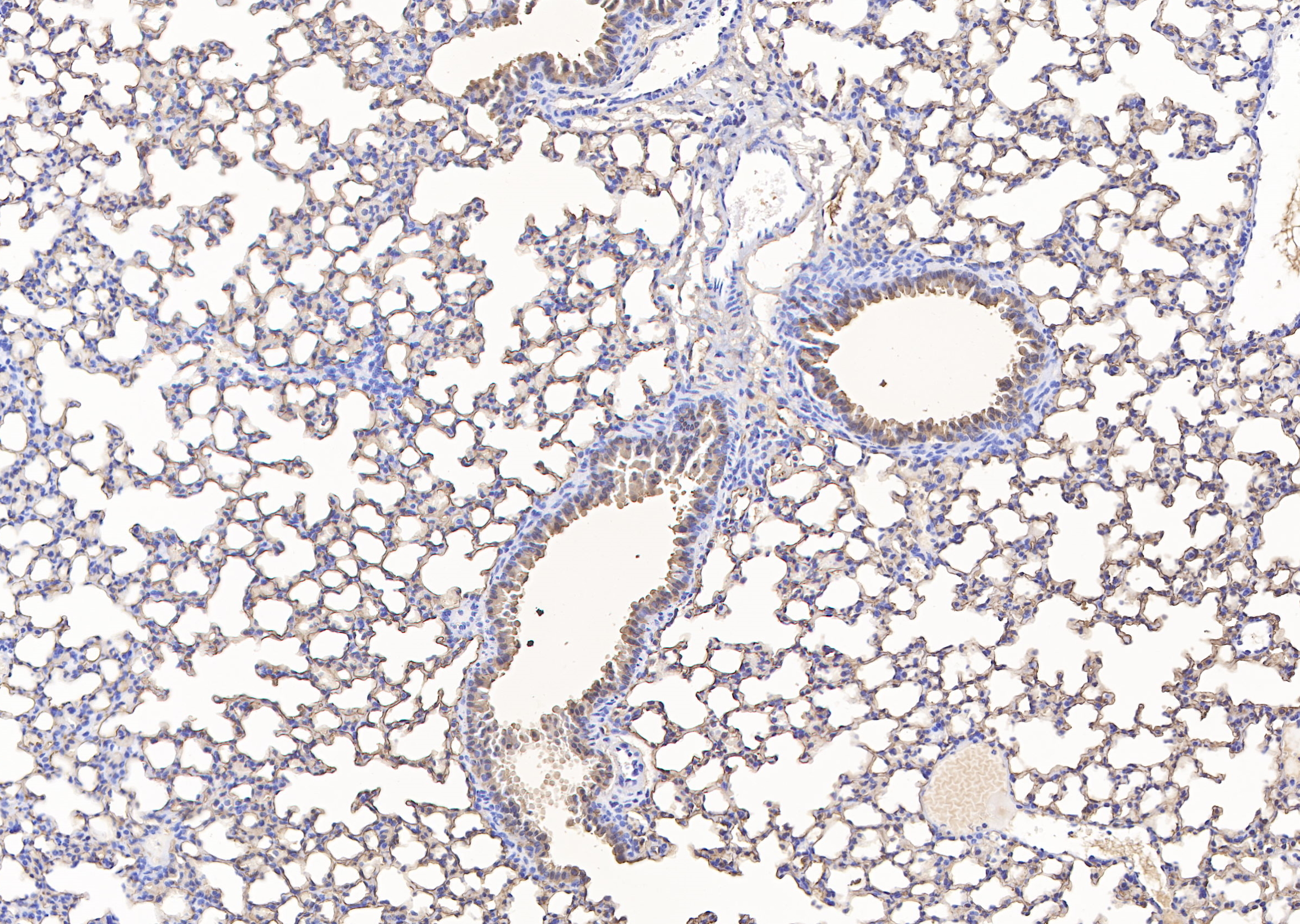

Supplement: Supplementary file 1 — Supplementary Information 1. [file 41598_2024_54722_MOESM1_ESM.zip › raw data/Figure1/Drug intervention droup CC10 100x.jpg]

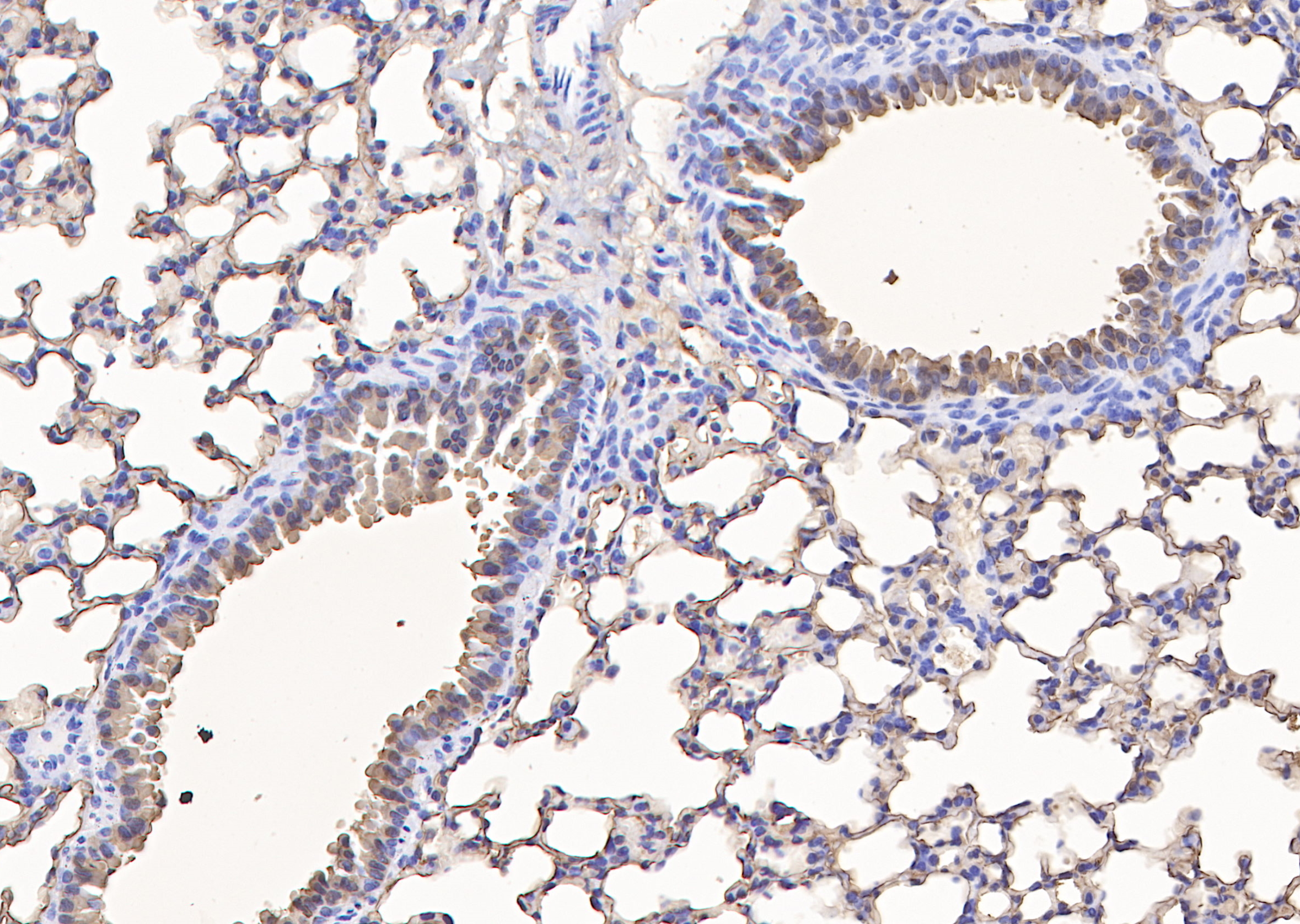

Supplement: Supplementary file 1 — Supplementary Information 1. [file 41598_2024_54722_MOESM1_ESM.zip › raw data/Figure1/Drug intervention droup CC10 400x.jpg]

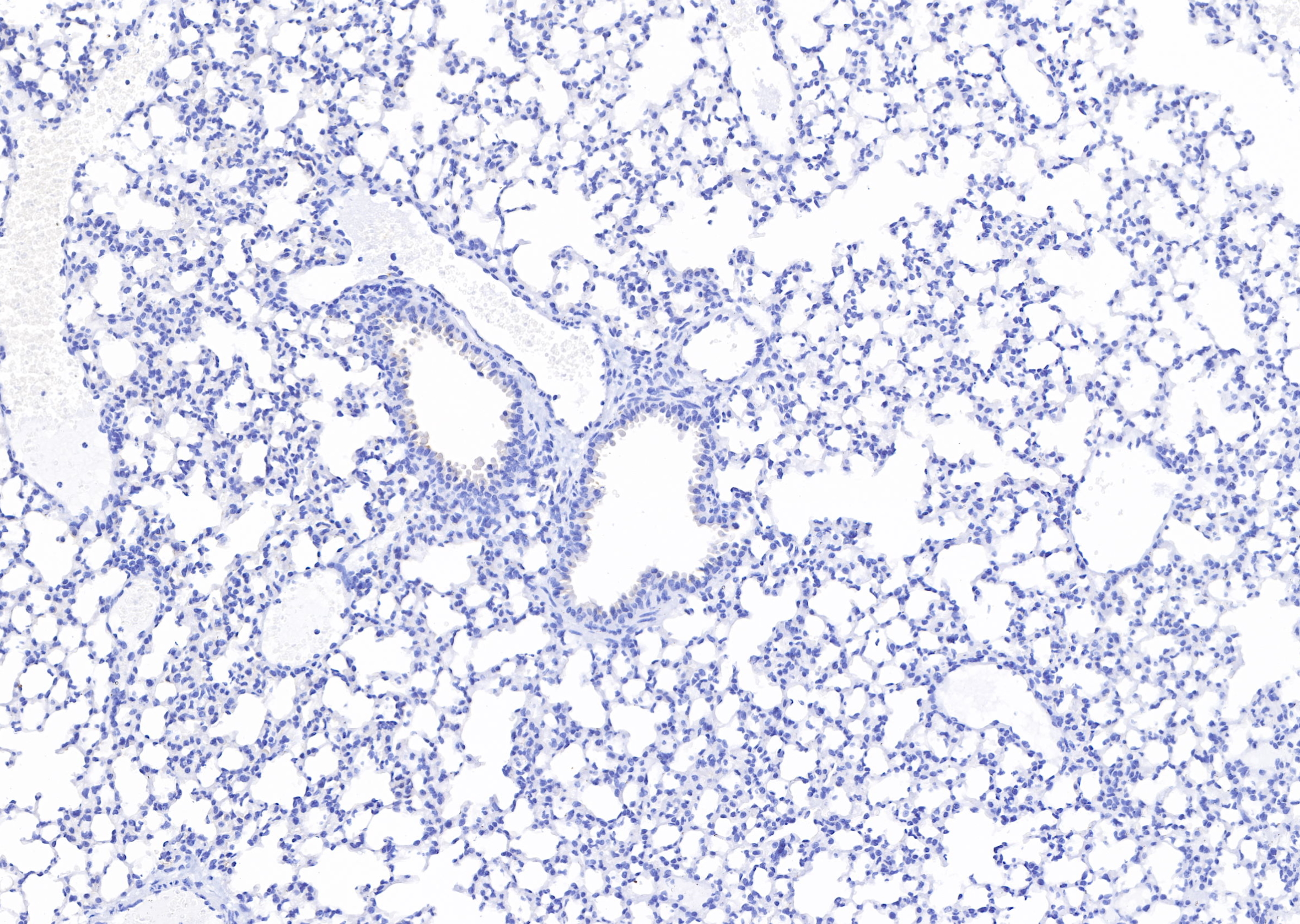

Supplement: Supplementary file 1 — Supplementary Information 1. [file 41598_2024_54722_MOESM1_ESM.zip › raw data/Figure1/Drug intervention droup ACE2 100x.jpg]

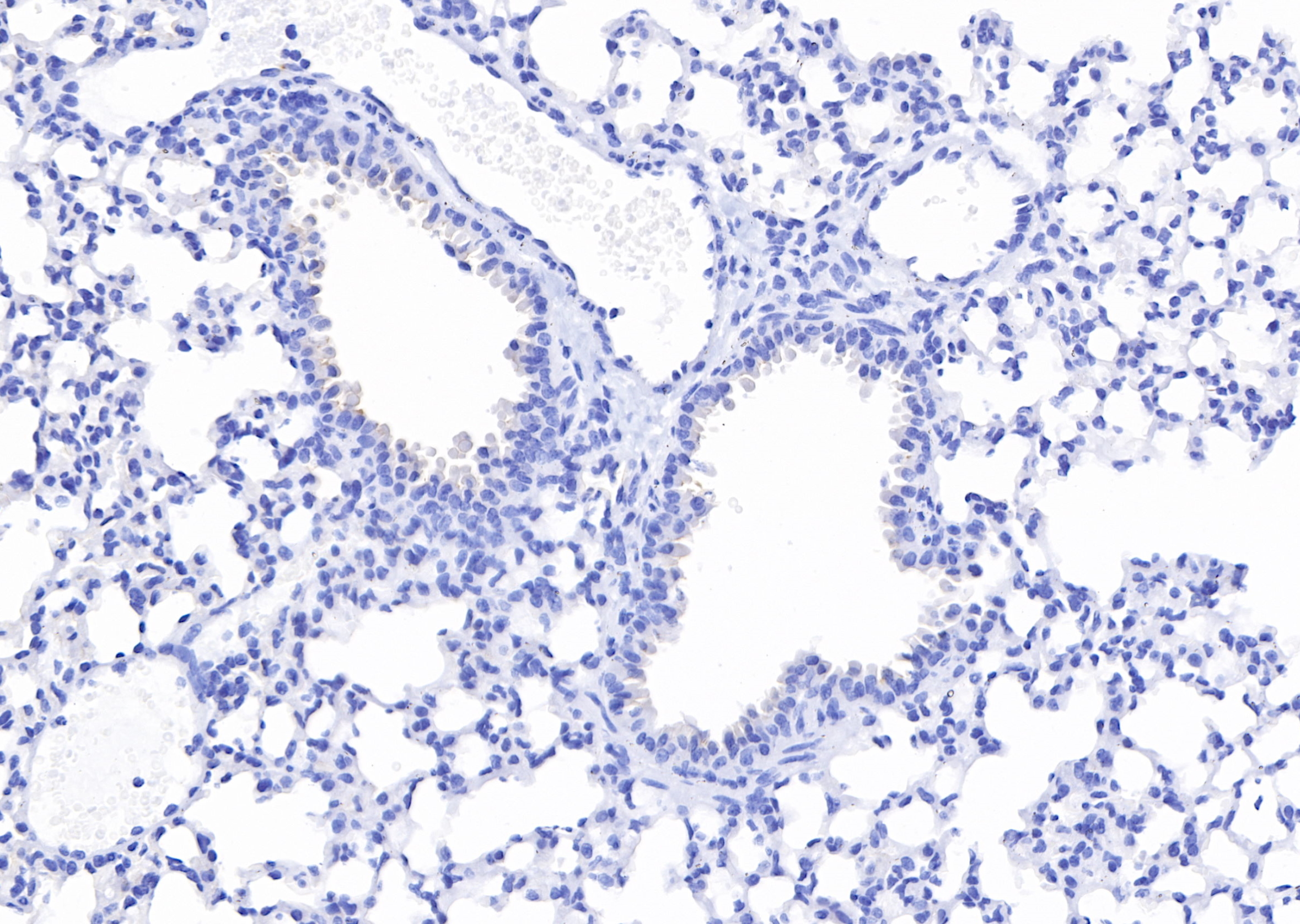

Supplement: Supplementary file 1 — Supplementary Information 1. [file 41598_2024_54722_MOESM1_ESM.zip › raw data/Figure1/Drug intervention droup ACE2 400x.jpg]

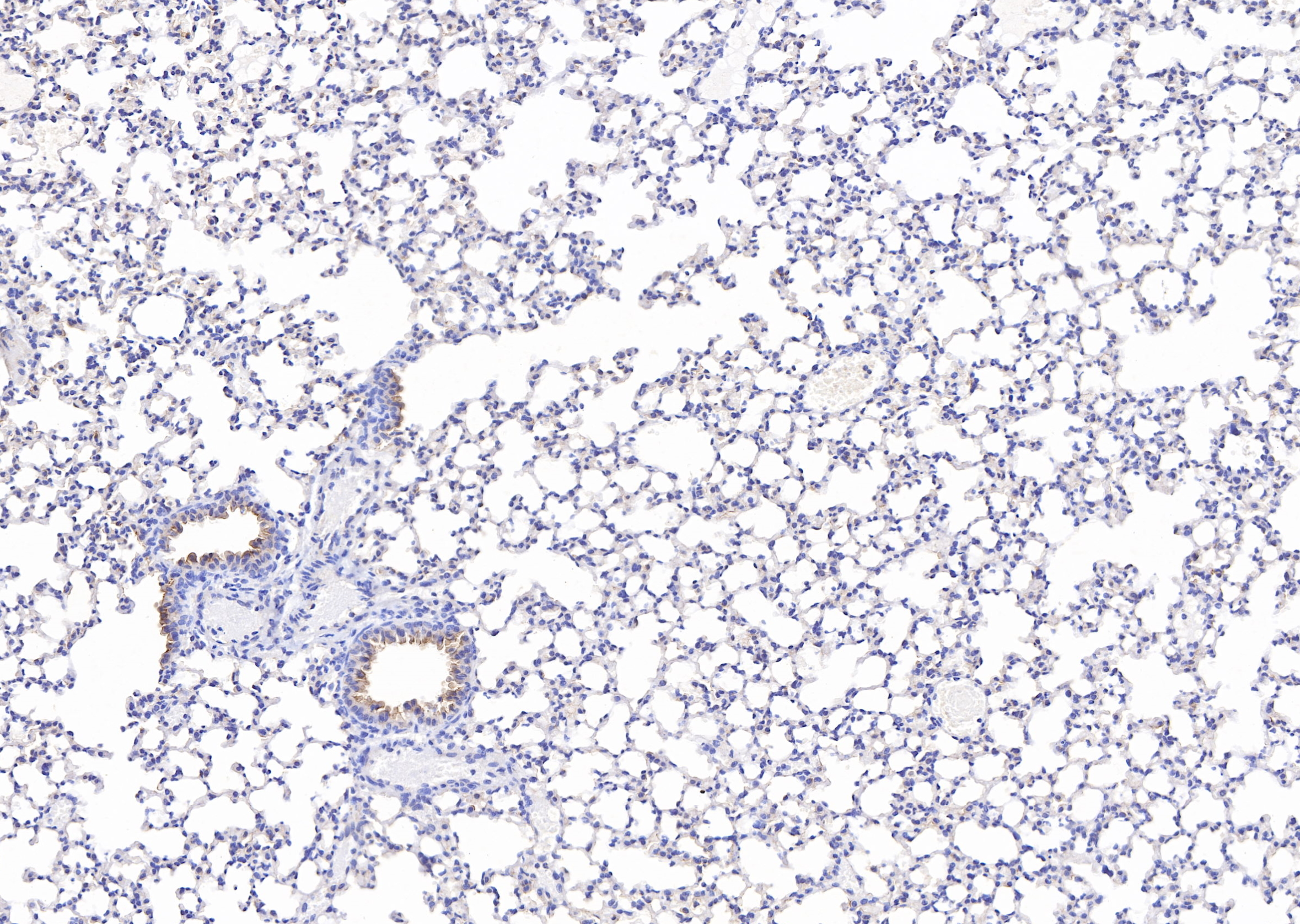

Supplement: Supplementary file 1 — Supplementary Information 1. [file 41598_2024_54722_MOESM1_ESM.zip › raw data/Figure1/SARS-COV-2 ACE2 100x.jpg]

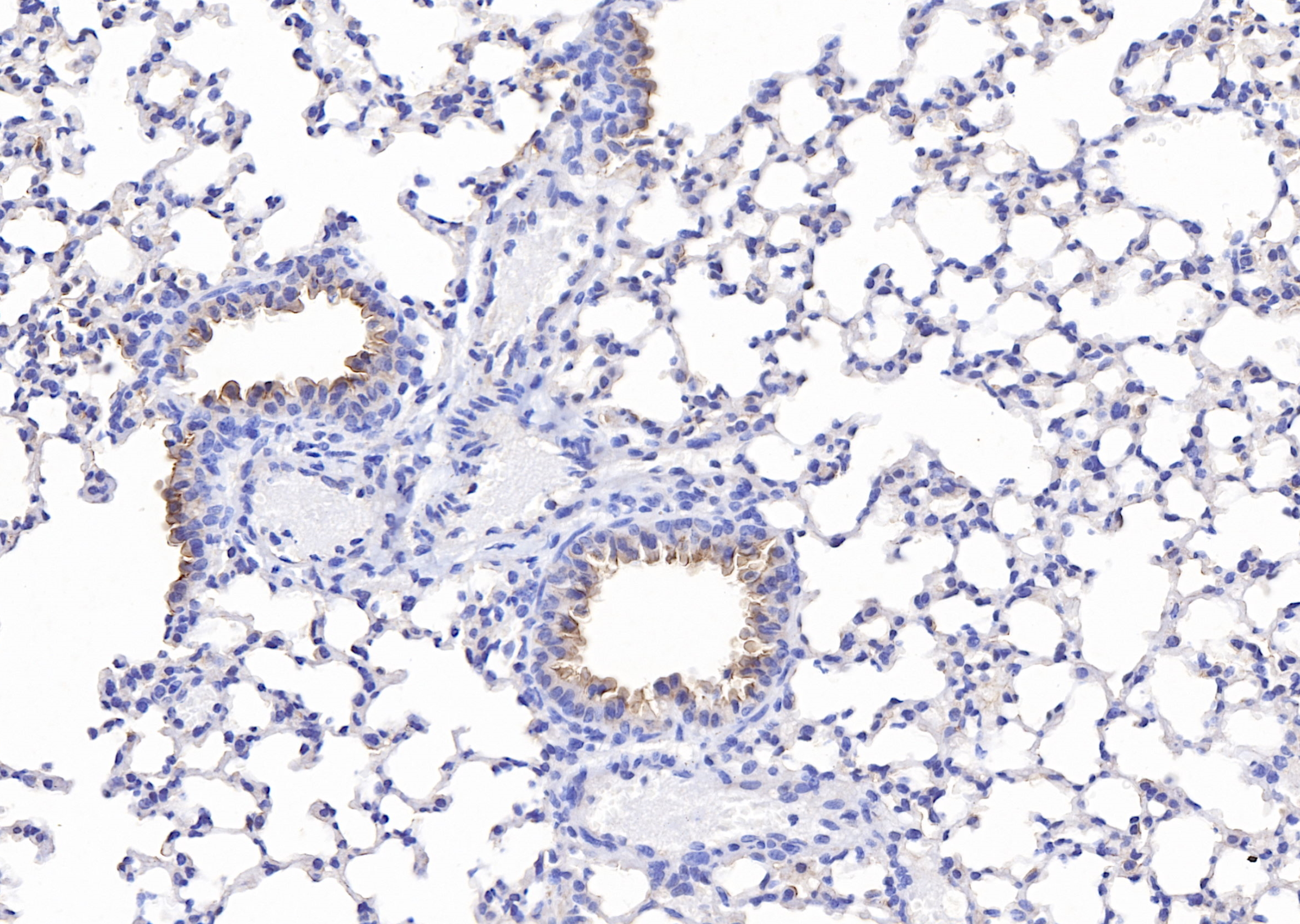

Supplement: Supplementary file 1 — Supplementary Information 1. [file 41598_2024_54722_MOESM1_ESM.zip › raw data/Figure1/SARS-COV-2 ACE2 400x.jpg]

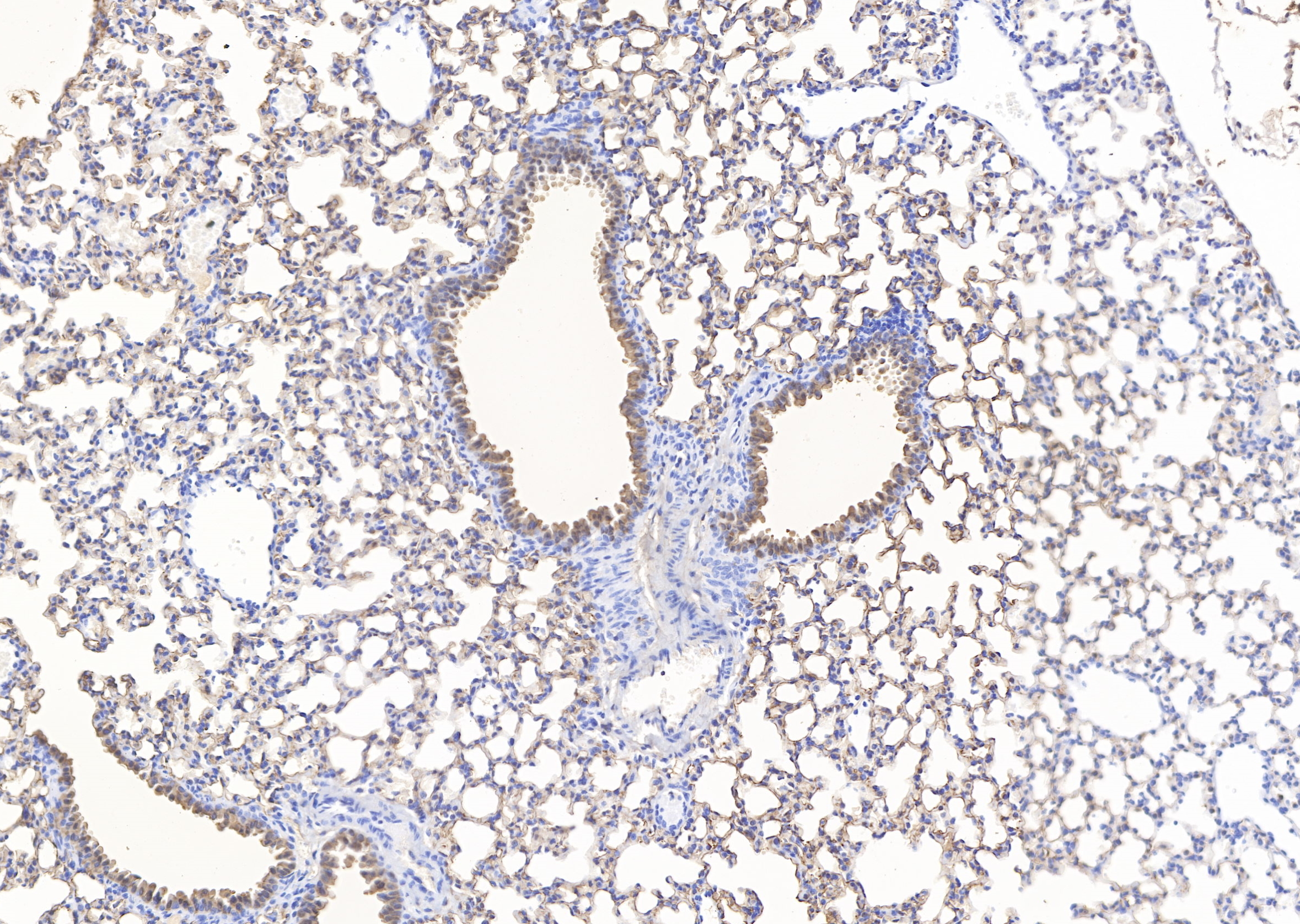

Supplement: Supplementary file 1 — Supplementary Information 1. [file 41598_2024_54722_MOESM1_ESM.zip › raw data/Figure1/SARS-COV-2 CC10 100x.jpg]

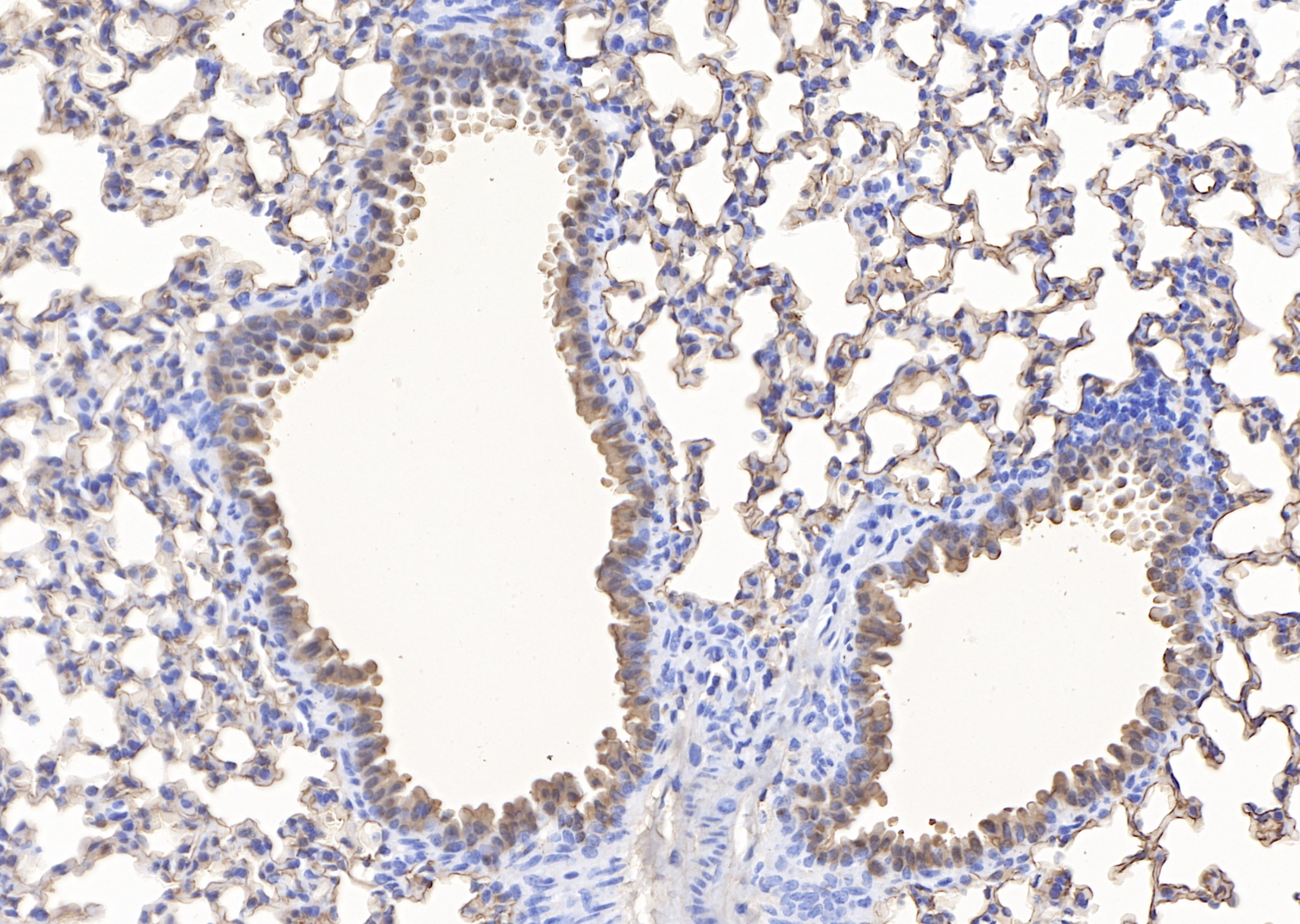

Supplement: Supplementary file 1 — Supplementary Information 1. [file 41598_2024_54722_MOESM1_ESM.zip › raw data/Figure1/SARS-COV-2 CC10 400x.jpg]

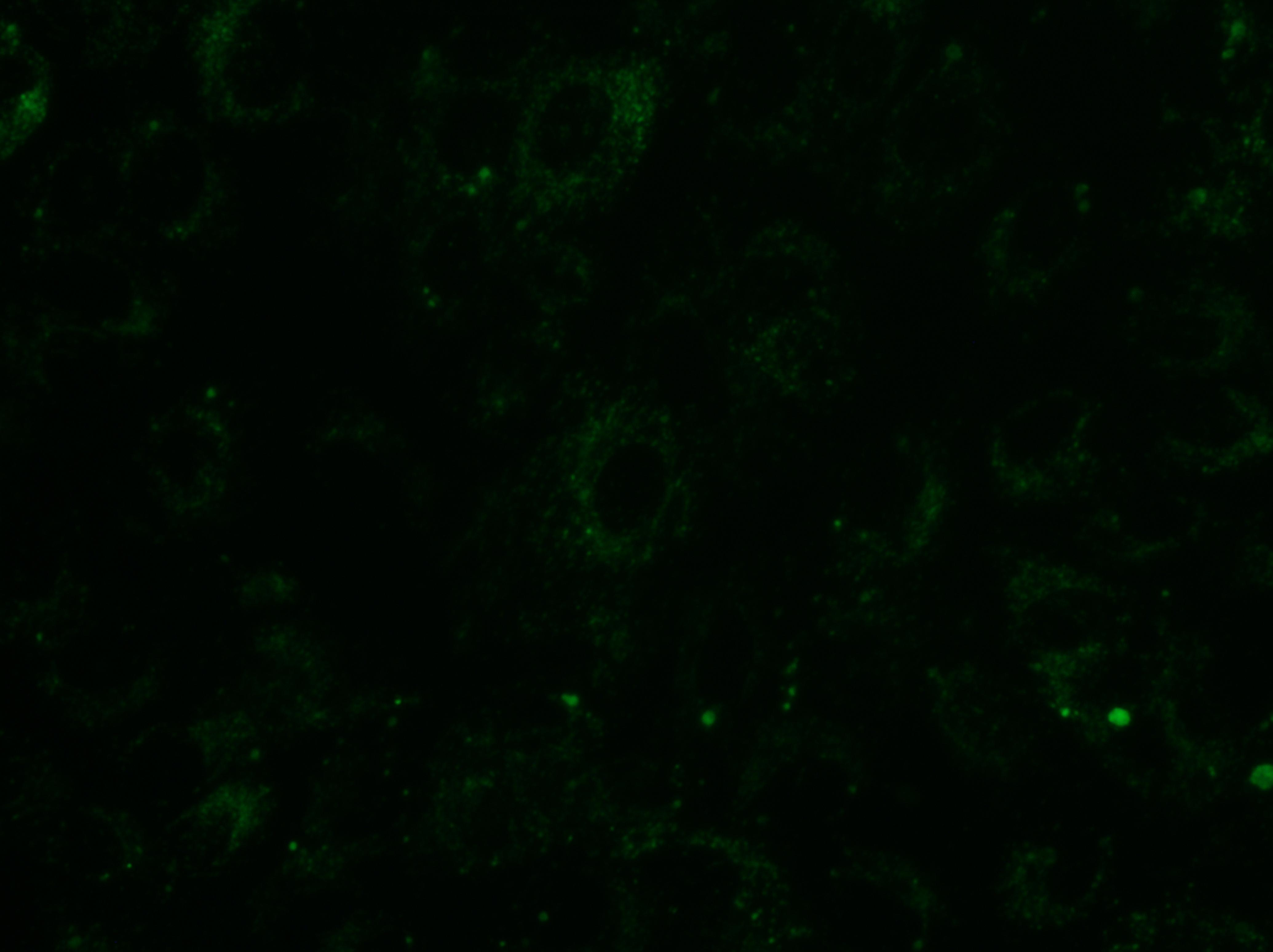

Supplement: Supplementary file 1 — Supplementary Information 1. [file 41598_2024_54722_MOESM1_ESM.zip › raw data/Figure2/Andrographolide and Bbacalin.jpg]

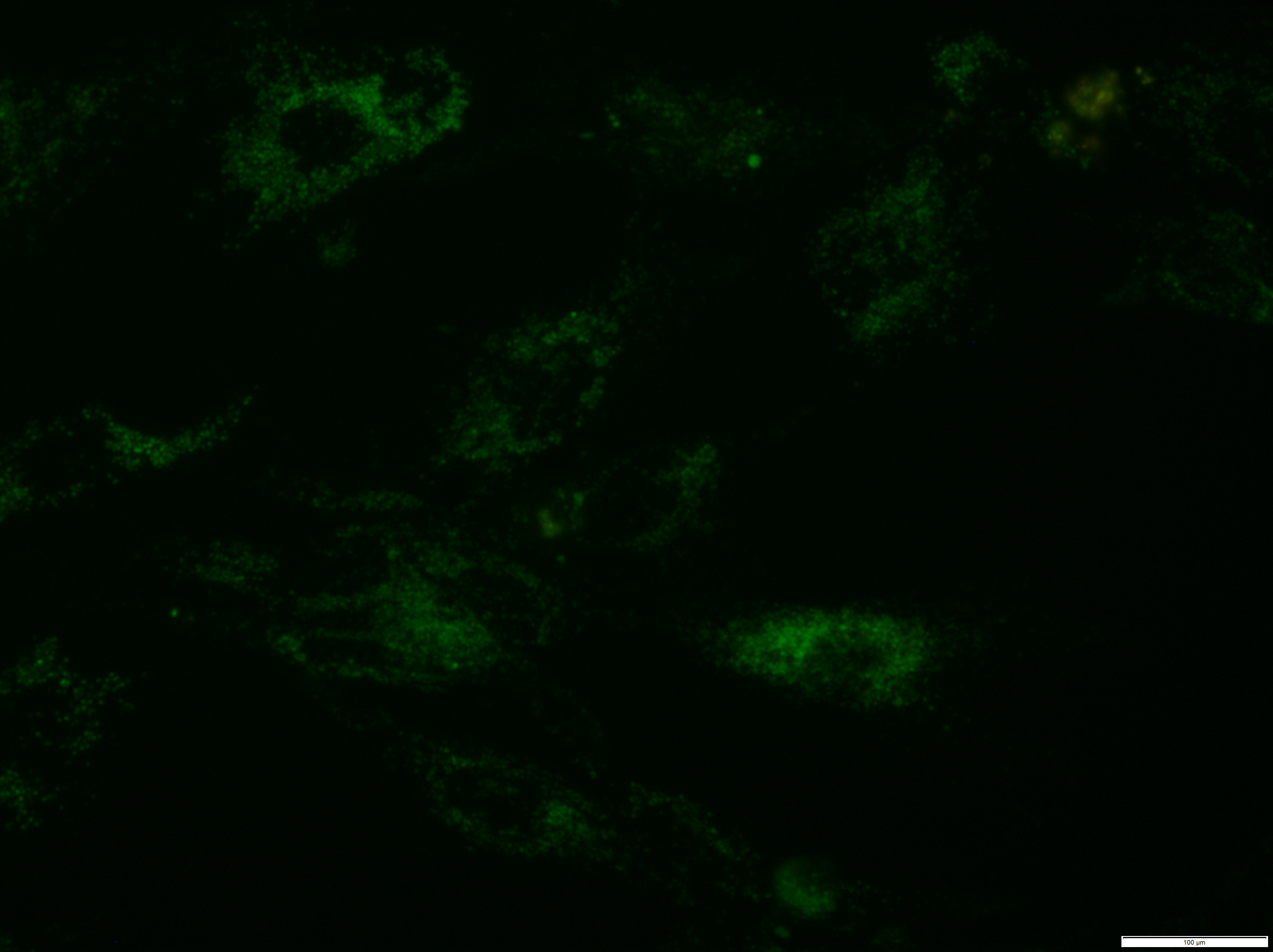

Supplement: Supplementary file 1 — Supplementary Information 1. [file 41598_2024_54722_MOESM1_ESM.zip › raw data/Figure2/Andrographolide.tif]

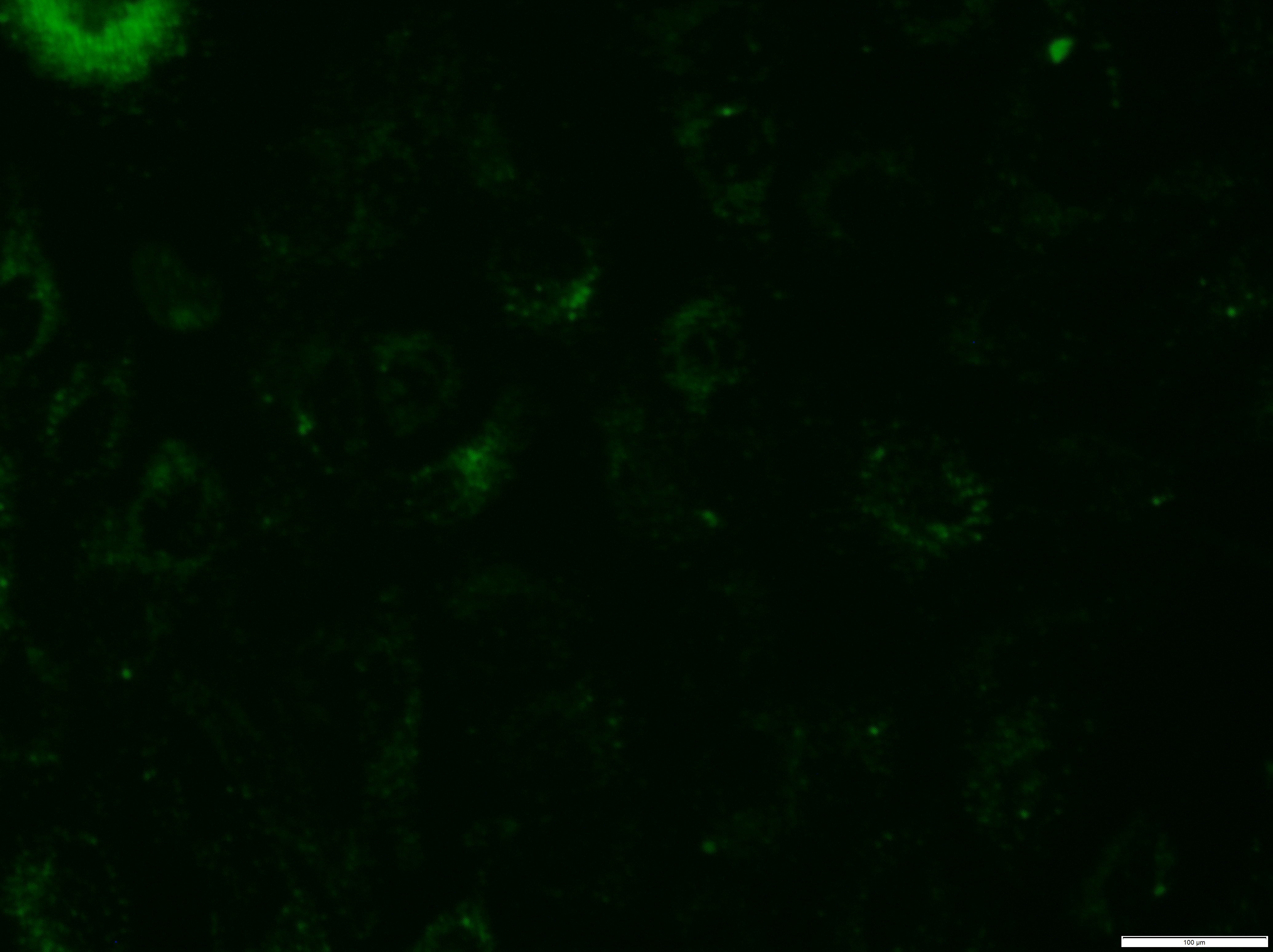

Supplement: Supplementary file 1 — Supplementary Information 1. [file 41598_2024_54722_MOESM1_ESM.zip › raw data/Figure2/Baicalin.tif]

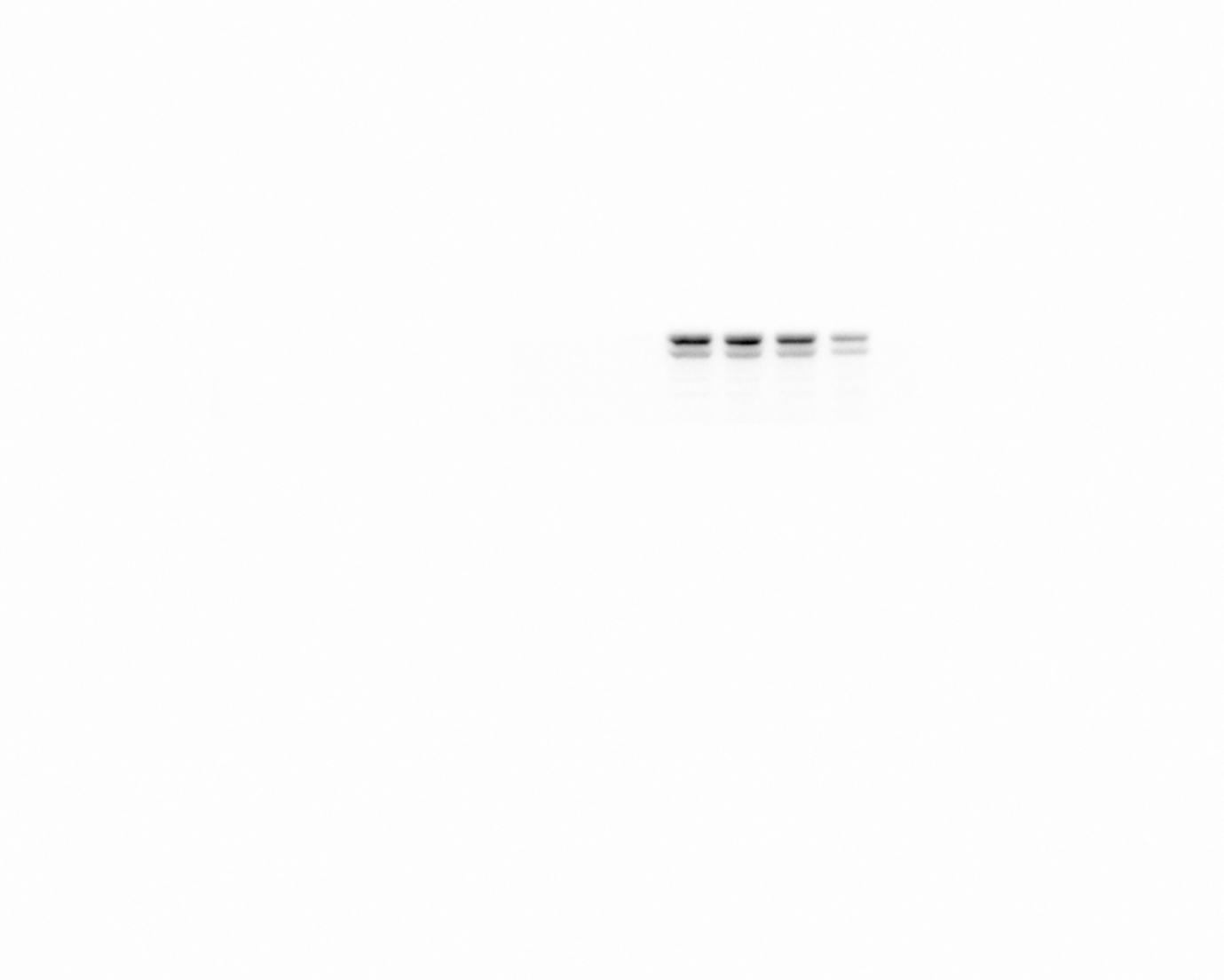

Supplement: Supplementary file 1 — Supplementary Information 1. [file 41598_2024_54722_MOESM1_ESM.zip › raw data/Figure2/Figure2.A/ACE2-24h SARA-COV-2, SARA-COV-2+Andrographolide, SARA-COV-2 +baicalin, SARA-COV-2+Andrographolide and baicalin.jpg]

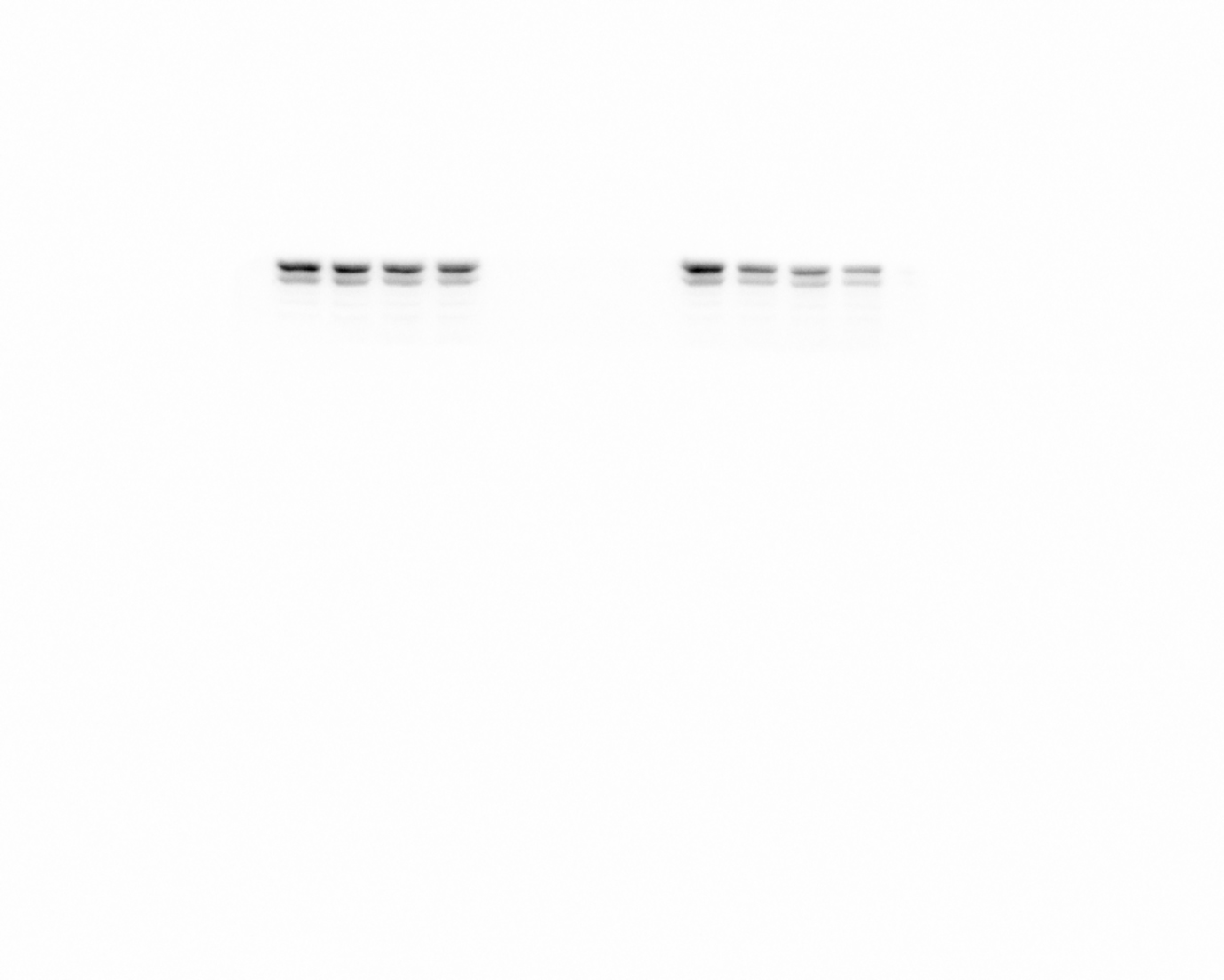

Supplement: Supplementary file 1 — Supplementary Information 1. [file 41598_2024_54722_MOESM1_ESM.zip › raw data/Figure2/Figure2.A/ACE2-3h,ACE2-12h SARA-COV-2, SARA-COV-2+Andrographolide, SARA-COV-2 +baicalin, SARA-COV-2+Andrographolide and baicalin.jpg]

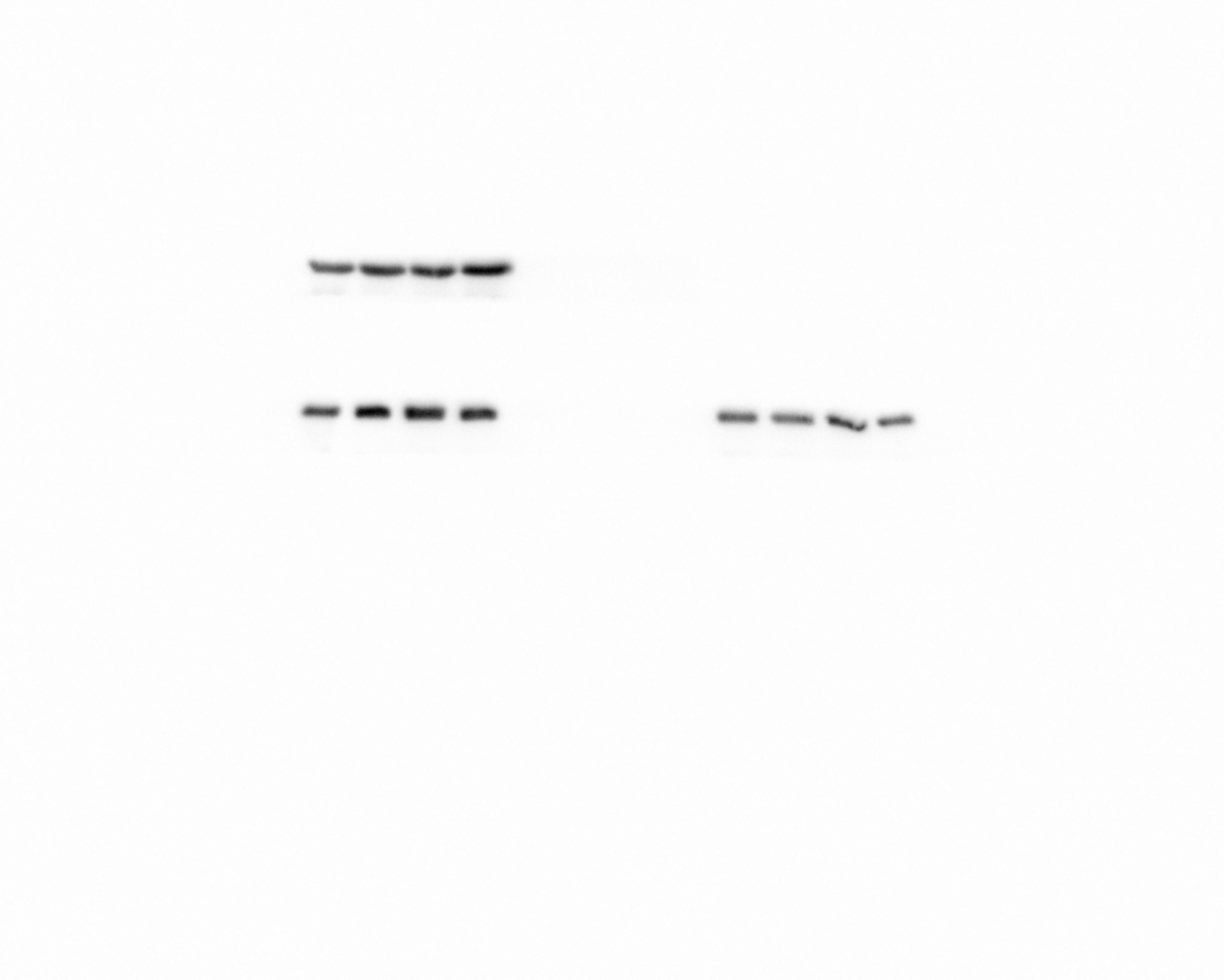

Supplement: Supplementary file 1 — Supplementary Information 1. [file 41598_2024_54722_MOESM1_ESM.zip › raw data/Figure2/Figure2.A/actin-3h,actin-12h,actin-24h SARA-COV-2, SARA-COV-2+Andrographolide, SARA-COV-2 +baicalin, SARA-COV-2+Andrographolide and baicalin.jpg]

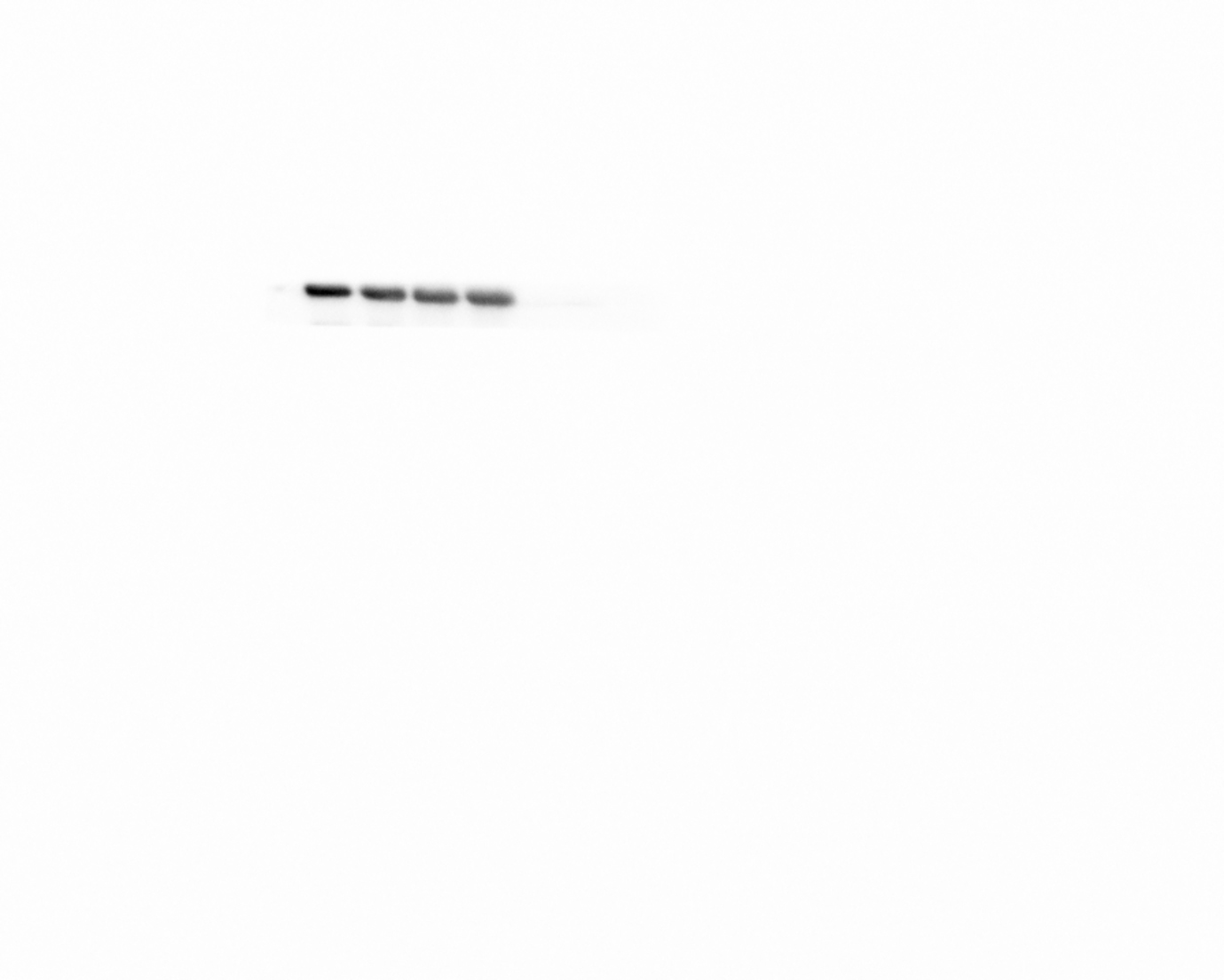

Supplement: Supplementary file 1 — Supplementary Information 1. [file 41598_2024_54722_MOESM1_ESM.zip › raw data/Figure2/Figure2.A/GFP-24h SARA-COV-2, SARA-COV-2+Andrographolide, SARA-COV-2 +baicalin, SARA-COV-2+Andrographolide and baicalin.jpg]

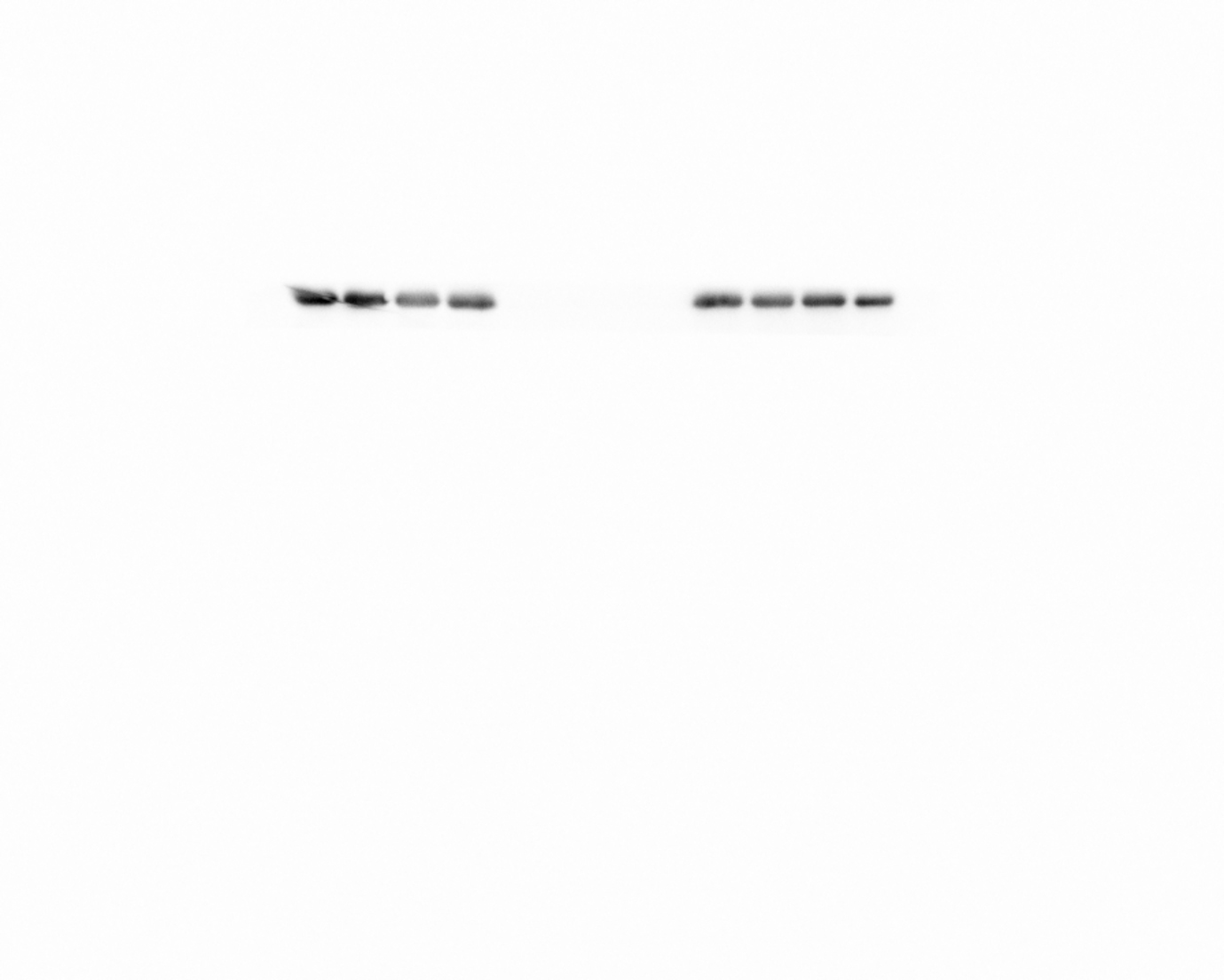

Supplement: Supplementary file 1 — Supplementary Information 1. [file 41598_2024_54722_MOESM1_ESM.zip › raw data/Figure2/Figure2.A/GFP-3h,GFP-12h SARA-COV-2, SARA-COV-2+Andrographolide, SARA-COV-2 +baicalin, SARA-COV-2+Andrographolide and baicalin.jpg]

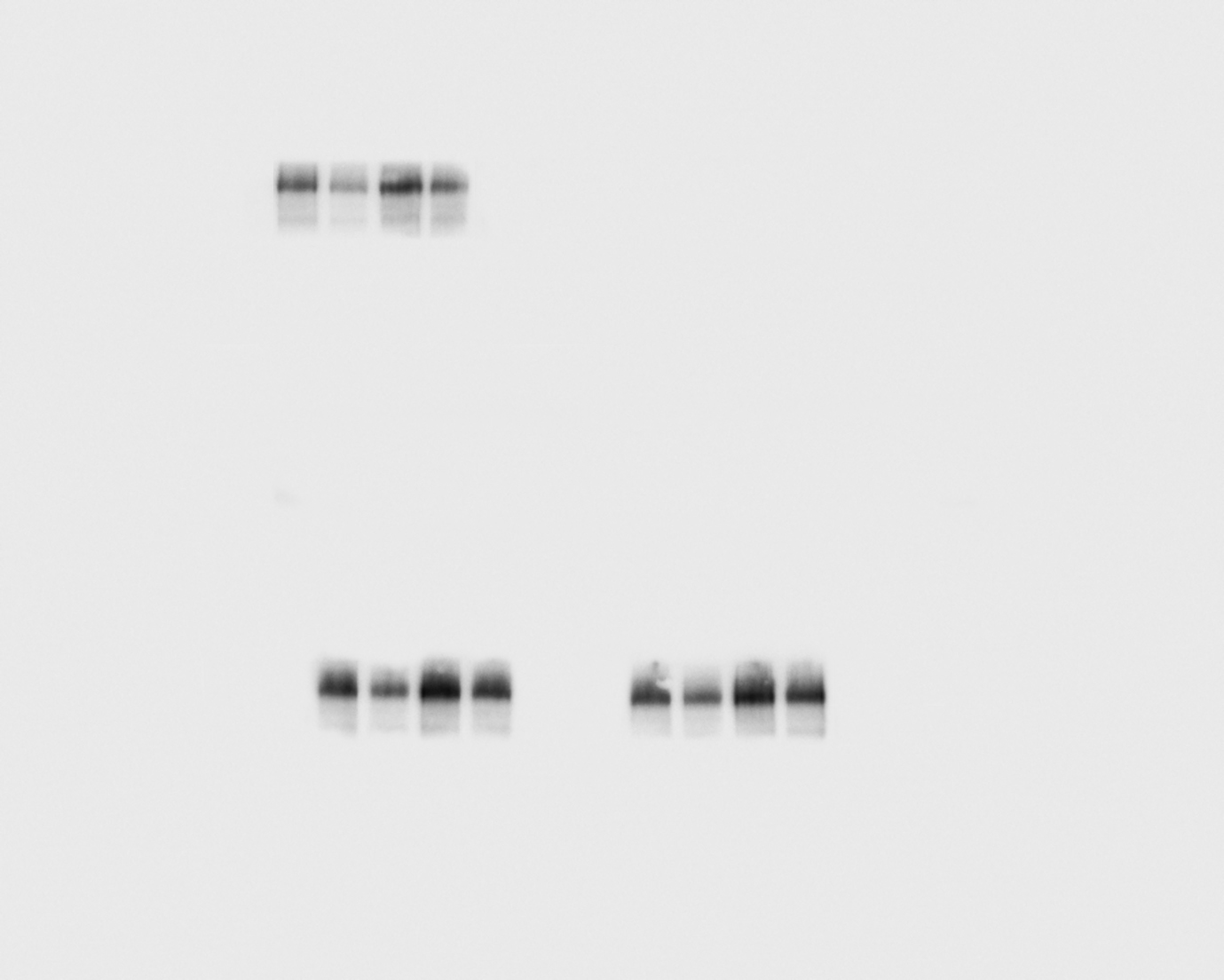

Supplement: Supplementary file 1 — Supplementary Information 1. [file 41598_2024_54722_MOESM1_ESM.zip › raw data/Figure2/Figure2.B/ACE2 ó┘ DMSO(MOCK,A+B) MG132(MOCK;A+B)ú╗ó┌ DMSO(MOCK,A+B) BAfA1(MOCK;A+B)ú╗ó█ DMSO(MOCK,A+B) Ma┬CD(MOCK;A+B).jpg]

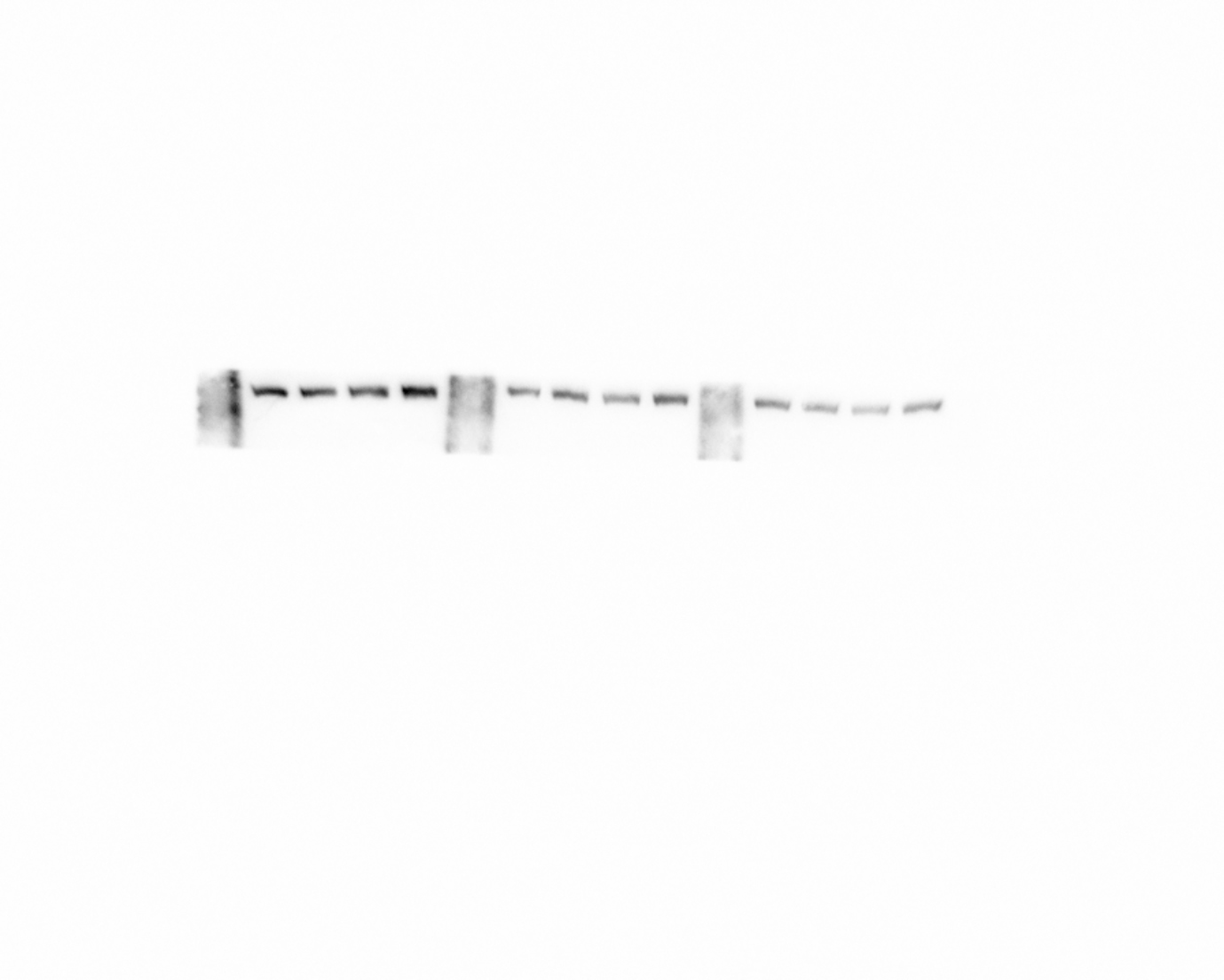

Supplement: Supplementary file 1 — Supplementary Information 1. [file 41598_2024_54722_MOESM1_ESM.zip › raw data/Figure2/Figure2.B/actinó┘ DMSO(MOCK,A+B) MG132(MOCK;A+B)ú╗ó┌ DMSO(MOCK,A+B) BAfA1(MOCK;A+B)ú╗ó█ DMSO(MOCK,A+B) Ma┬CD(MOCK;A+B).jpg]

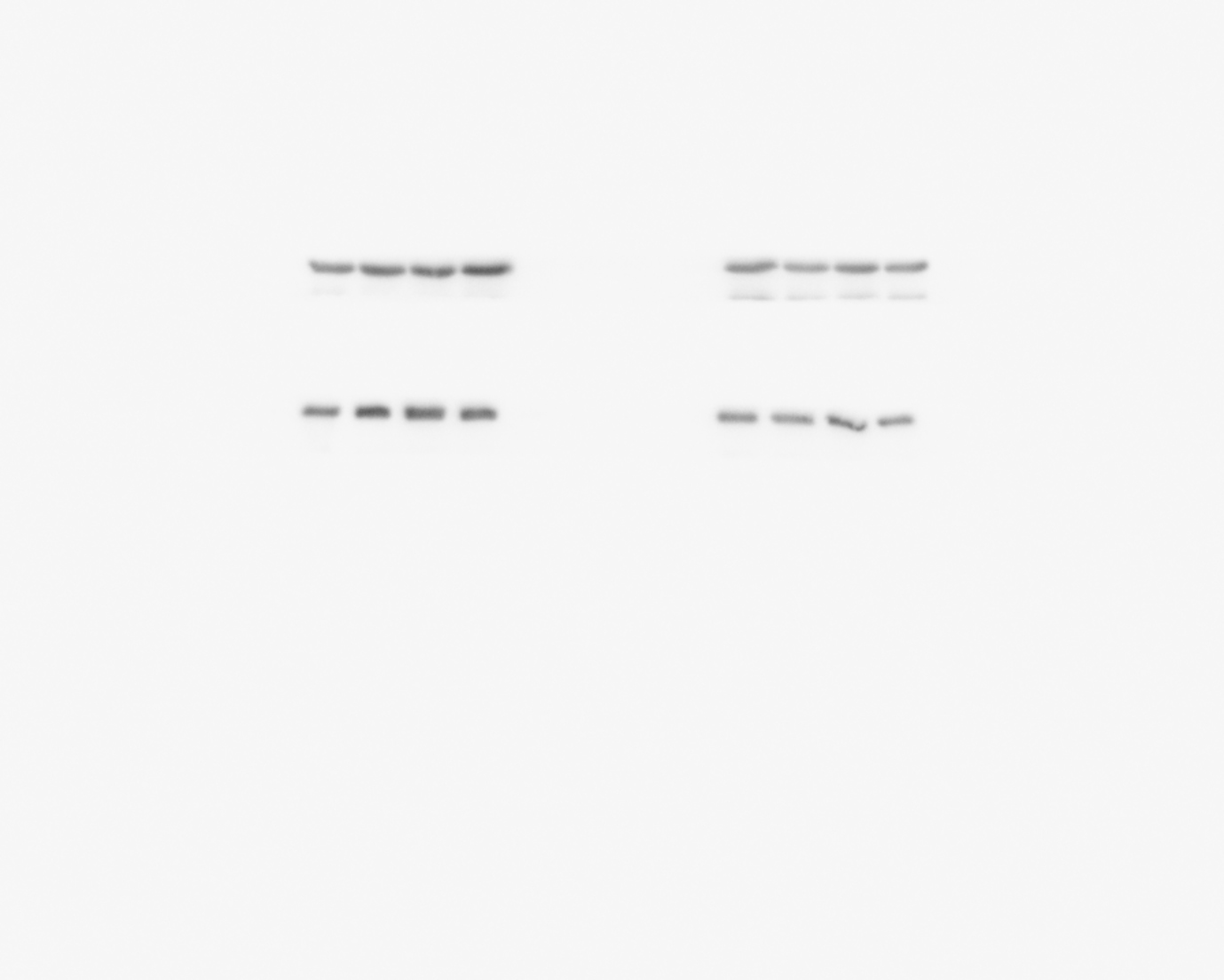

Supplement: Supplementary file 1 — Supplementary Information 1. [file 41598_2024_54722_MOESM1_ESM.zip › raw data/Figure2/Figure2.B/GFPó┘ DMSO(MOCK,A+B) MG132(MOCK;A+B)ú╗ó┌ DMSO(MOCK,A+B) BAfA1(MOCK;A+B)ú╗ó█ DMSO(MOCK,A+B) Ma┬CD(MOCK;A+B);ó▄DMSO(MOCK,A+B) Ma┬CD(MOCK;A+B).jpg]

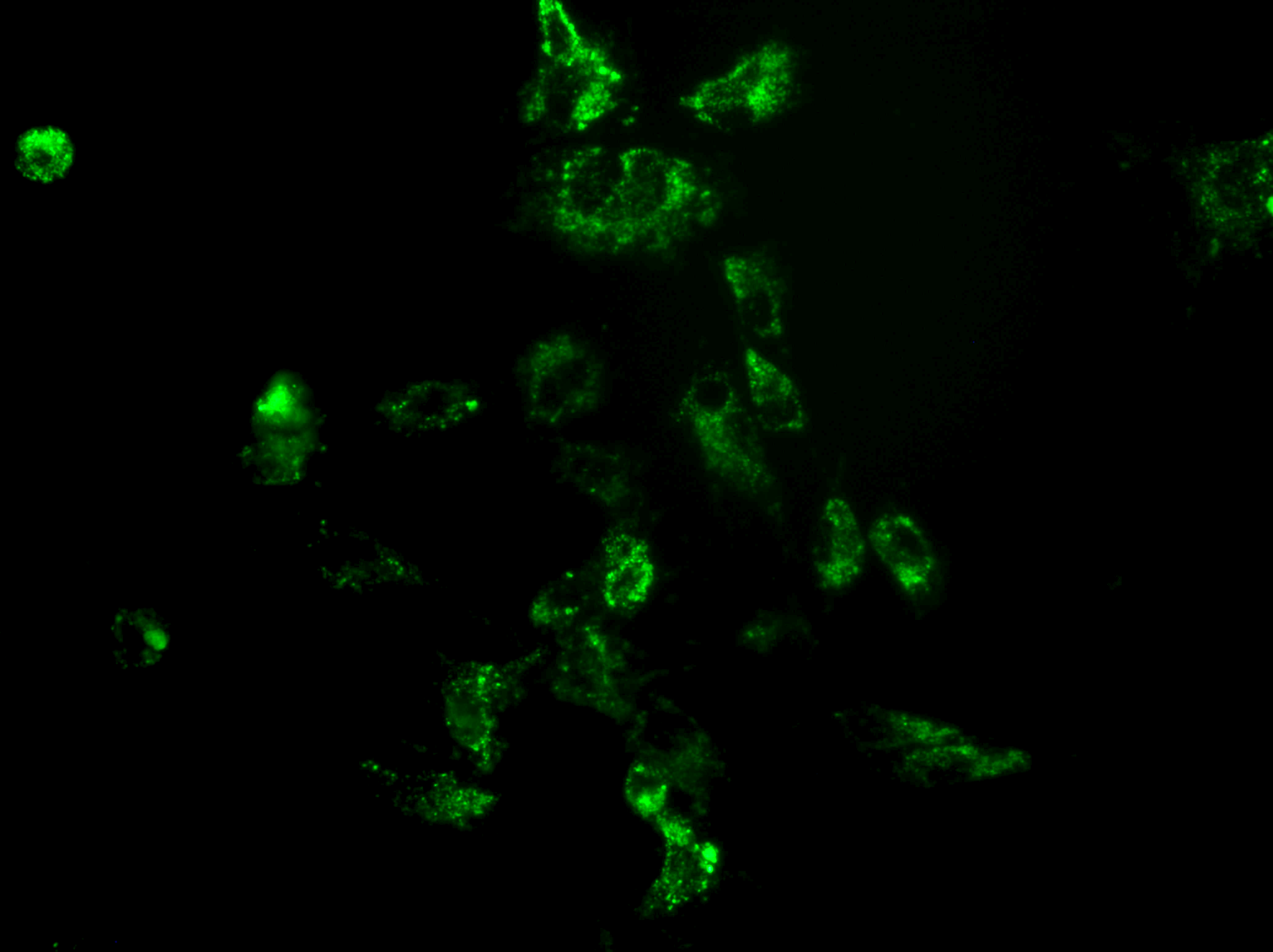

Supplement: Supplementary file 1 — Supplementary Information 1. [file 41598_2024_54722_MOESM1_ESM.zip › raw data/Figure2/MOCK.jpg]

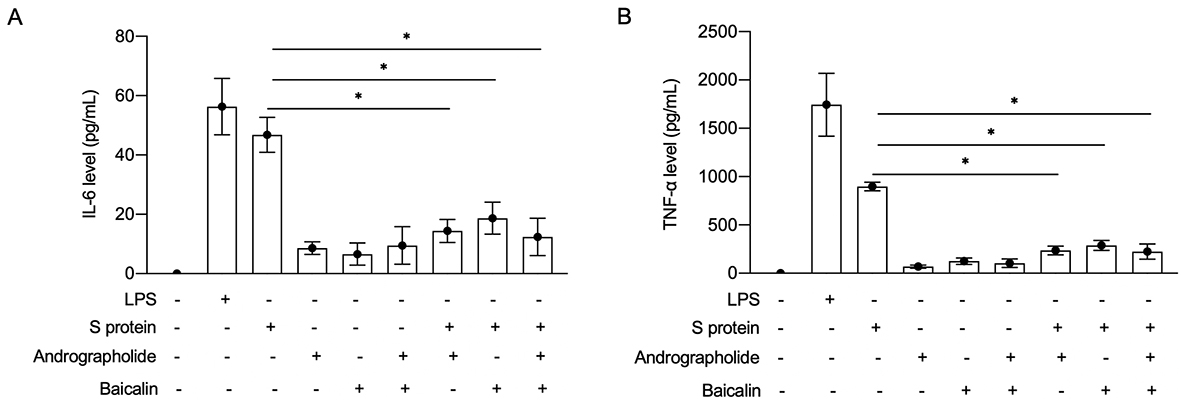

Supplement: Supplementary file 1 — Supplementary Information 1. [file 41598_2024_54722_MOESM1_ESM.zip › raw data/Figure3/Fig.3.jpg]

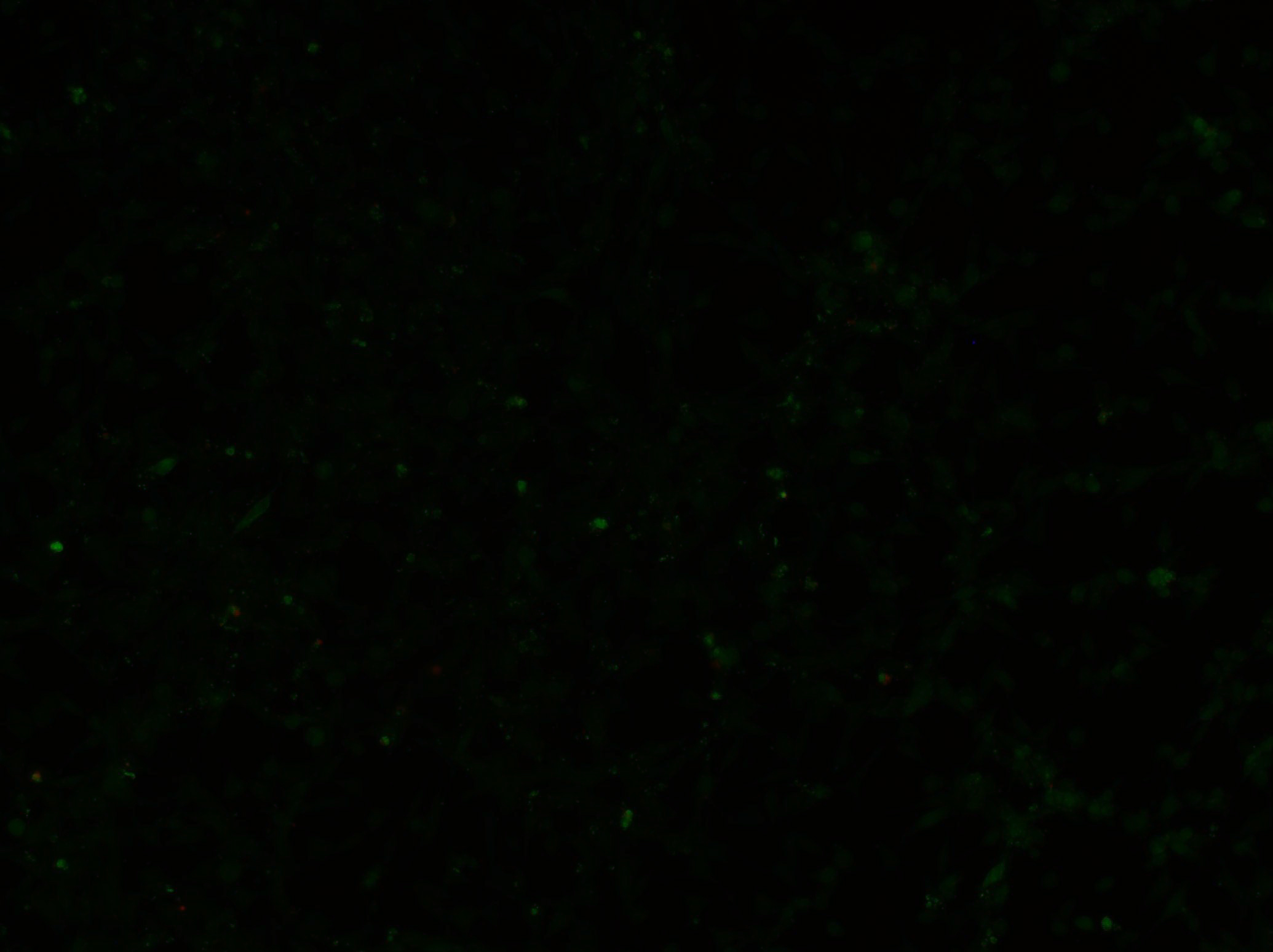

Supplement: Supplementary file 1 — Supplementary Information 1. [file 41598_2024_54722_MOESM1_ESM.zip › raw data/Figure4/Calu-3 Andrographolide.jpg]

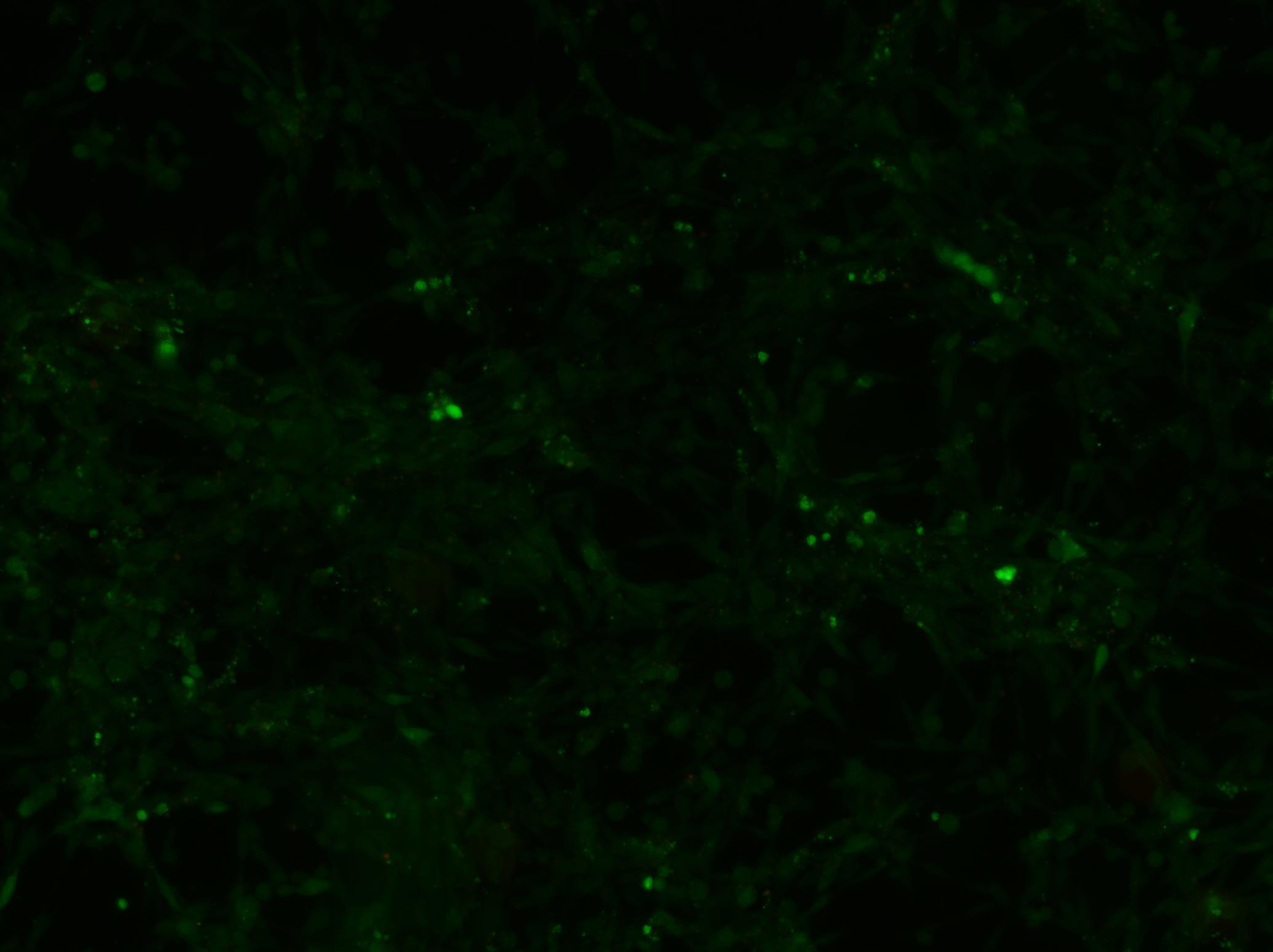

Supplement: Supplementary file 1 — Supplementary Information 1. [file 41598_2024_54722_MOESM1_ESM.zip › raw data/Figure4/Calu-3 Baicalin.jpg]

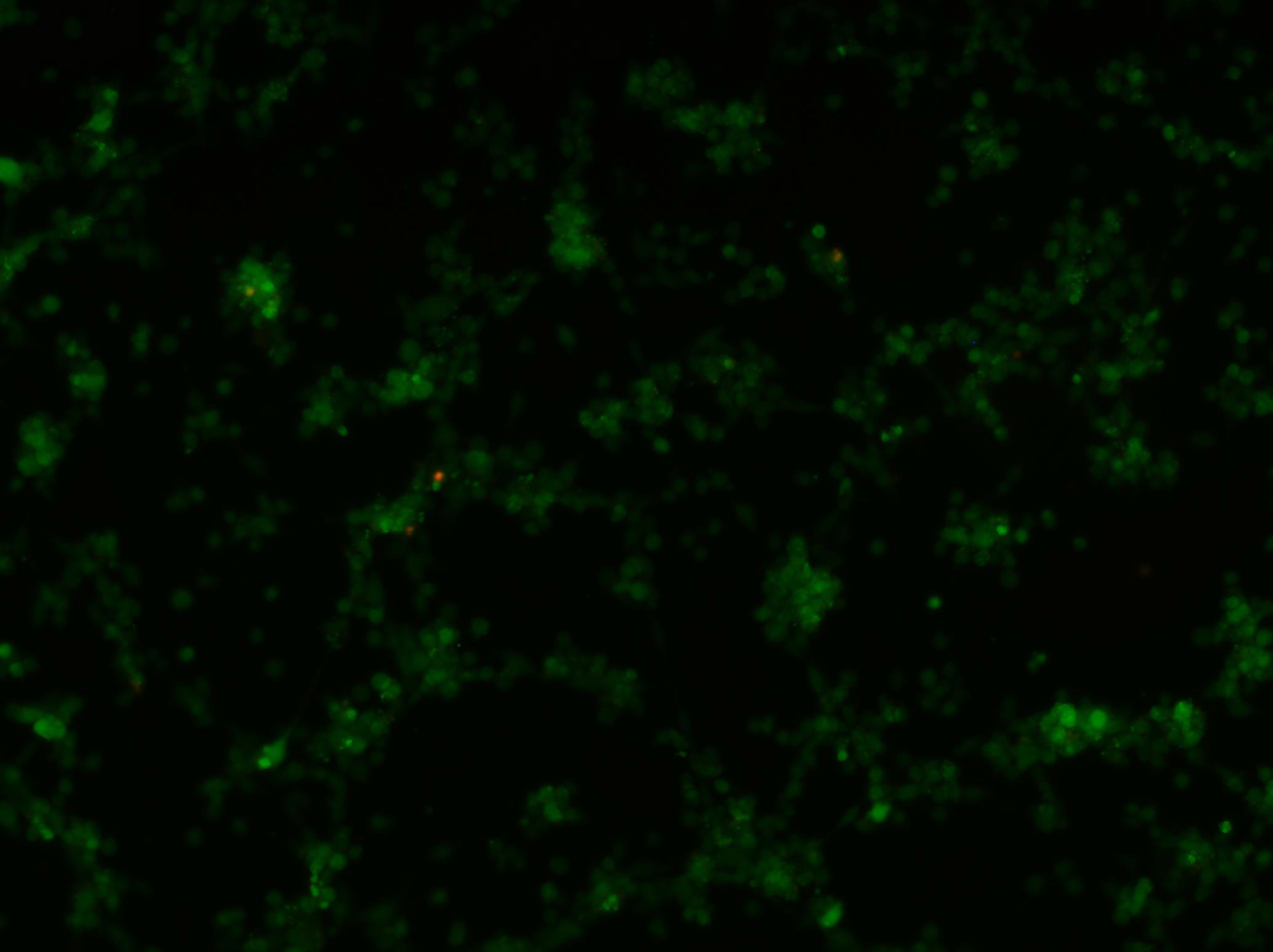

Supplement: Supplementary file 1 — Supplementary Information 1. [file 41598_2024_54722_MOESM1_ESM.zip › raw data/Figure4/Calu-3 MOCK.jpg]

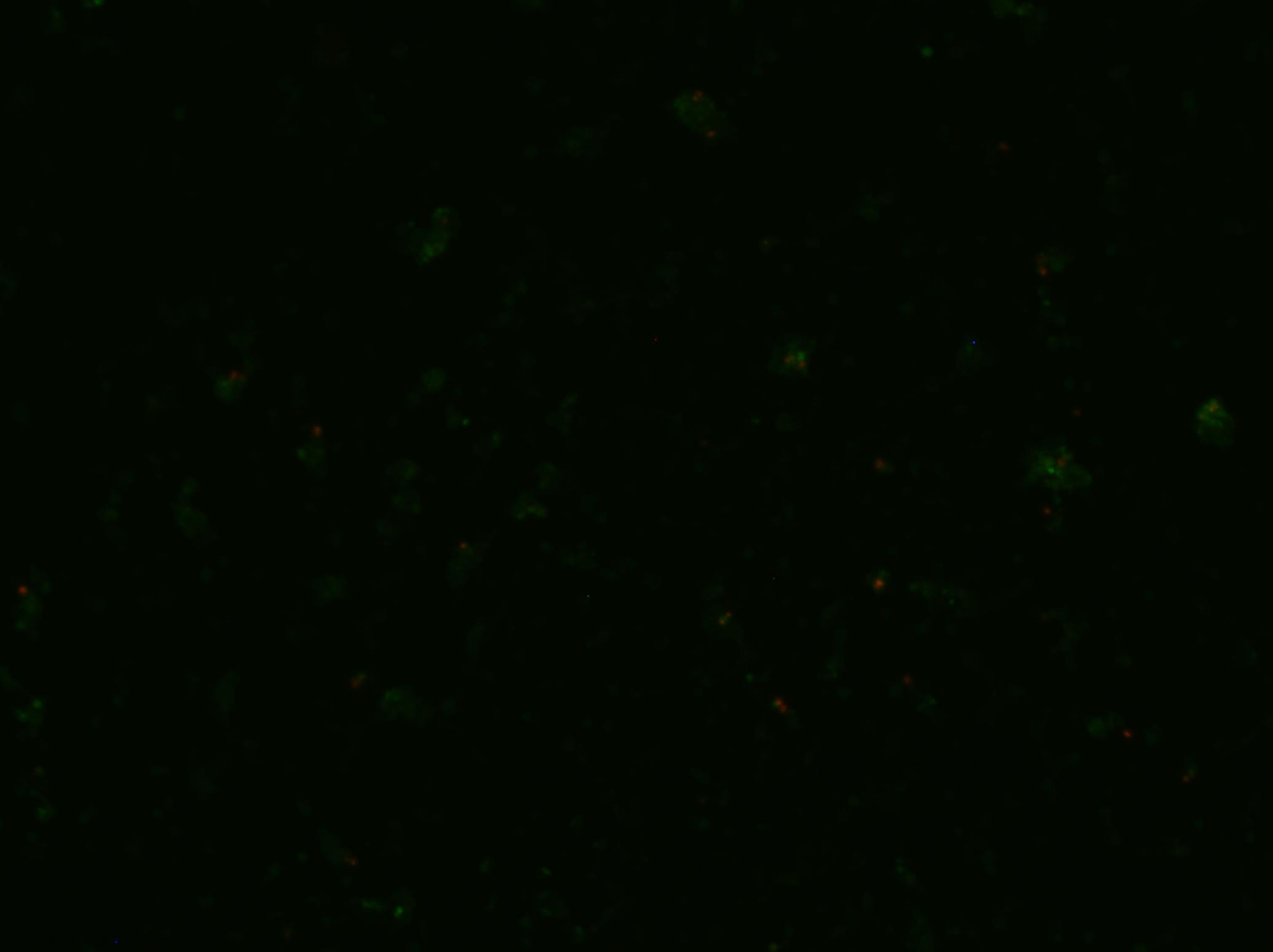

Supplement: Supplementary file 1 — Supplementary Information 1. [file 41598_2024_54722_MOESM1_ESM.zip › raw data/Figure4/HUVECs Andrographolide.jpg]

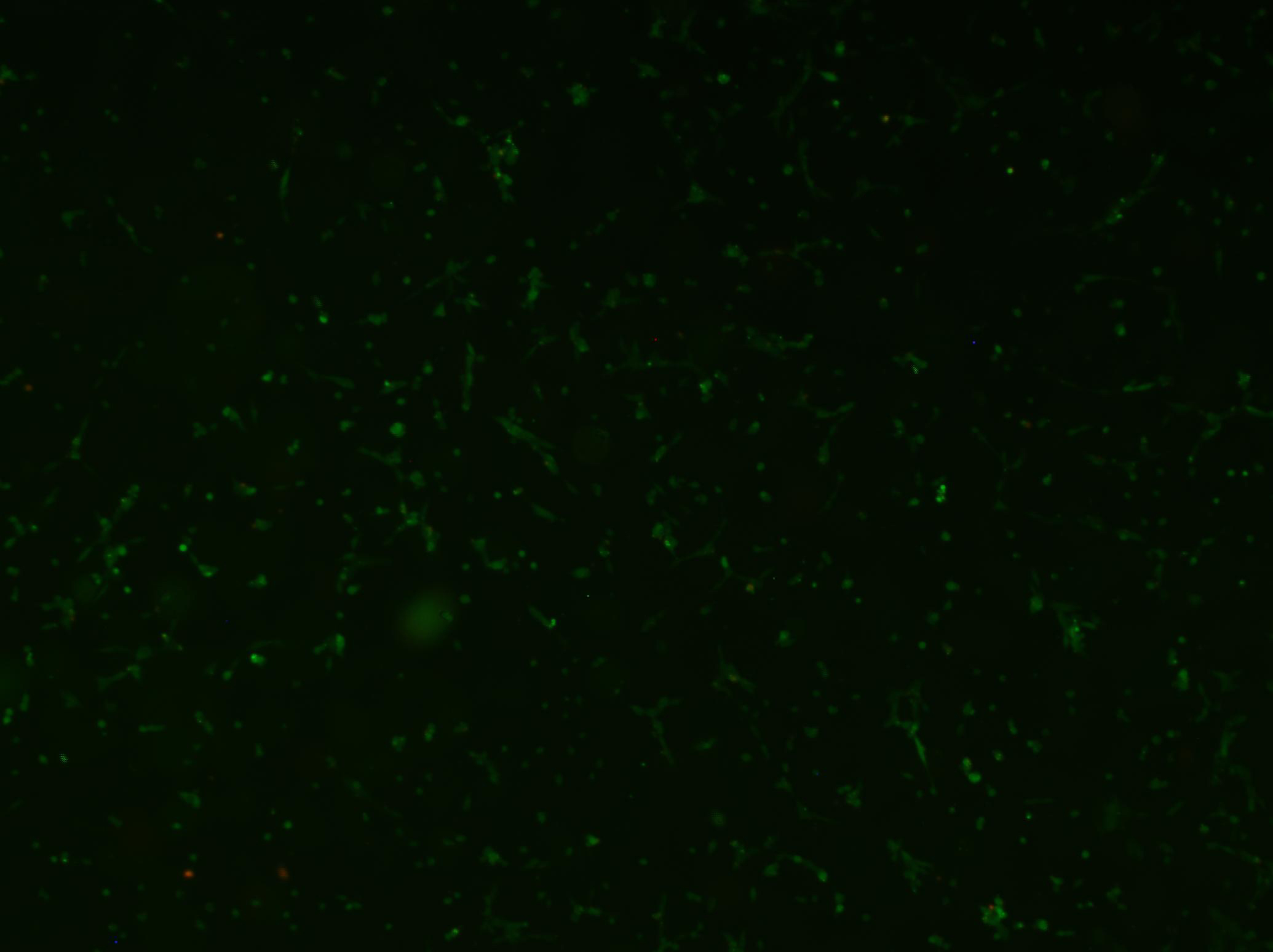

Supplement: Supplementary file 1 — Supplementary Information 1. [file 41598_2024_54722_MOESM1_ESM.zip › raw data/Figure4/HUVECs Baicalin.jpg]

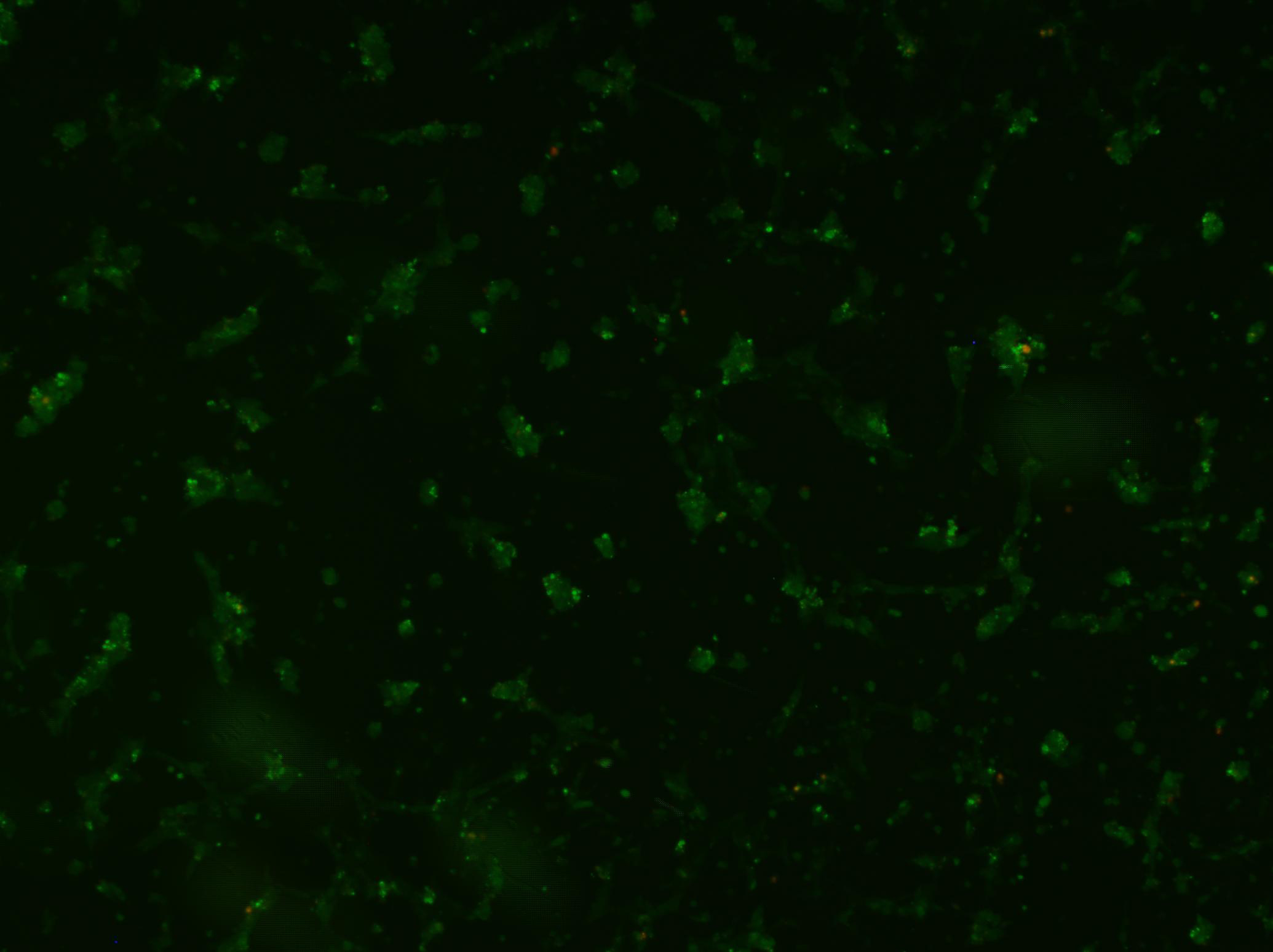

Supplement: Supplementary file 1 — Supplementary Information 1. [file 41598_2024_54722_MOESM1_ESM.zip › raw data/Figure4/HUVECs MOCK.jpg]

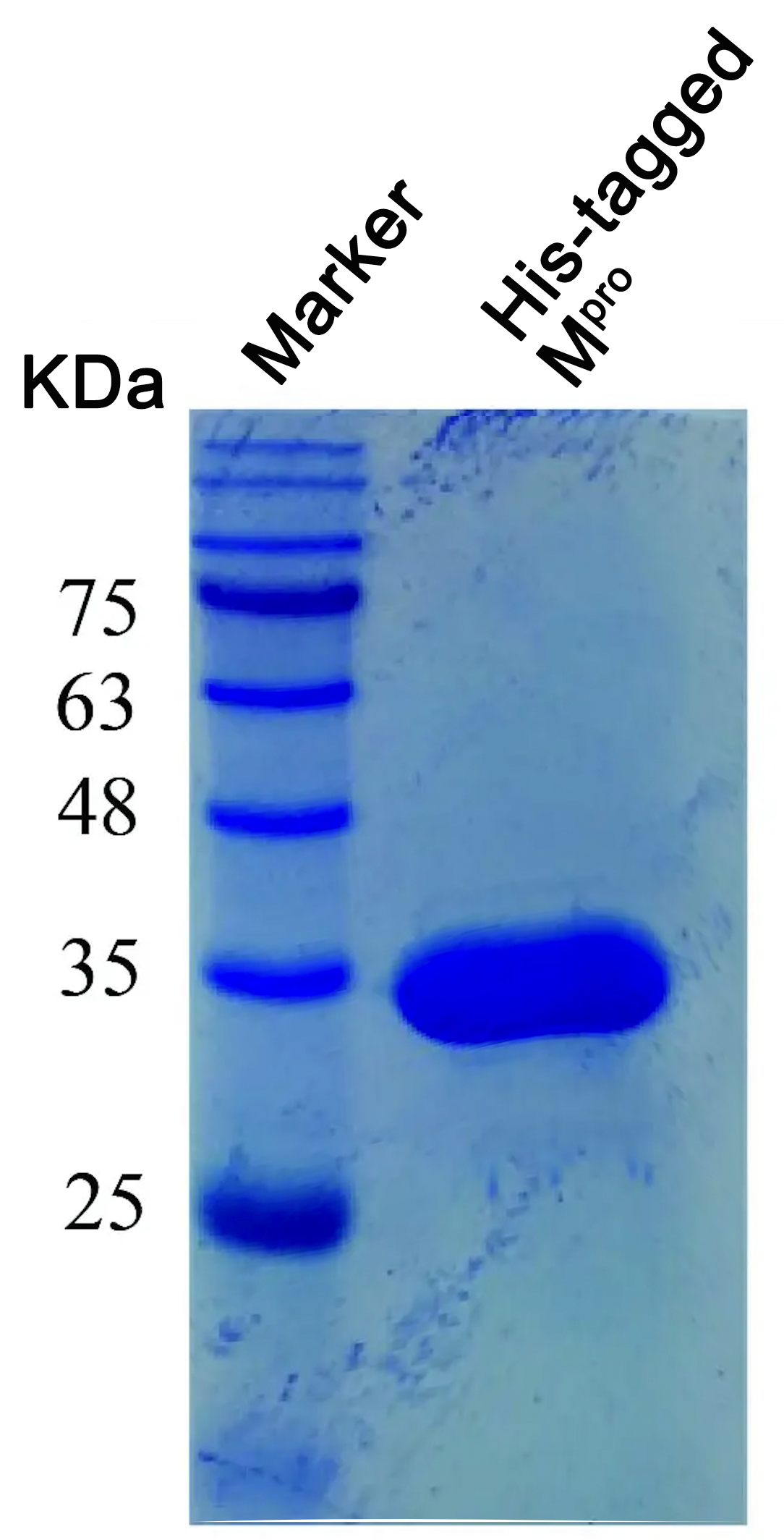

Supplement: Supplementary file 1 — Supplementary Information 1. [file 41598_2024_54722_MOESM1_ESM.zip › raw data/Figure5/Figure5.A Mpro purification.jpg]

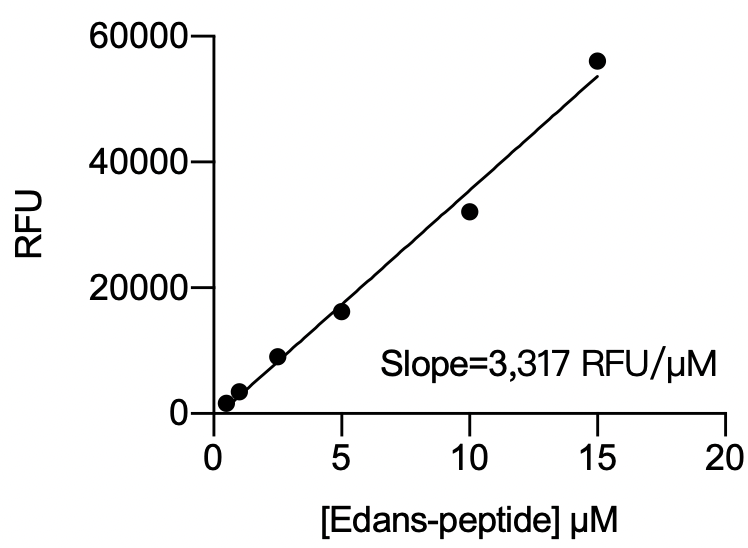

Supplement: Supplementary file 1 — Supplementary Information 1. [file 41598_2024_54722_MOESM1_ESM.zip › raw data/Figure5/Figure5.C RFU, SLOPE.png]

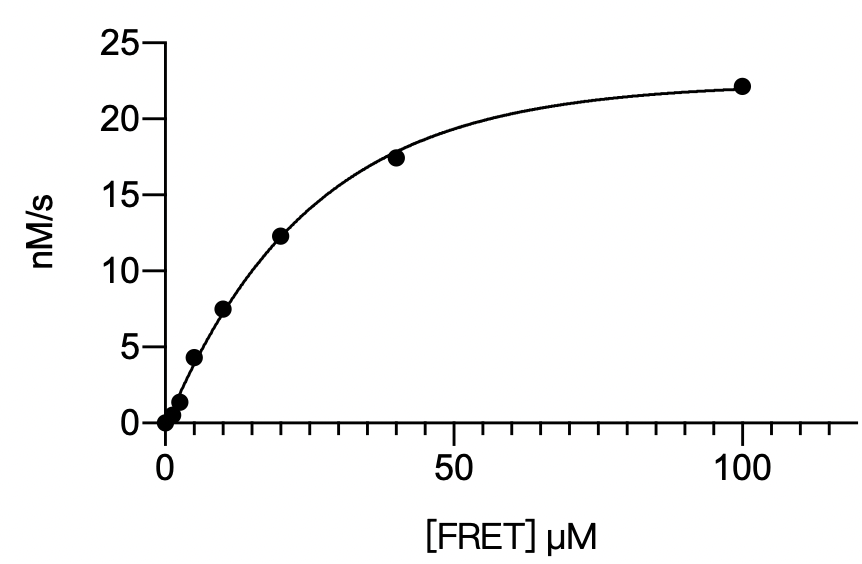

Supplement: Supplementary file 1 — Supplementary Information 1. [file 41598_2024_54722_MOESM1_ESM.zip › raw data/Figure5/Figure5.D FRET assay.png]

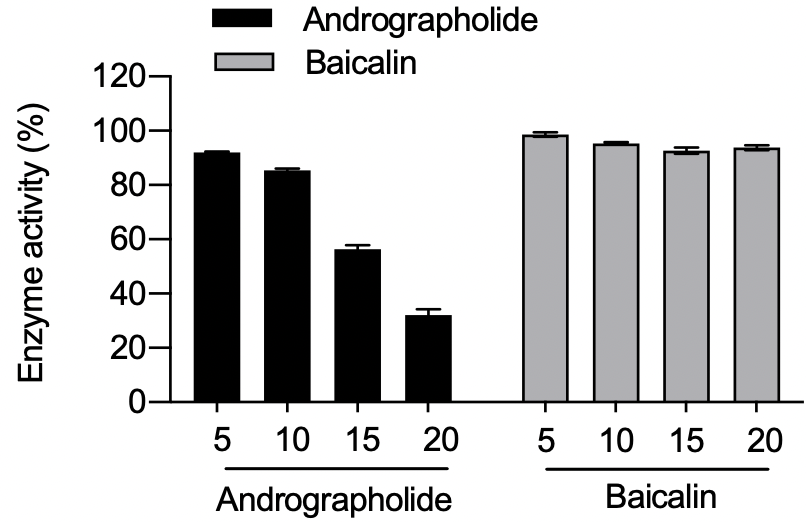

Supplement: Supplementary file 1 — Supplementary Information 1. [file 41598_2024_54722_MOESM1_ESM.zip › raw data/Figure5/Figure5.E Enzyme activity.png]

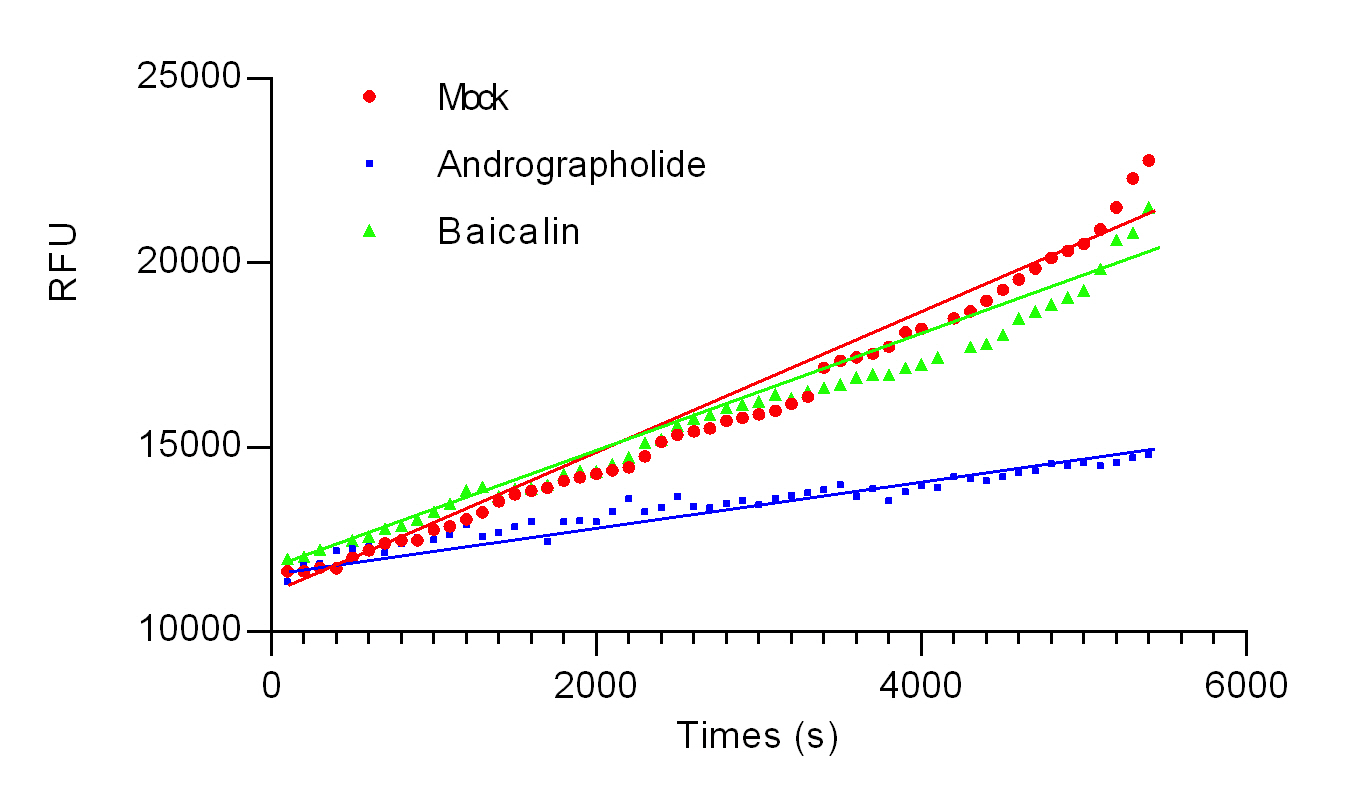

Supplement: Supplementary file 1 — Supplementary Information 1. [file 41598_2024_54722_MOESM1_ESM.zip › raw data/Figure5/Figure5.F.jpg]
