# Supplementary material for: Synergistic inhibition effects of andrographolide and baicalin on coronavirus mechanisms by downregulation of ACE2 protein level
Source: Sci Rep. 2024 Feb 21;14:4287. doi: 10.1038/s41598-024-54722-5 (PMC10882053; doi:10.1038/s41598-024-54722-5)
Supplement: Supplementary file 2 — Supplementary Information 2. [file 41598_2024_54722_MOESM2_ESM.pdf]

STANDARDS OF REPORTING

***In vitro* experiments**
- Name of the cell line, supplier and the number of cells used (in cells/ml or cells/well).
- Cell culture medium (and supplier), any supplementary serum (and supplier), any supplementary antibiotics and their concentrations.
- Culture conditions (temperature and % CO_2 if any).
- Concentration and supplier of drug used.
- Treatment temperature and duration.
- Negative controls must be described.

Animal studies
- Statement of ethics approval, including name of ethics committee.
- The name, the route of administration and the dose of anaesthetic used, if any.
- The method used for euthanasia.
- The housing conditions: Temperature, humidity, light/dark cycle and access to food and water.
- The animals: Strain, total number used, sex, age, average weight at the start of the experiment.

Clinical studies
- Statement of ethics approval, including name of ethics committee.
- Statement of patient consent (written or oral).
- Age and sex distribution of the participants.
- Place and time (month and year) of sample collection.
- Inclusion and exclusion criteria.

Statistical analysis
- Presentation of data (e.g. mean ± standard deviation).
- Number of experimental repeats.
- Name of statistical tests used, including any post hoc tests.
- Name, version and manufacturer of statistical software used.
- Threshold used to determine significance (e.g. $P<0.05$).
- Please note that improper use of statistical tests will delay the publication of the manuscript.

Photomicrographs
- Scale bar and representative length.
- OR magnification stated in the figure legends.

Western blot analysis
- Protein extraction buffer (name and supplier OR components and concentrations).
- Protein concentration determination method.
- Mass of protein loaded per lane and percentage of the resolving/separating gel.
- Type of membrane the proteins were transferred to.
- Blocking reagent and percentage, temperature and duration.
- Primary antibodies: Dilution used, catalogue number, supplier, and temperature and duration of incubations (antibody for the reference protein must been included).
- Secondary antibodies: Dilution, catalogue number, supplier, conjugate, and temperature and duration of incubations.
- Visualisation reagent and supplier.
- Name, version number and supplier of the software used for densitometry.
- Representative western blot images, including all loading controls, are required.

RT-qPCR

- Source of RNA (cells/tissue).
- RNA extraction buffer and supplier.
- RT kit and supplier OR the reverse transcriptase, buffer, dNTPs and primers.
- Temperature and duration of RT.
- qPCR reagent name and supplier.
- Sequences of forward and reverse primers.
- Reference gene should be stated and respective primer sequences provided.
- Thermocycling conditions.
- Method of quantification stated and referenced.

Immunohistochemistry/Immunocytochemistry

- Samples for immunohistochemistry: Whether tissues were paraffin-embedded or snap-frozen, thickness of sections.
- Samples for immunocytochemistry: Name or source of the cells used.
- Blocking reagent and percentage, temperature and duration.
- If serum was used, please state the supplier.
- Primary antibody dilution, catalogue number, supplier, and temperature and duration of incubation.
- Secondary antibody dilution, catalogue number, supplier, conjugate, and temperature and duration of incubation.
- Type of microscope used (representative images are required).

Histology/Staining (all types)

- Fixative used, including concentration, temperature and duration.
- Thickness of sections.
- Stain used, including temperature and duration.
- Type of microscope used (representative images are required).

Flow cytometry

- Sample description and details of any treatments used.
- Description of the analytes and their fluorochromes: Name of the antigen, name of the fluorochrome, catalogue number, supplier.
- Model and supplier of the instrument used to acquire the data.
- Name, version number and supplier of the software used to analyse the data.
- Description of the staining controls.
- Representative flow cytometry histograms/dotplots are required:
    - Histograms/dotplots with poor compensation and/or inconsistent gating are not suitable for publication.
    - The frequencies of the populations within each gate and/or quadrant must be shown.

TUNEL assay

- Fixative used, including concentration, temperature and duration.
- TUNEL reagent, temperature and duration.
- Nuclear stain used, including staining concentration, temperature and duration.
- Mounting medium used.
- Number of fields of view observed by microscopy (representative images are required).

Wound healing assay

- Cell line(s), and concentration(s) and supplier(s) of any drugs used.
- Cells should be serum-starved during the wound healing assay. If serum was required to maintain cell viability, a reference from the literature should be provided.

- Width of the wound being measured should be shown in the figure using lines at the edge of the wound or a line across the wound.
- Type of microscope (images from the 0 h and the ending timepoints are required).

Transwell (migration) and Matrigel (invasion) assays

- Transwell inserts used.
- For invasion assays only: Matrigel details (including temperature and duration of precoating).
- Cell number and medium plated in upper chamber and any drug treatments.
- Medium and serum (type and concentration) plated in lower chamber.
- Incubation time and temperature.
- Stain used, including temperature and duration.
- Type of microscope used (representative images are required).

Transfection

- For overexpression plasmids: The name of the gene of interest, plasmid backbone and supplier
- For shRNA-containing plasmids: The sequence of the shRNA, plasmid backbone and supplier (including those of the negative controls)
- For siRNA and microRNA: The sequence and supplier (including those of the negative controls)
- Mass/concentration of nucleic acid used to transfect cells.
- Name of transfection reagent and supplier.
- Duration and temperature of incubation with transfection reagent.
- Time interval between transfection and subsequent experimentation.
- Proof of successful transfection is required:
    - It must be demonstrated that the expression of the gene or protein of interest is significantly reduced compared with an appropriate negative control (usually a non-targeting sequence for siRNA, shRNA and microRNA, or an empty plasmid for plasmid transfections).
    - Proof of transfection using fluorescence (GFP, RFP etc.), or using antibodies against tagged proteins (V5 epitope, 6x His tag, etc.) is also acceptable.

Transduction

- Name and supplier of vectors used and generation (2nd or 3rd).
- Mass or concentration of each vector used for transfection.
- Name and supplier of the packaging cell line used to generate the virions.
- Method used to transfect the packaging cell line, including name and supplier of any transfection reagent used, and the temperature and duration of incubation.
- Method used to harvest viral supernatant.
- Method used for transduction of target cells, and the duration and temperature of incubation.
- Name of the target cells transduced with the harvested virions and the MOI.
- Details of screening method.
- Proof of successful transduction is required.

Dual luciferase reporter assay

- Name of the genes from which the 3' UTRs have been taken.
- Name, sequences and manufacturer of miRNA mimic/inhibitor and miR-NCs.
- Name and manufacturer of luciferase reporter plasmid.
- Transfection reagent/kit name and manufacturer.
- Duration between transfection and activity measurement.
- Name and manufacturer of the kit used for activity measurement.

Fluorescence *in situ* hybridization (FISH)

- Sample (tissue/cell culture/chromosome) preparation, including fixing.
- Reagents used washes, hybridization, blocking, counterstain, if applicable.

- Name, manufacturer, catalogue number, conjugate and dilution of primary and secondary antibodies, as well as temperature/duration of incubation
- Method of detection, including type of microscope, wavelength, magnification.
- Software used for analysis, if applicable.
- Probes:
  - If a kit was used and/or probes were purchased: Name of kit/probes and manufacturer.
  - If probes were made in the lab: Details of probe synthesis.
    - Type of probe used, source of template and manufacturer, if applicable.
    - Probe length and sequence.
    - Probe linearization, if applicable.
    - RNA polymerase used and manufacturer.
    - RNase inhibitor used and manufacturer.
    - Type of labelling used/labelled nucleotide.
    - Reverse transcription (name of kit used), if applicable.
    - Primer sequences and PCR thermocycling conditions, if applicable.
    - DNase treatment, including manufacturer; temperature and duration.

Xenograft experiments

- Name of cell line injected and number of cells injected per site.
- Medium/vehicle used to resuspend the cells to inject.
- Site of injection/implantation.
- Time interval between injection final tumour growth measurement and/or end of experiment.
- Representative images of the excised tumours with ruler or scale bar are required.
- The general requirements of the “Animal studies” section also apply.

Sanger sequencing

- Representative Sanger sequencing traces (chromatograms) are required.

High-throughput sequencing

If sequencing was performed by a company, the company should be named, and the information below still needs to be provided.

- Name, catalogue number and supplier of the kit used to prepare DNA/RNA samples for sequencing.
- Method used to verify the quality/integrity of the processed samples.
- Type of sequencing done, including nucleotide length and the direction of sequencing (e.g. 75 bp paired end).
- Name, catalogue number and supplier of the sequencing kit.
- Loading concentration (in moles) of the final library, including how concentrations were measured.
- The data must be submitted to a freely available curated database.

Bioinformatics

- Name of all software used, along with a reference or URL.
- Output (for example, ‘differentially expressed genes were defined as…’) and parameters analysed (for example, FDR < XXX, $\log_2$ fold change > XXX or adjusted P-value < XXX).
- For datasets downloaded from public databases: Accession number of the dataset.

Microarray

- Source of the samples (names of the cell lines used, or tissue type)
- The commercial name and manufacturer of the RNA extraction kit, reverse transcription kit and microarray chip.
- The name of the instrument used to scan the microarray
- The data must be submitted to a freely available curated database.
