# Supplementary material for: Synergistic inhibition effects of andrographolide and baicalin on coronavirus mechanisms by downregulation of ACE2 protein level
Source: Sci Rep. 2024 Feb 21;14:4287. doi: 10.1038/s41598-024-54722-5 (PMC10882053; doi:10.1038/s41598-024-54722-5)

Western blot multiple exposure images

Fig3A.Western blot raw image


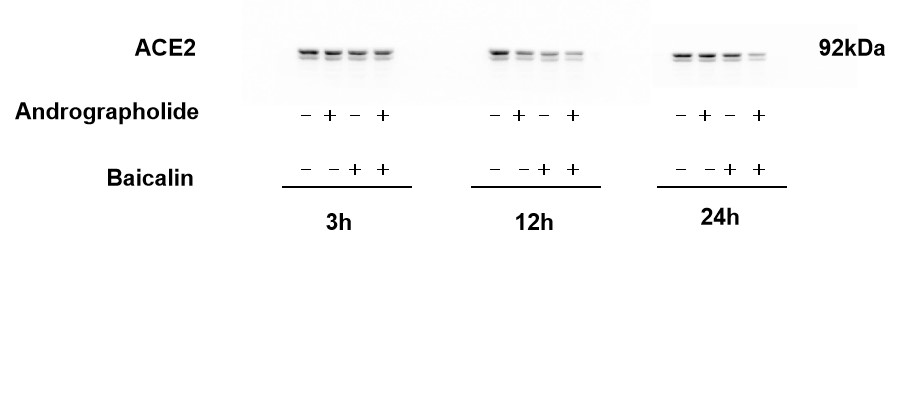


Multiple exposed images:

①ACE2：


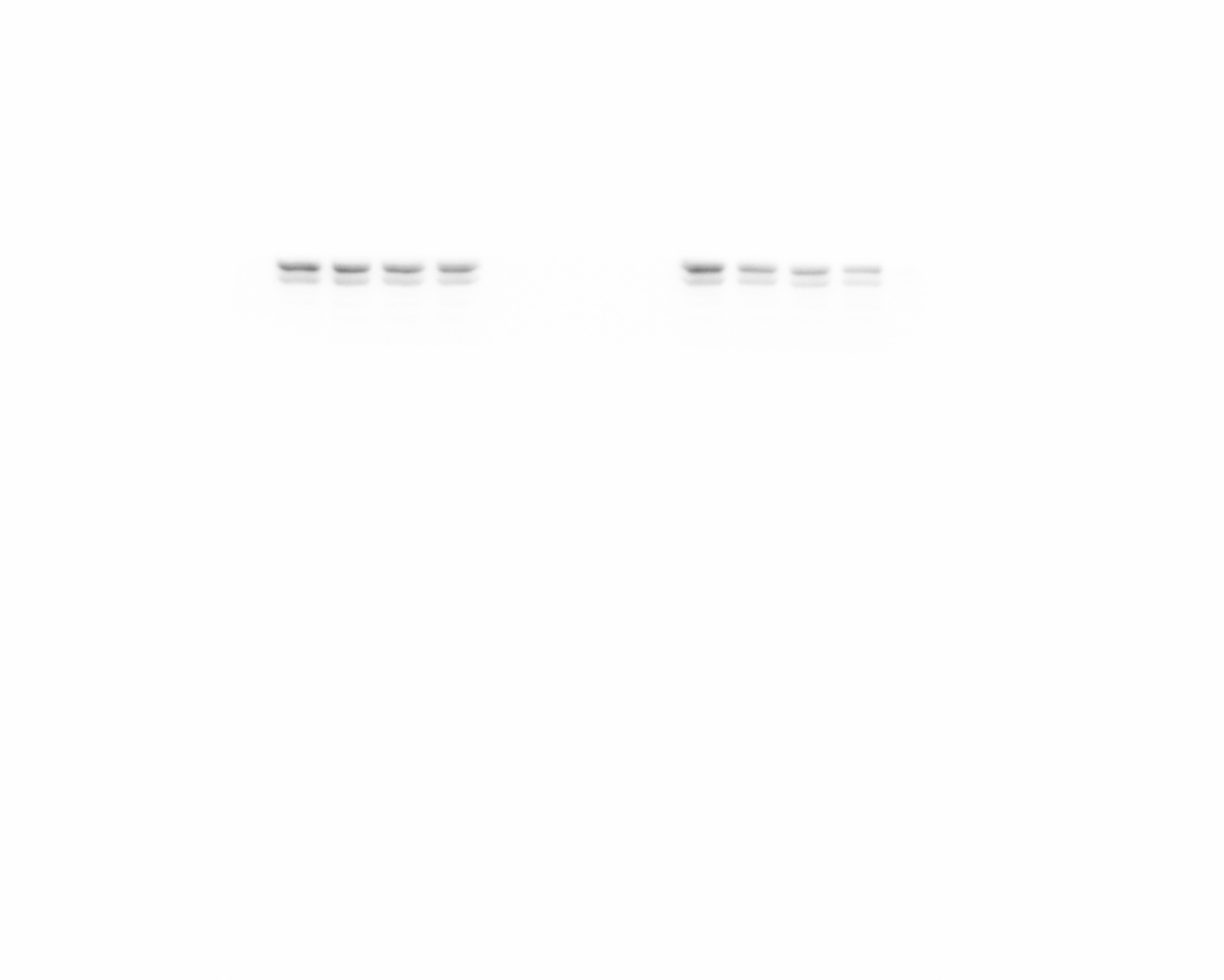


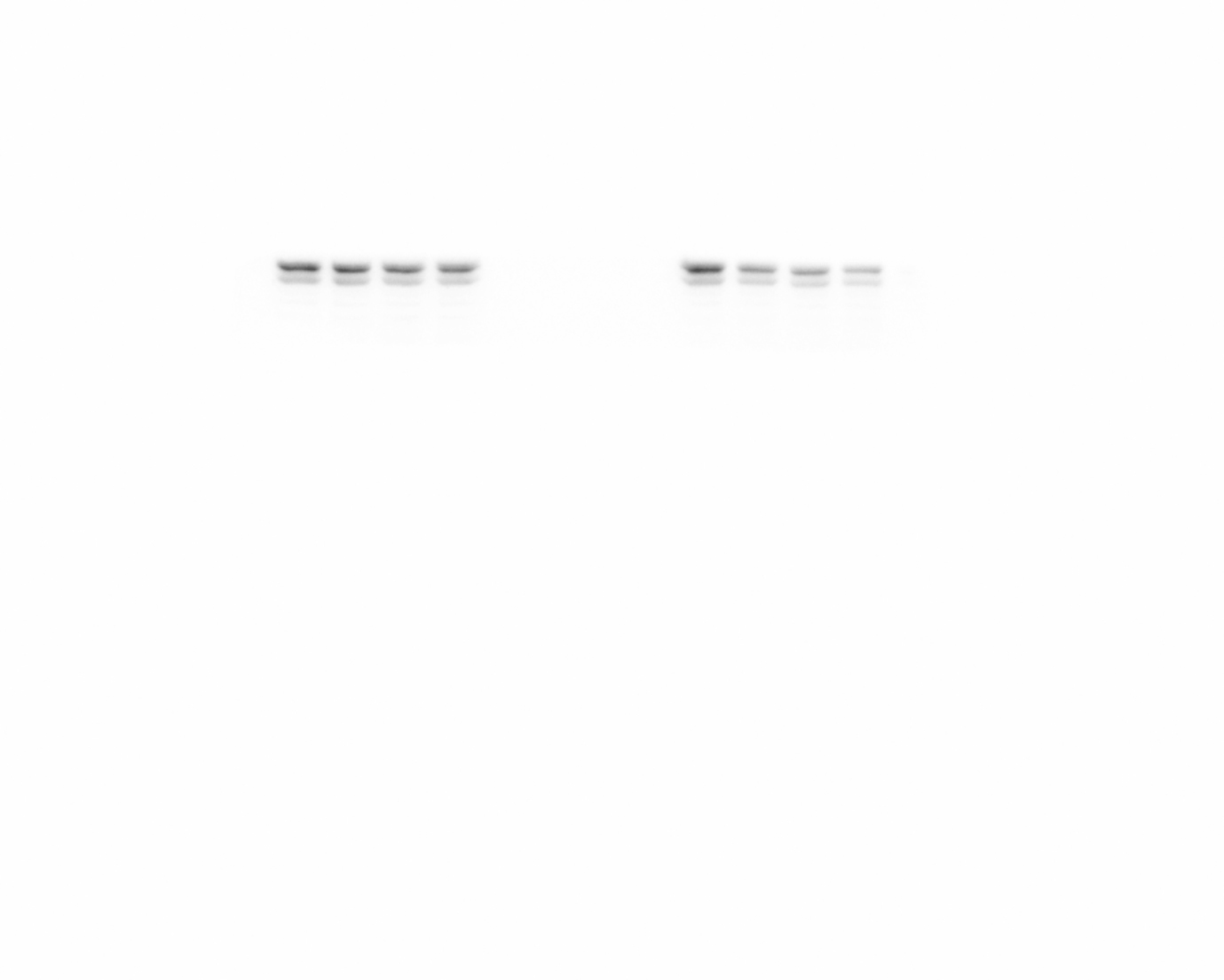


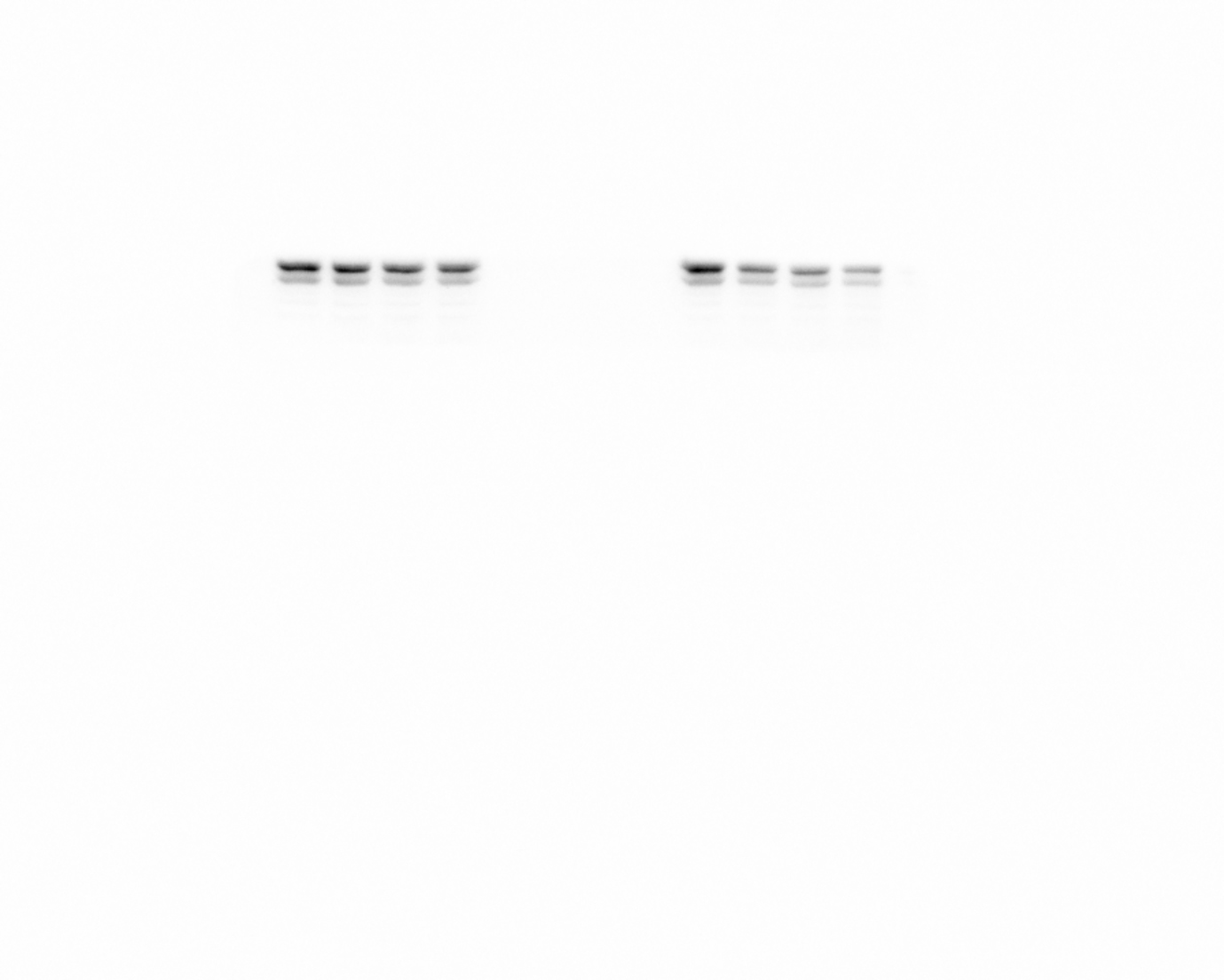


②ACE2：


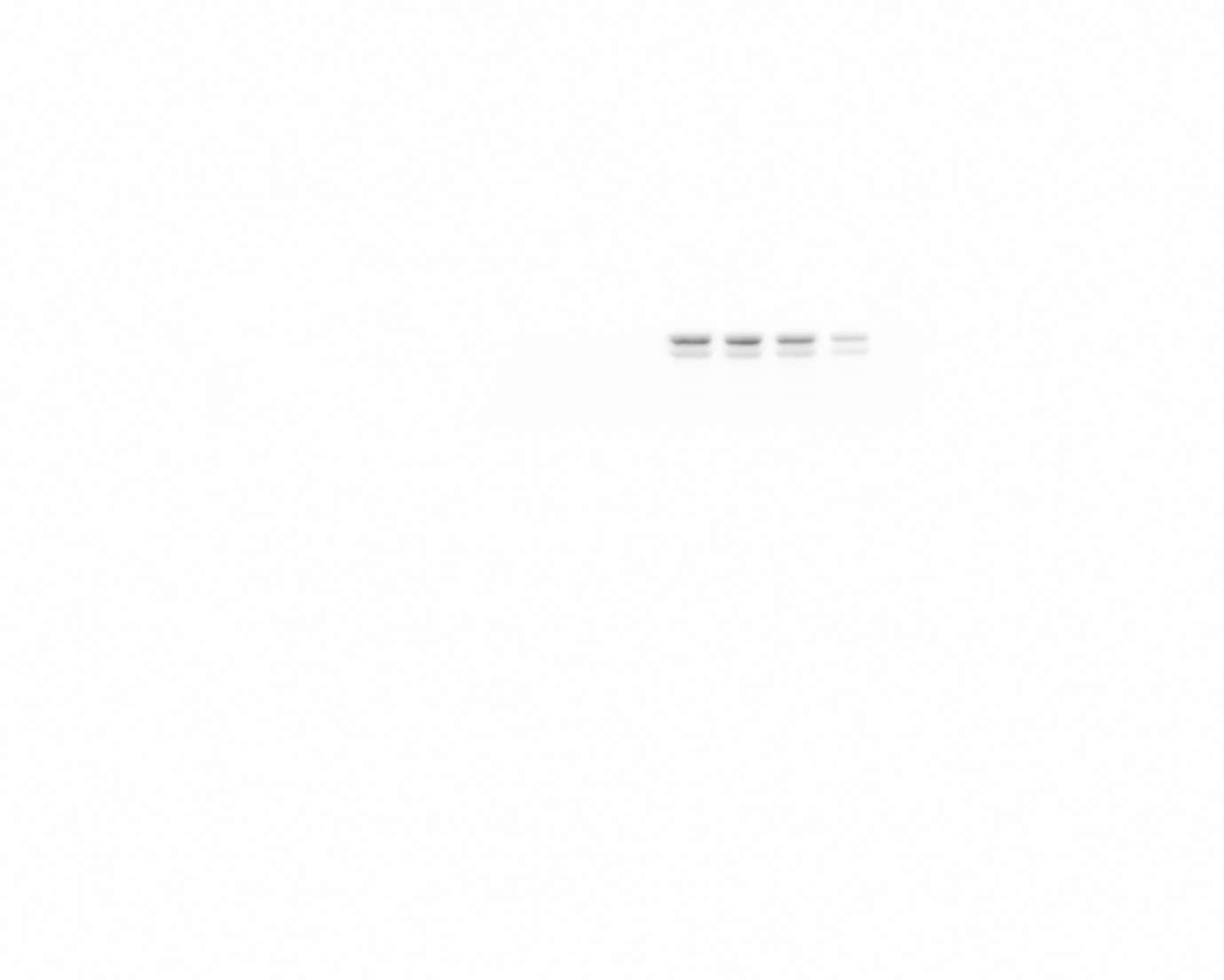


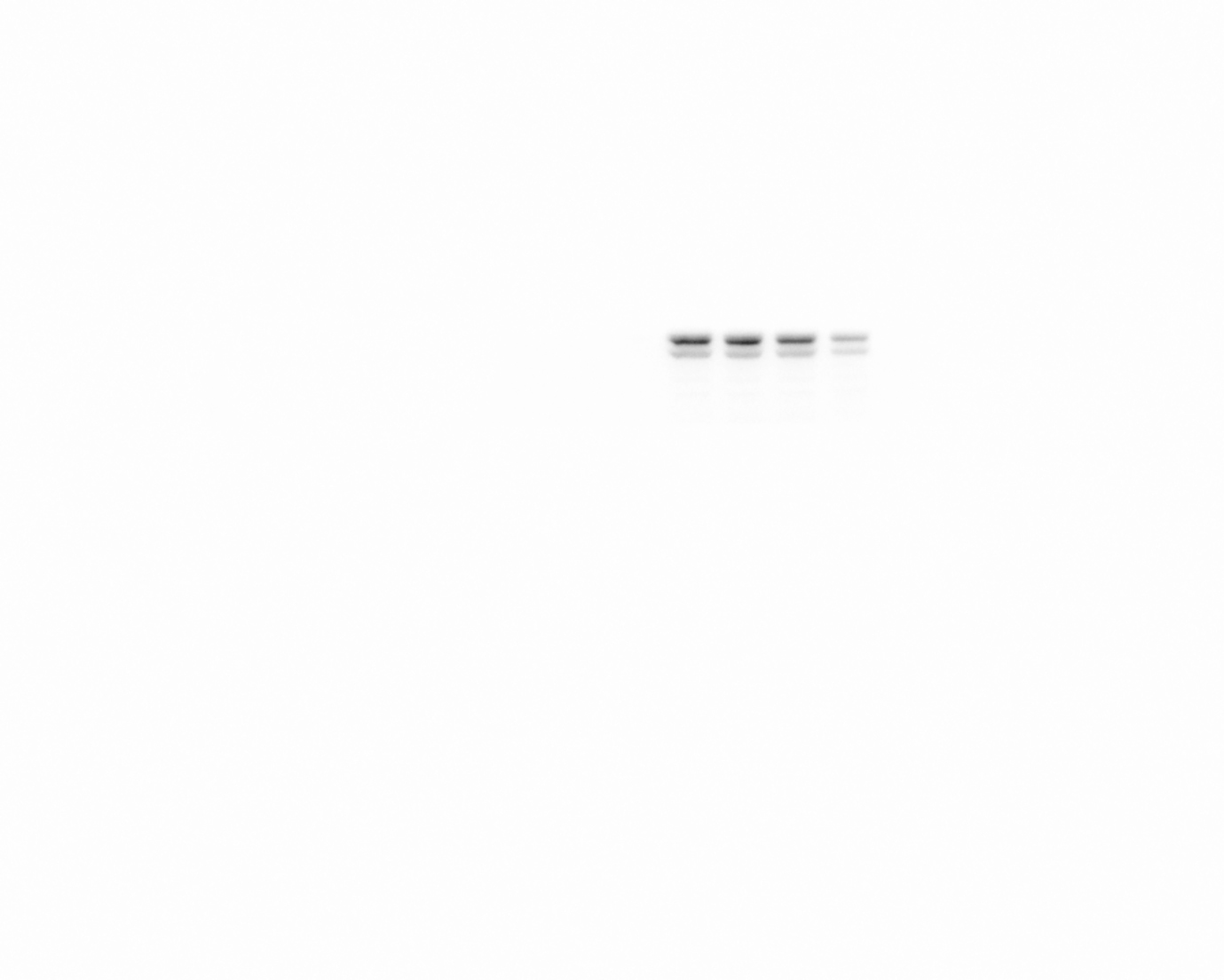


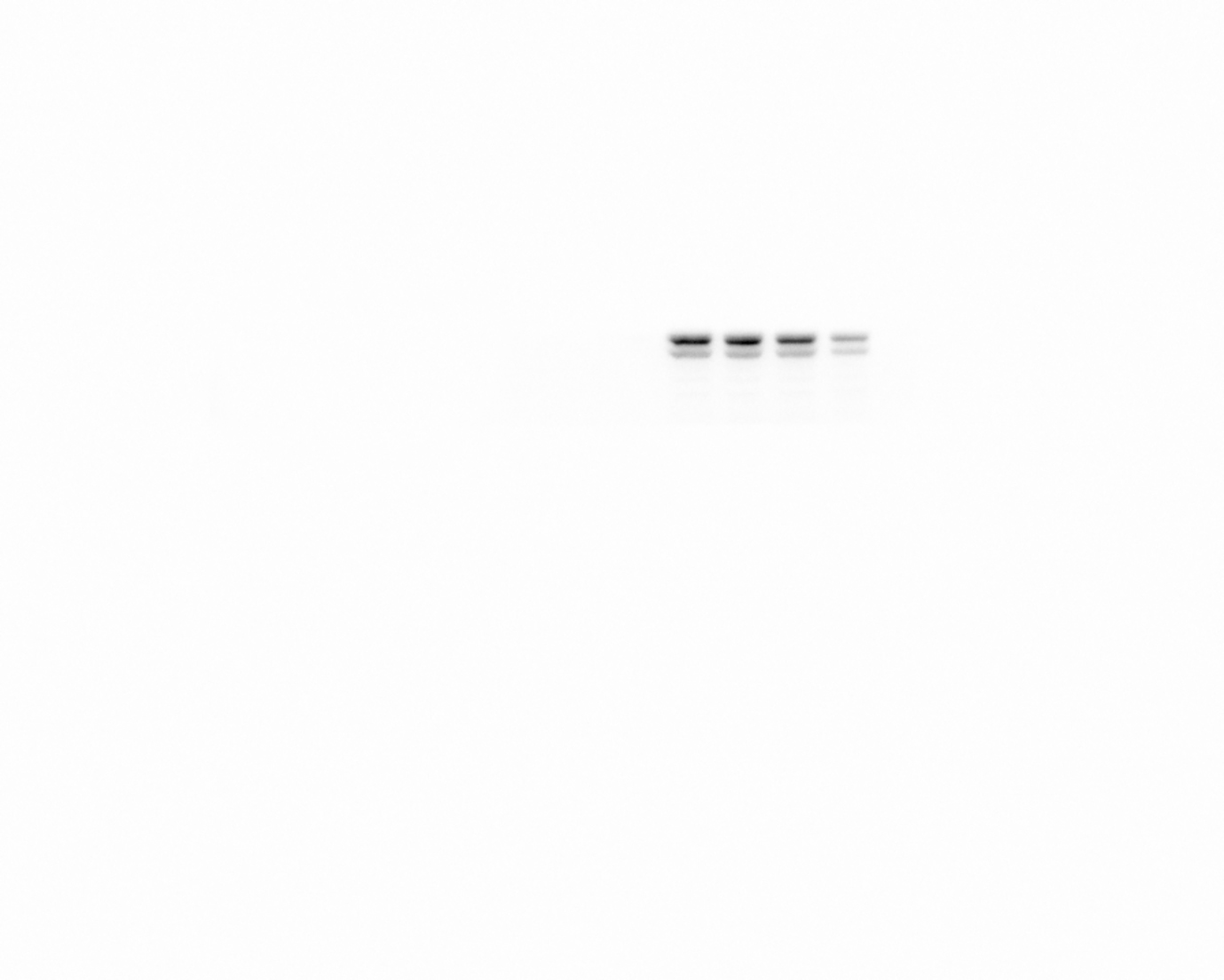


Fig3A.Western blot raw image


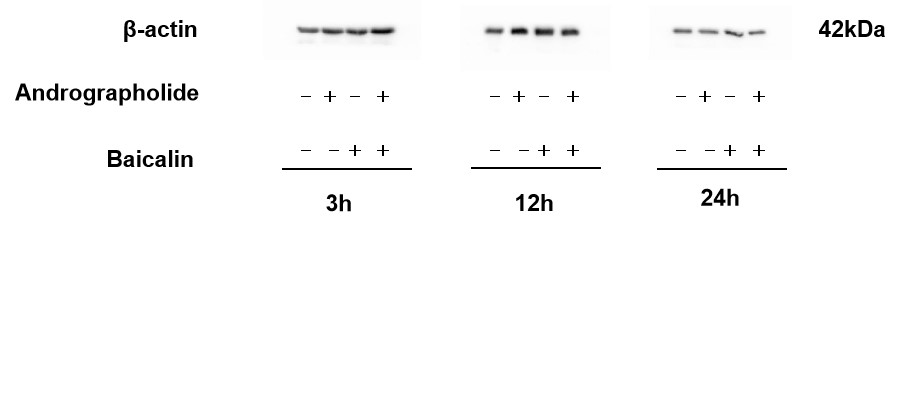


Multiple exposed images:

β-actin：


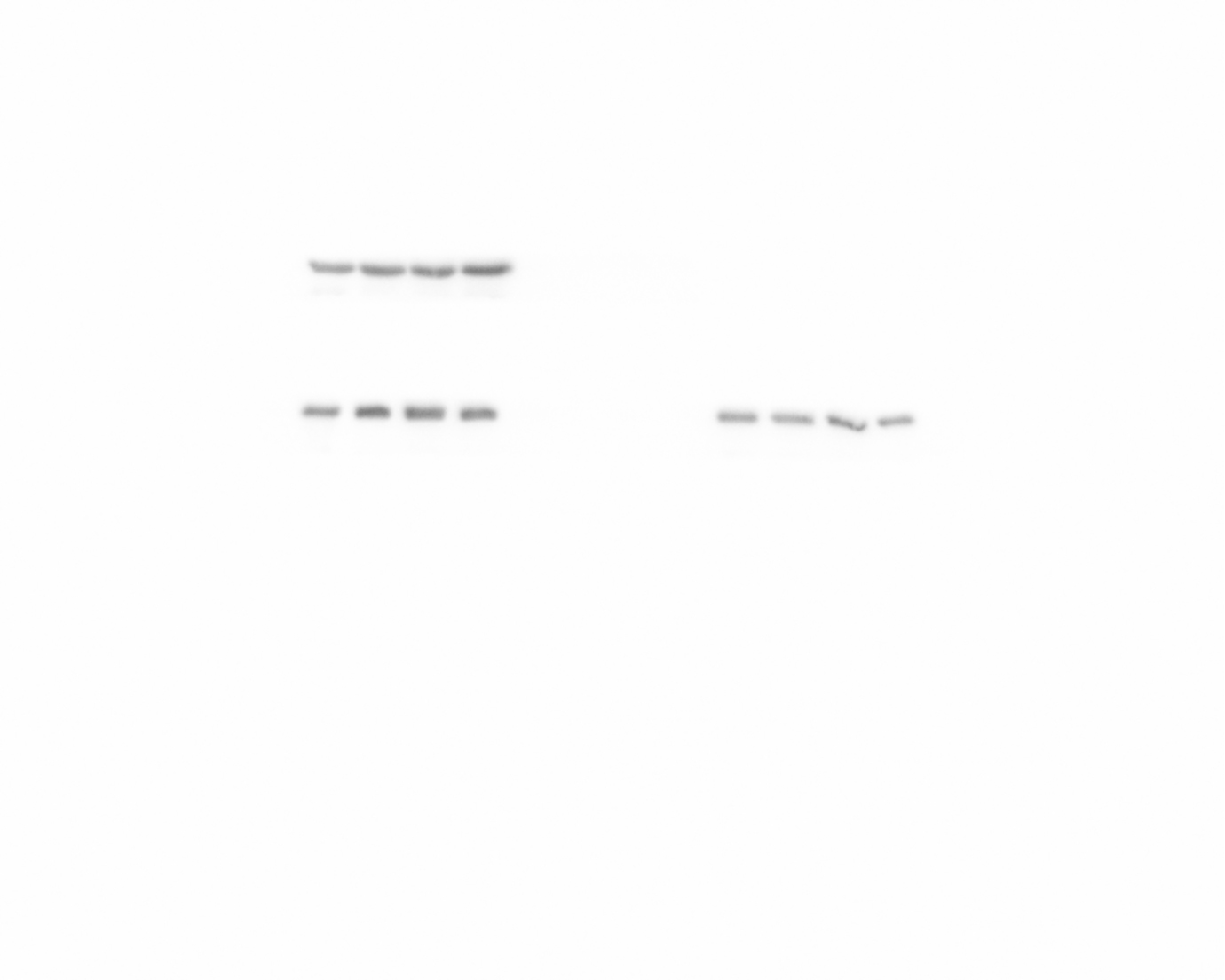


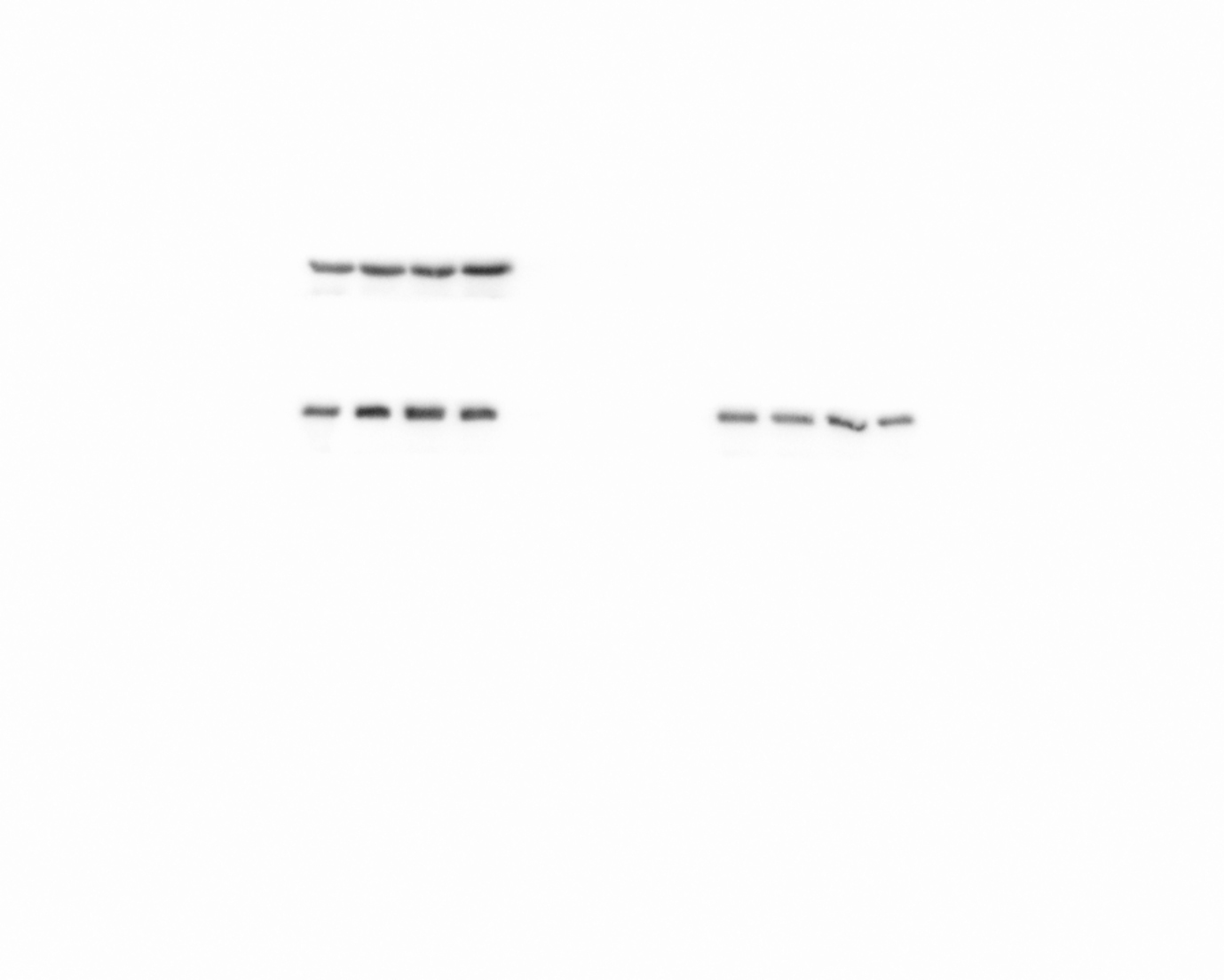


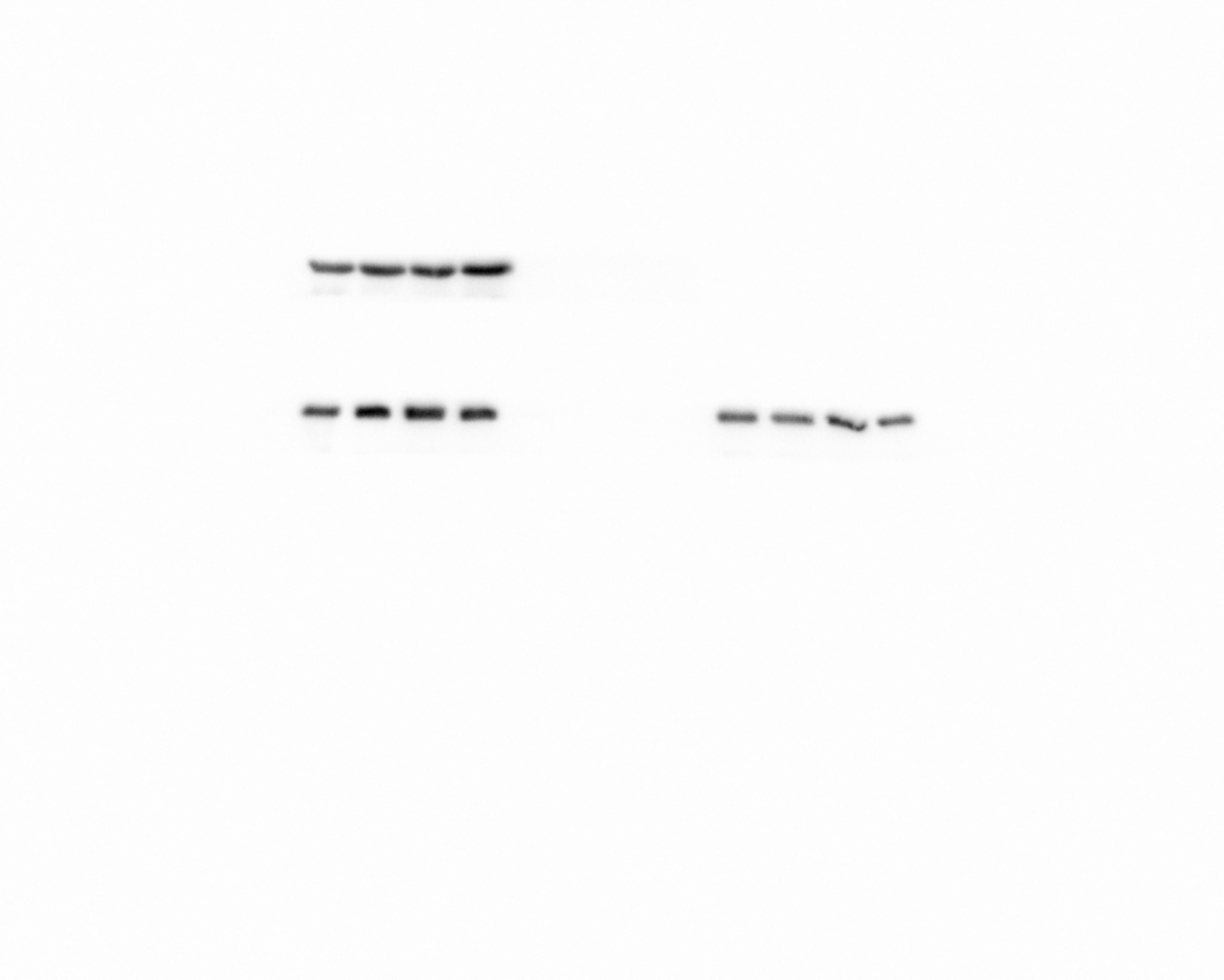


Fig3A.Western blot raw image


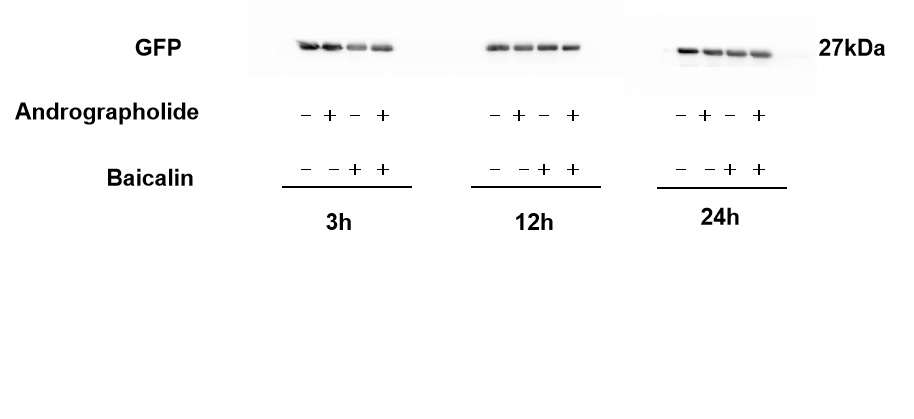


Multiple exposed images:

①GFP：


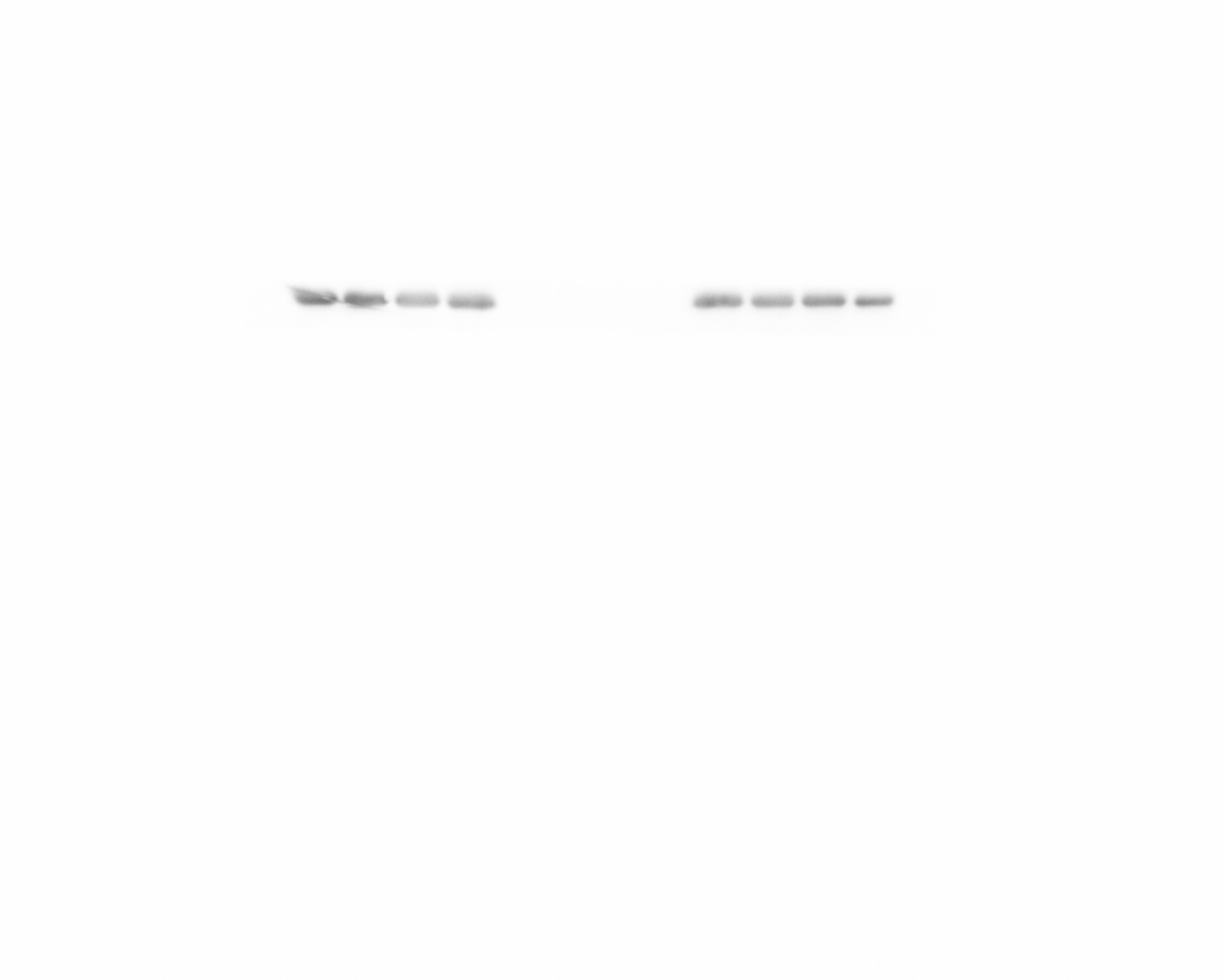


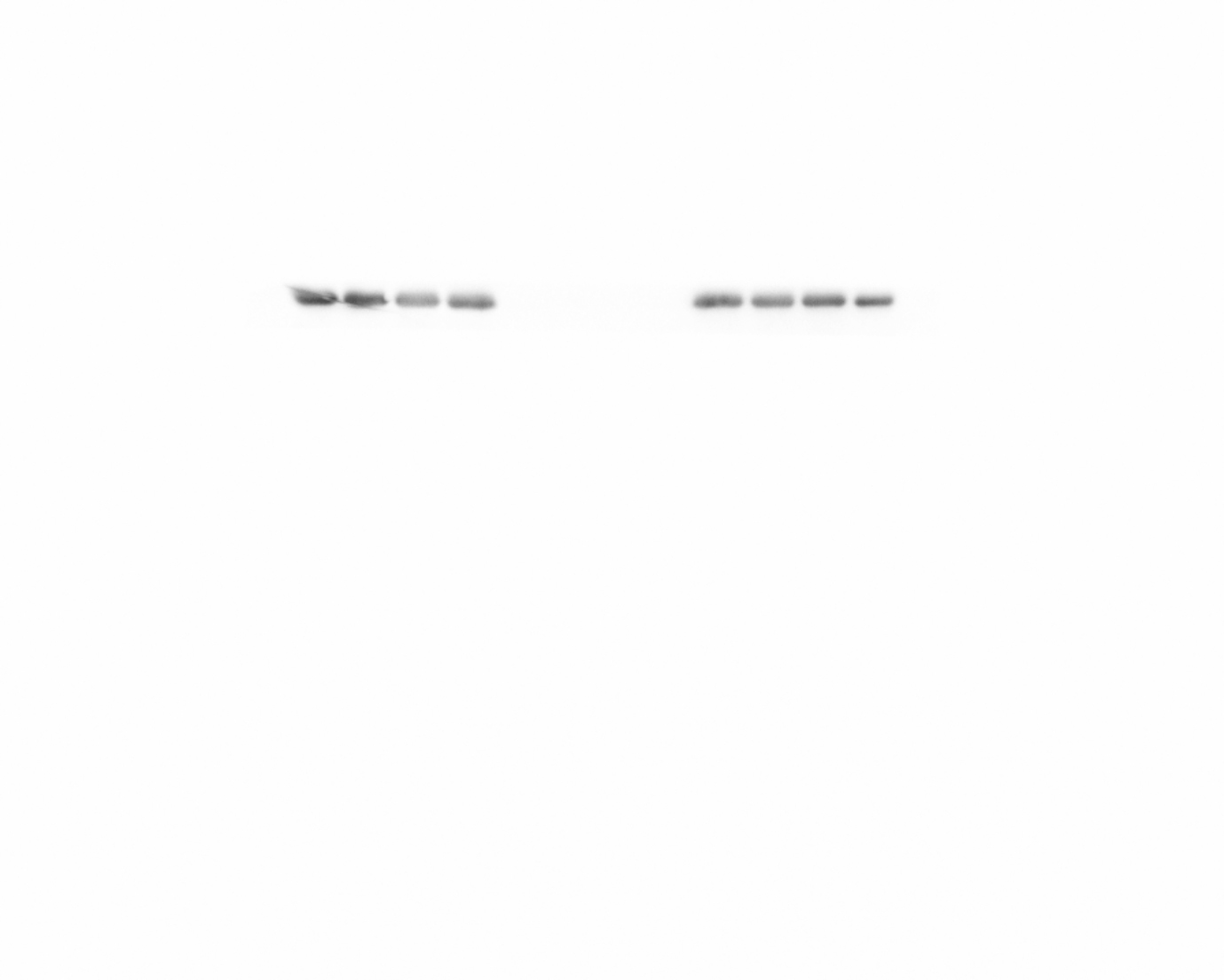


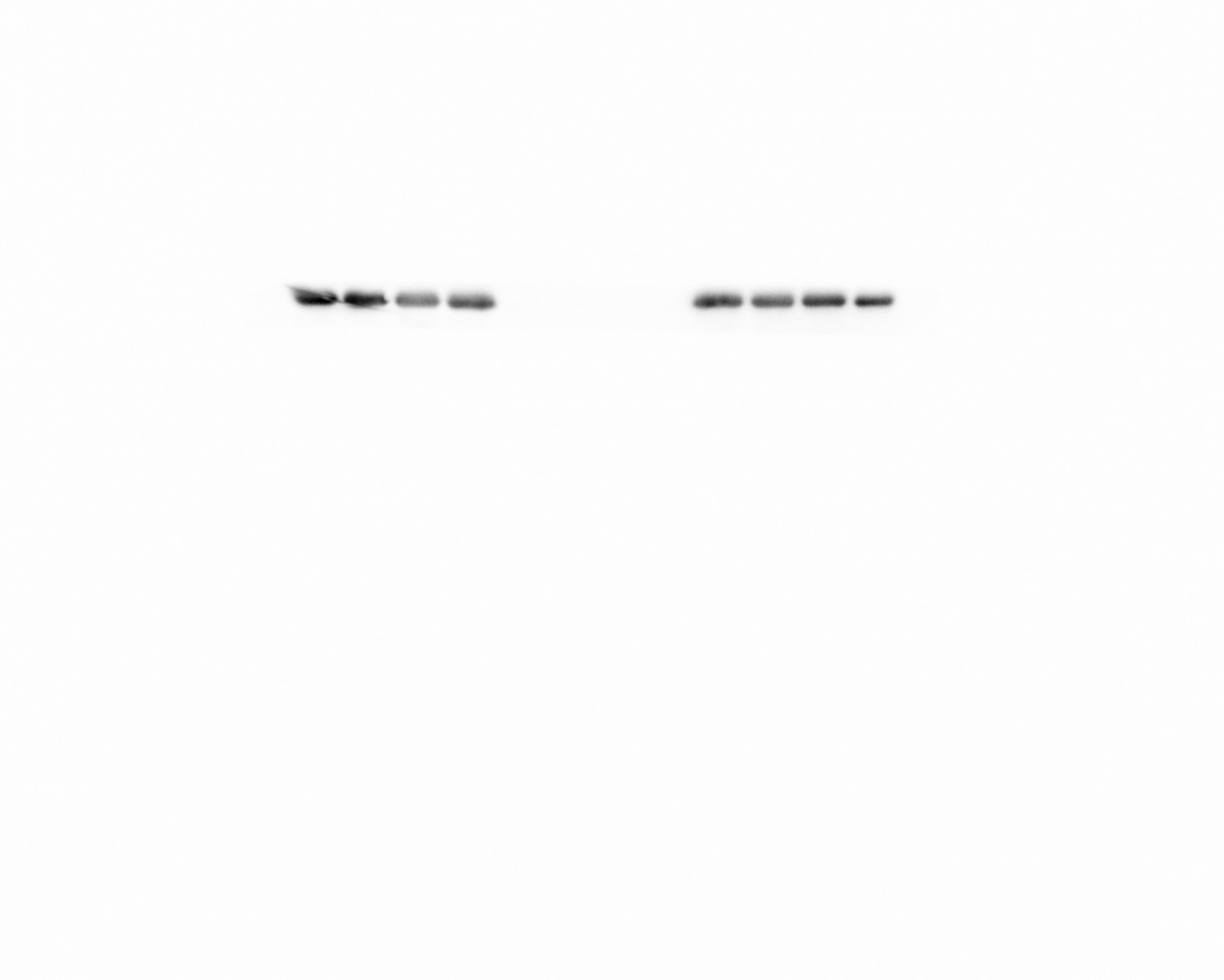


②GFP：


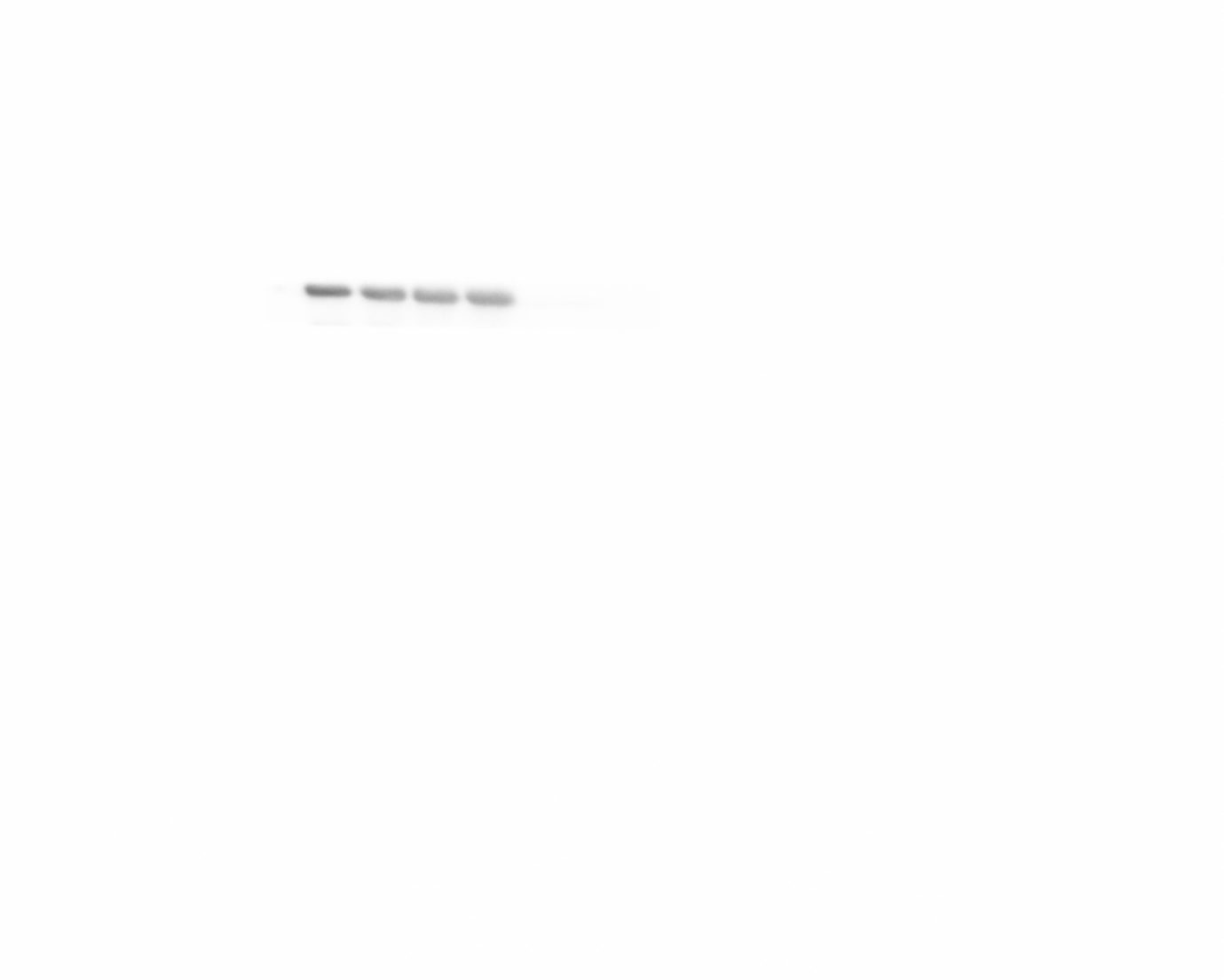


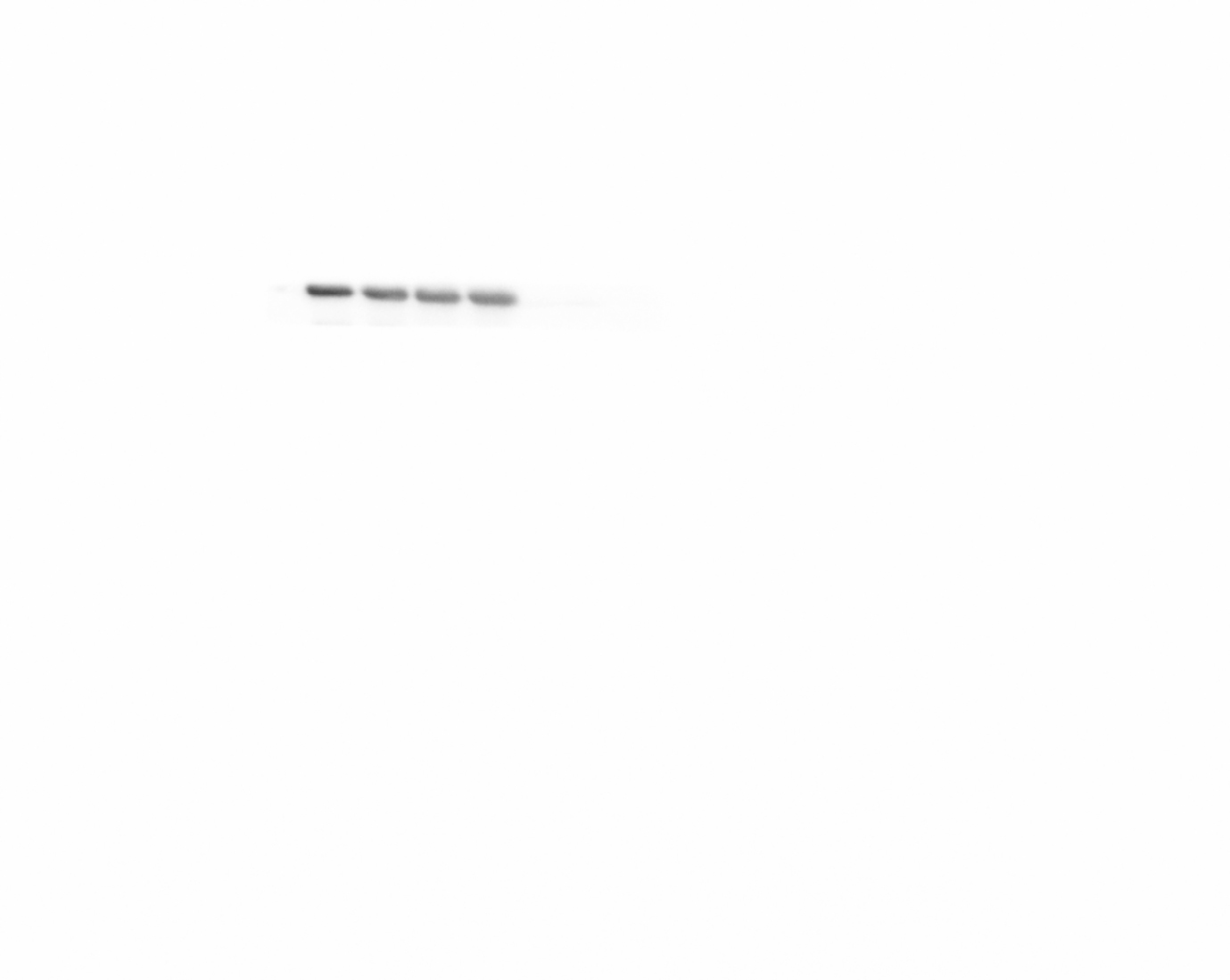


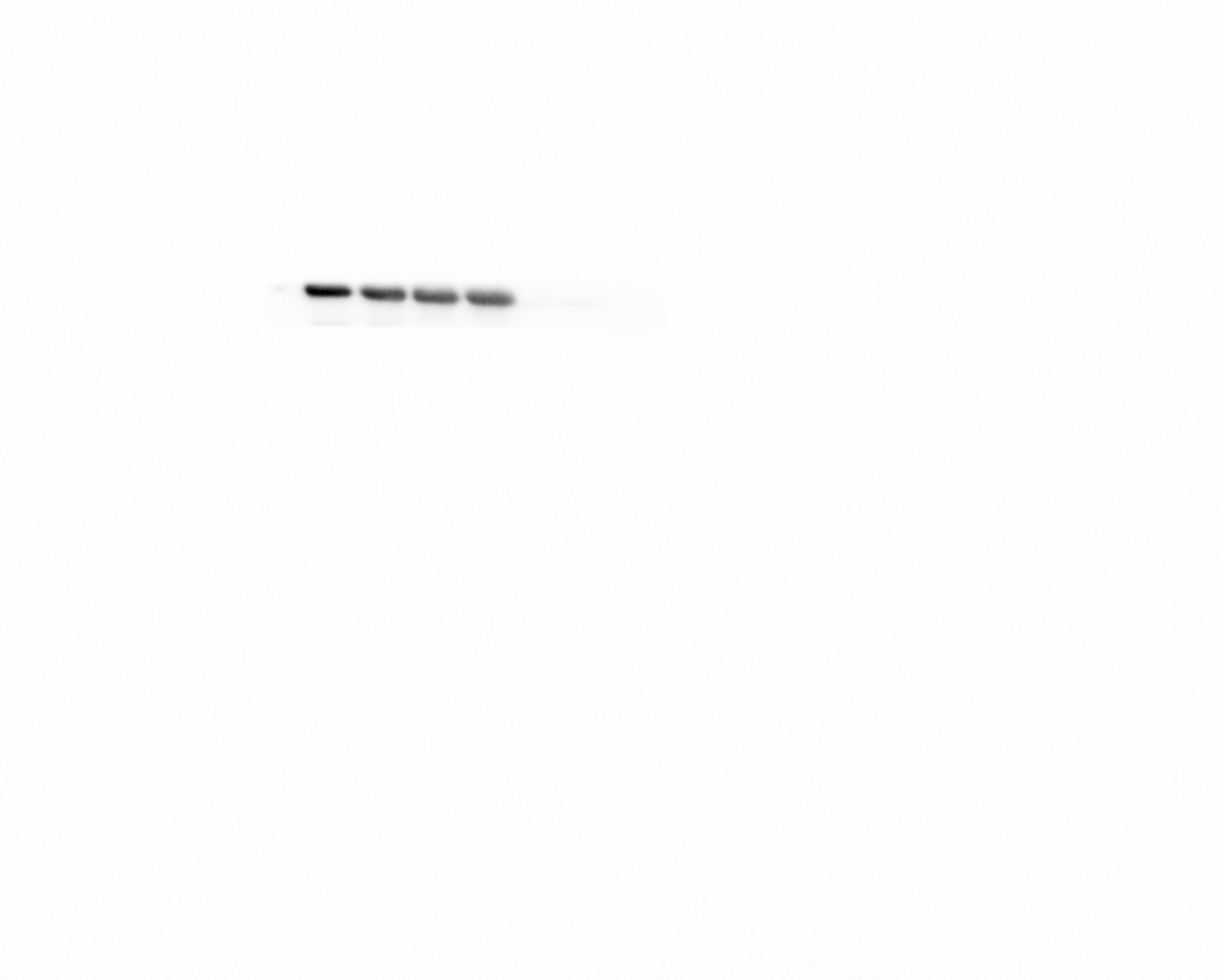


Fig3B.Western blot raw image


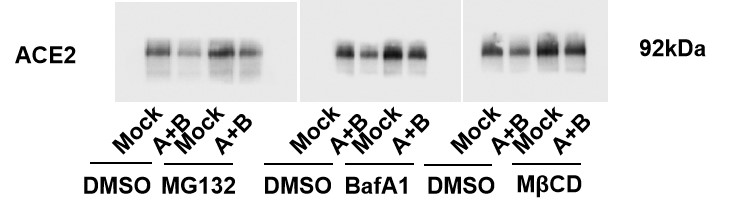


Multiple exposed images:

ACE2：


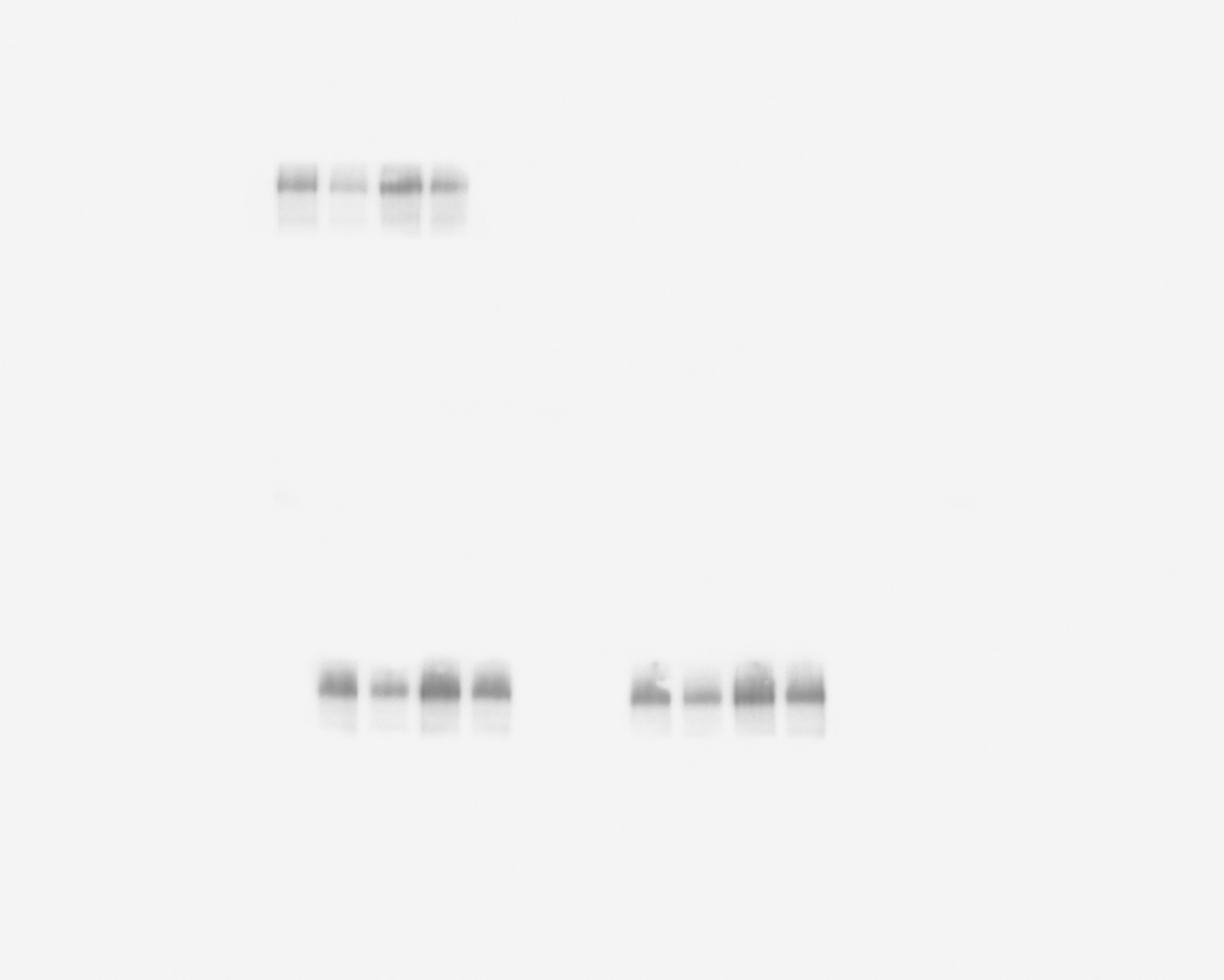


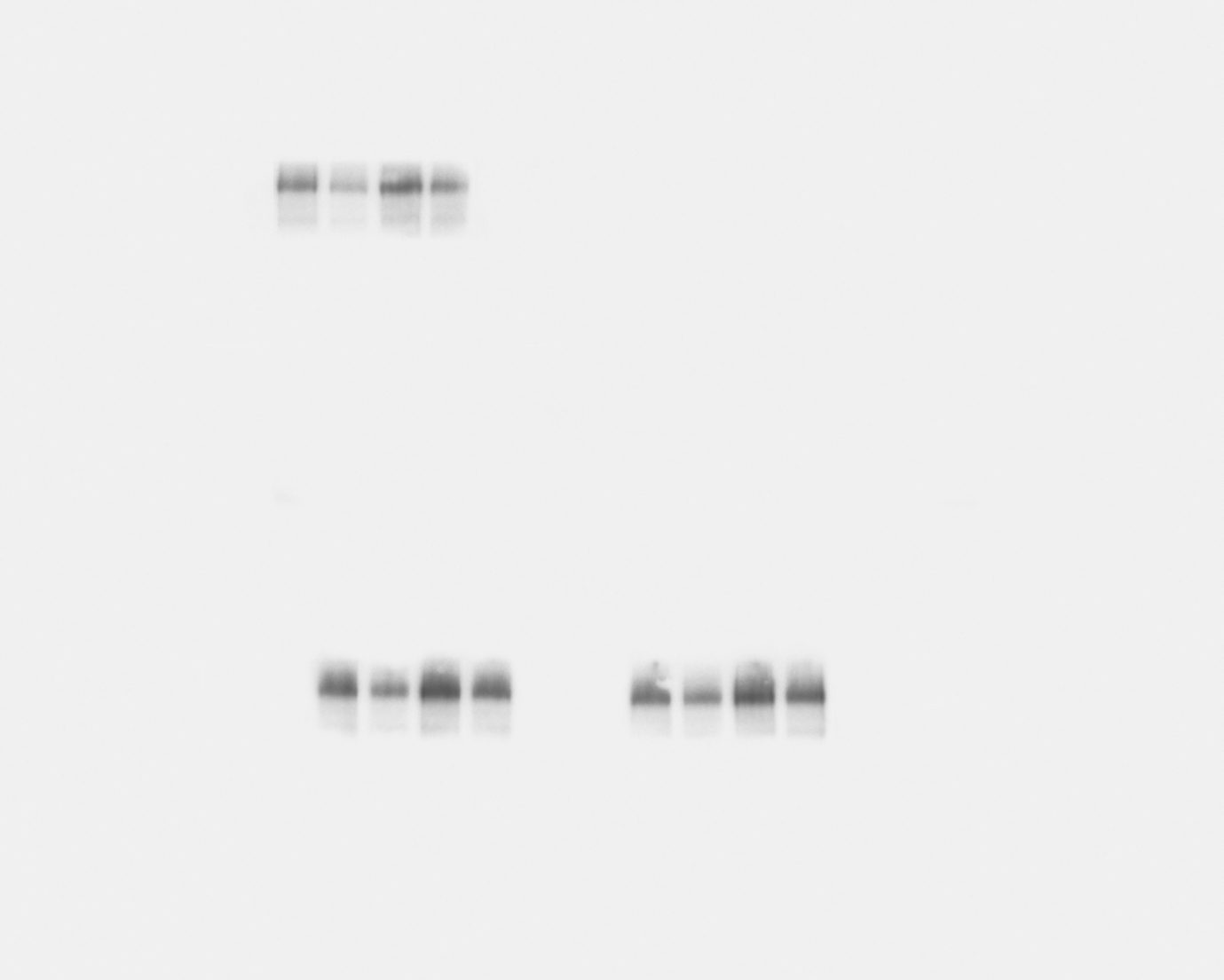


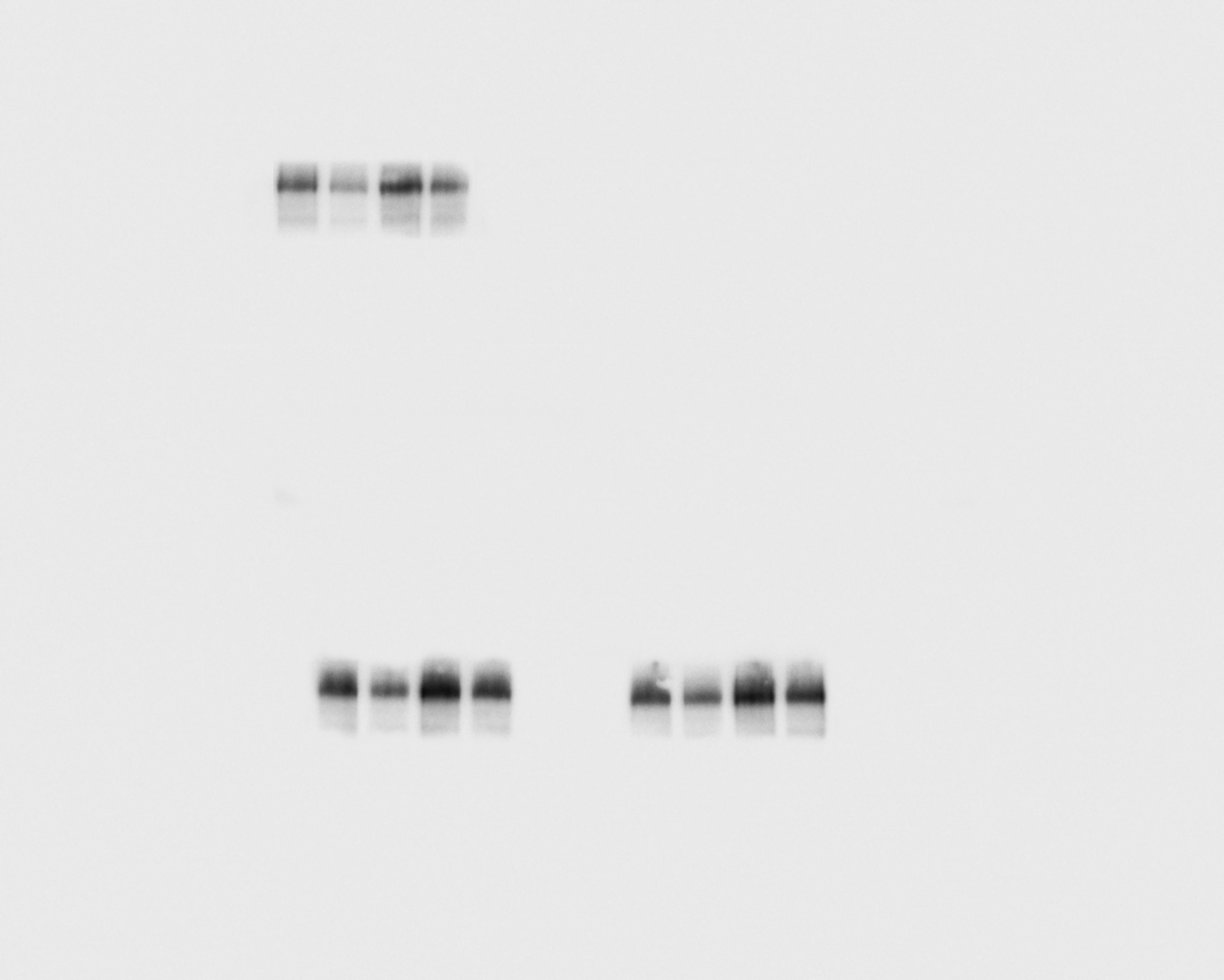


Fig3B.Western blot raw image


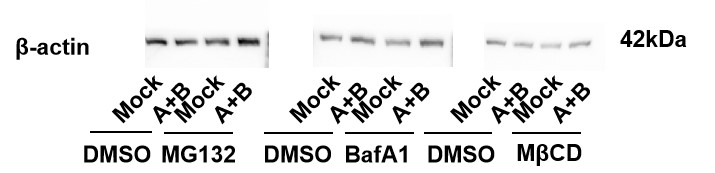


Multiple exposed images:

β-actin：


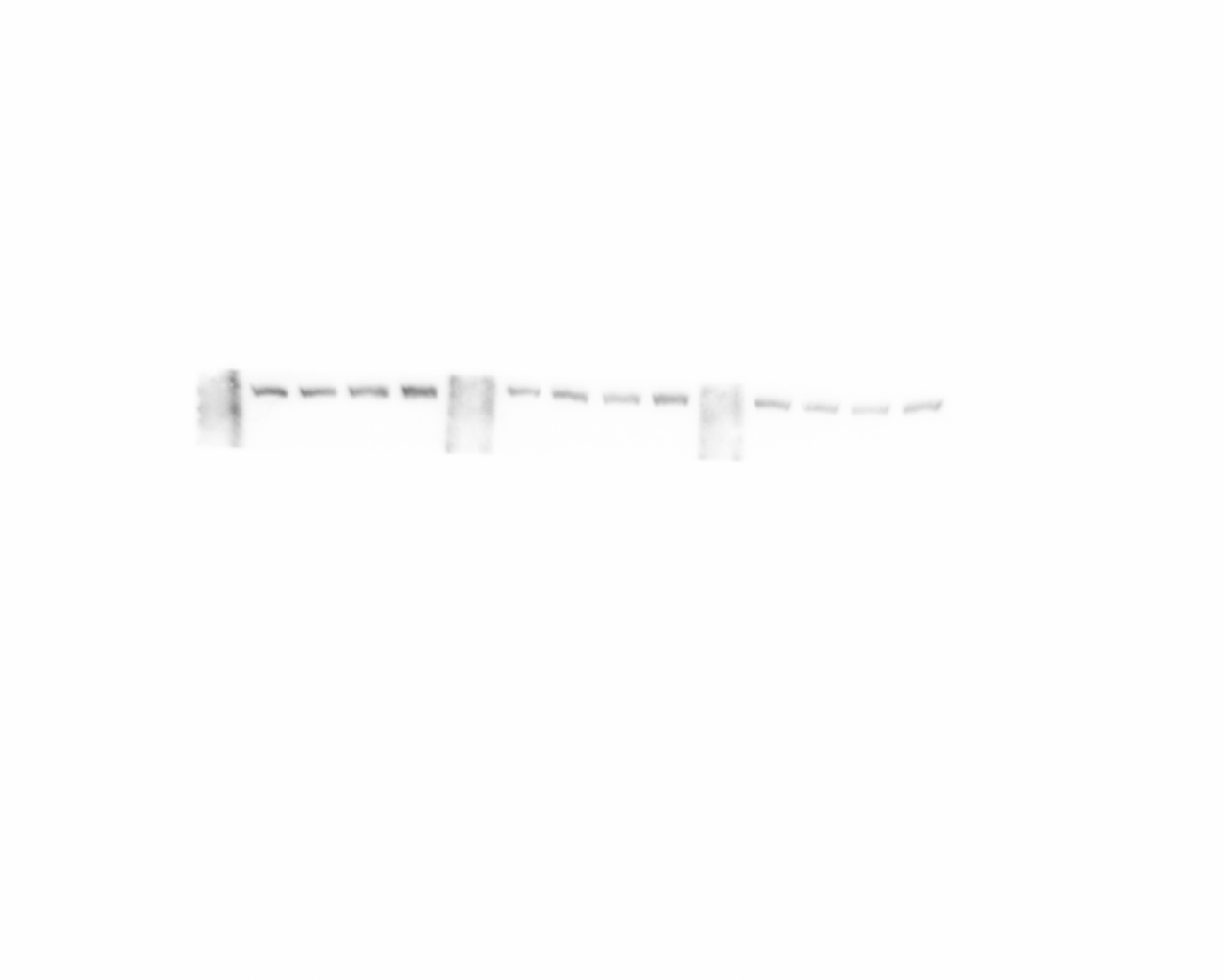


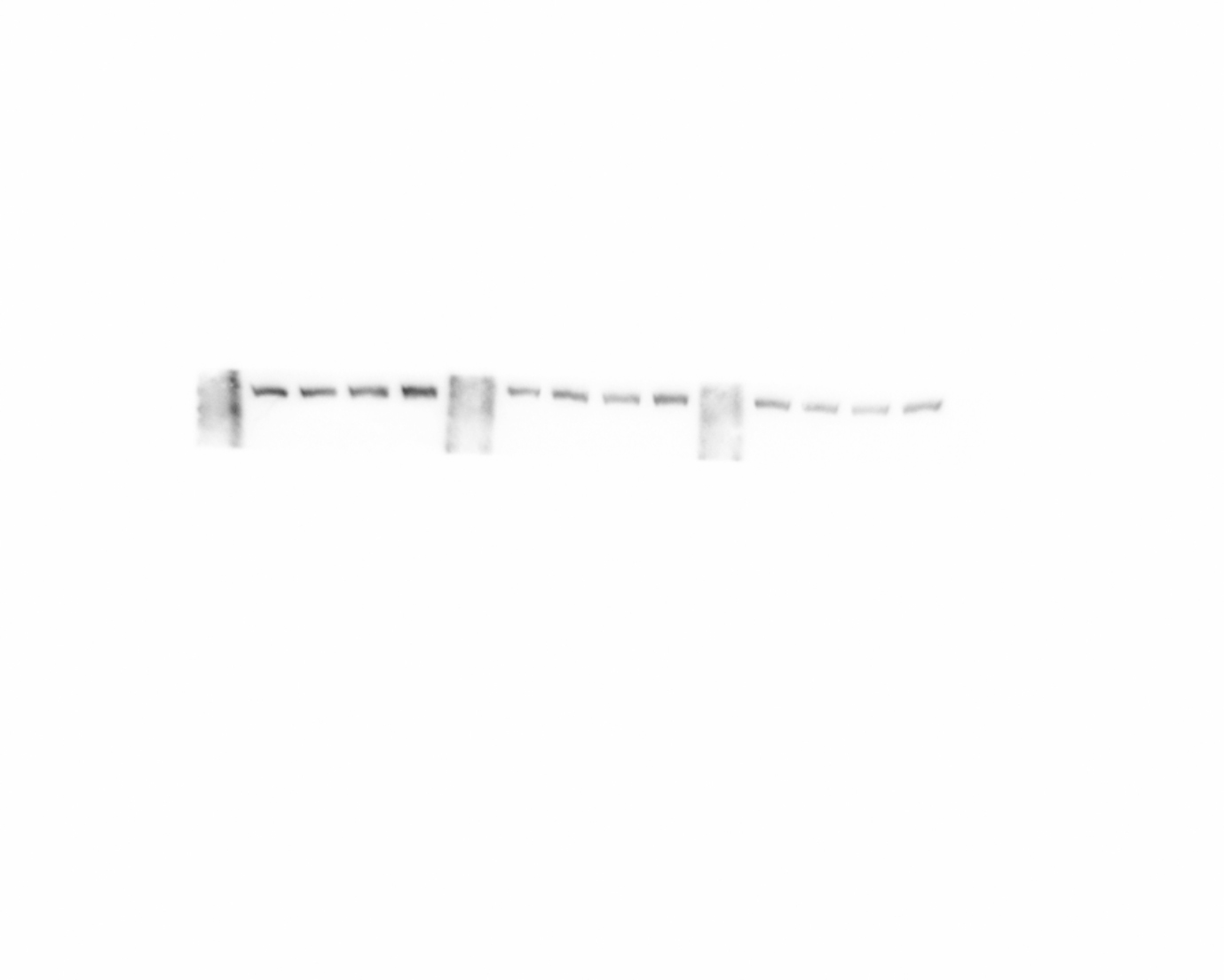


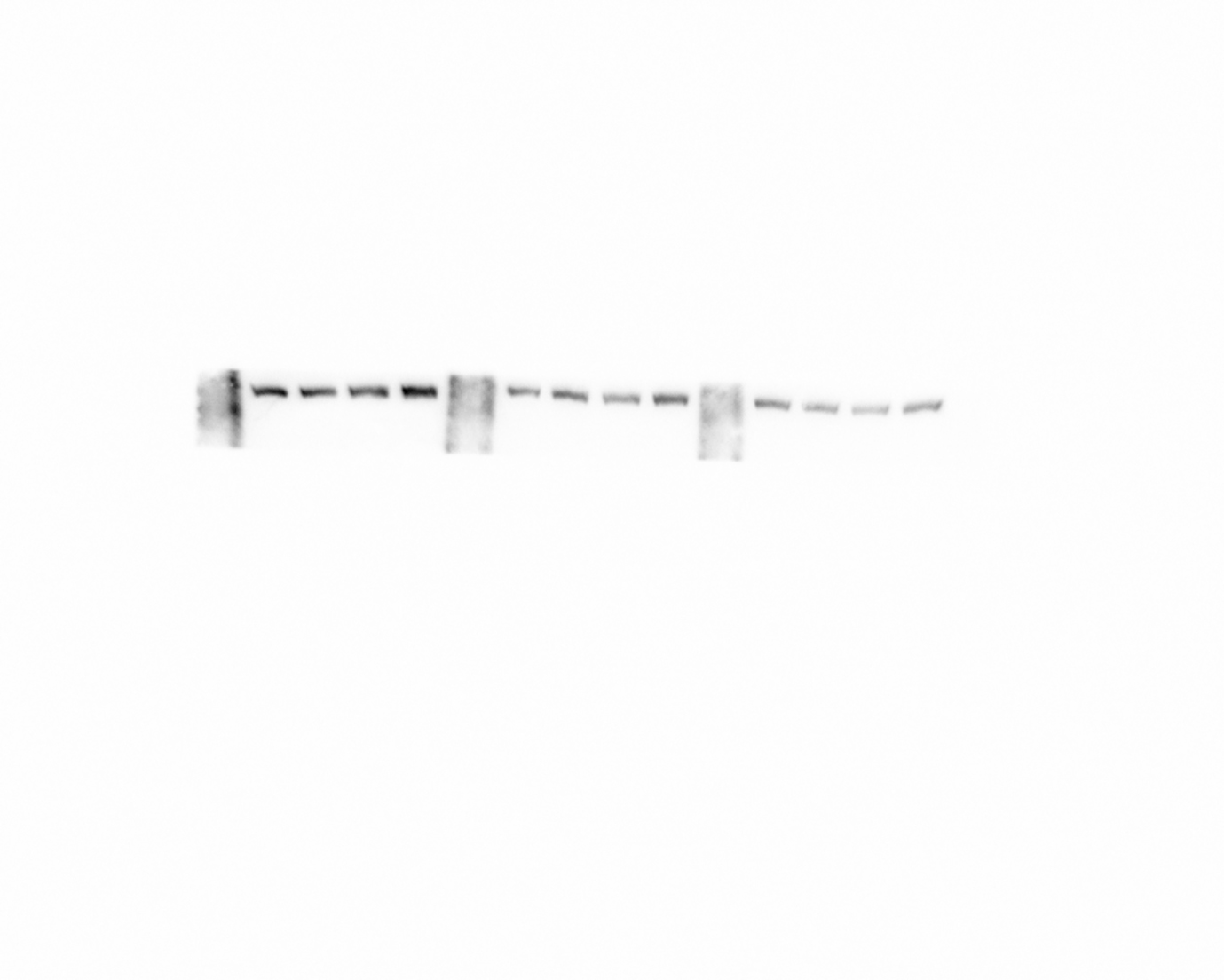


Fig3B.Western blot raw image


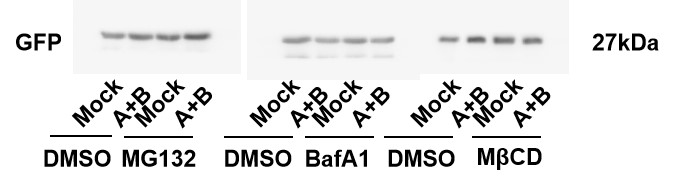


Multiple exposed images:

GFP：


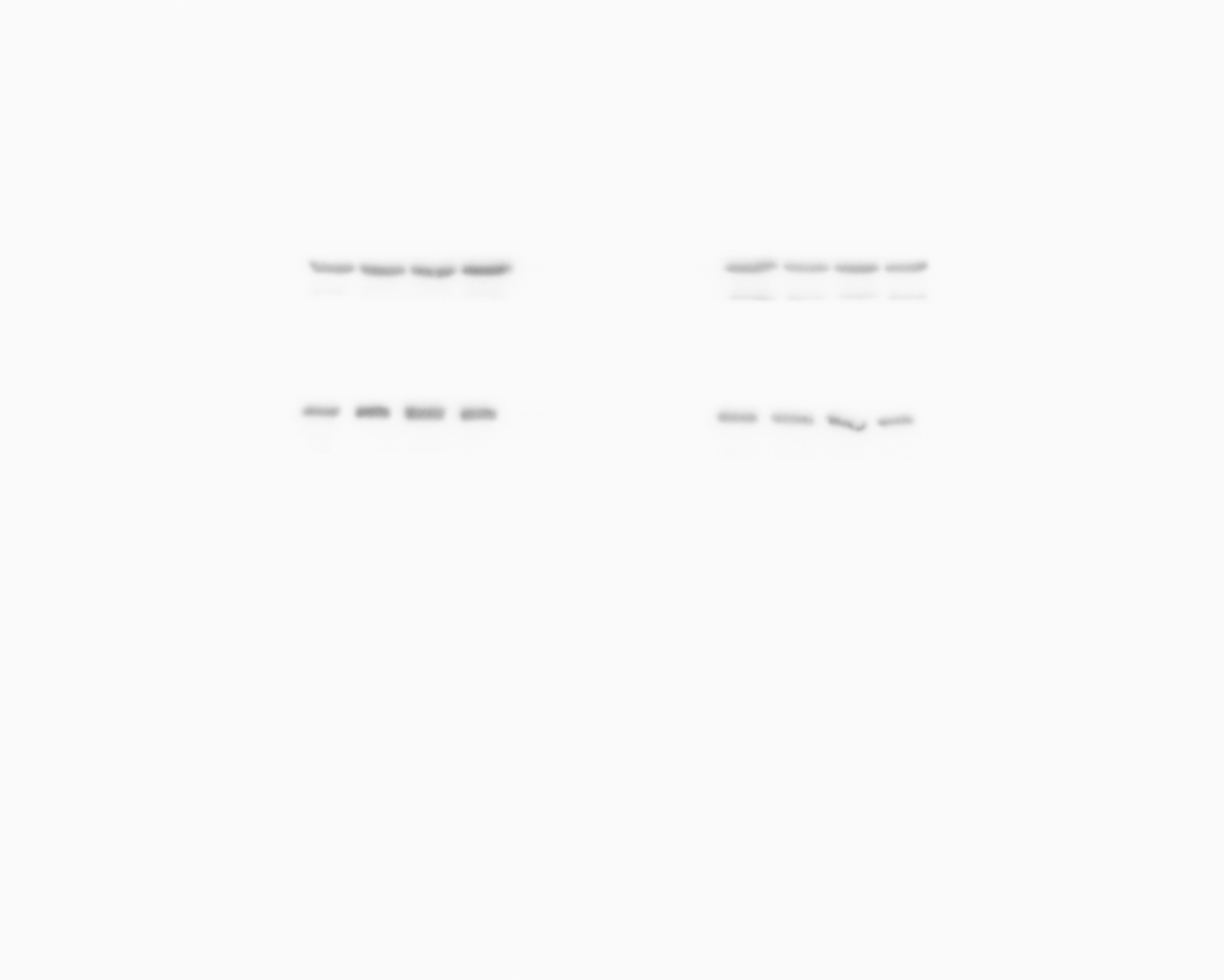


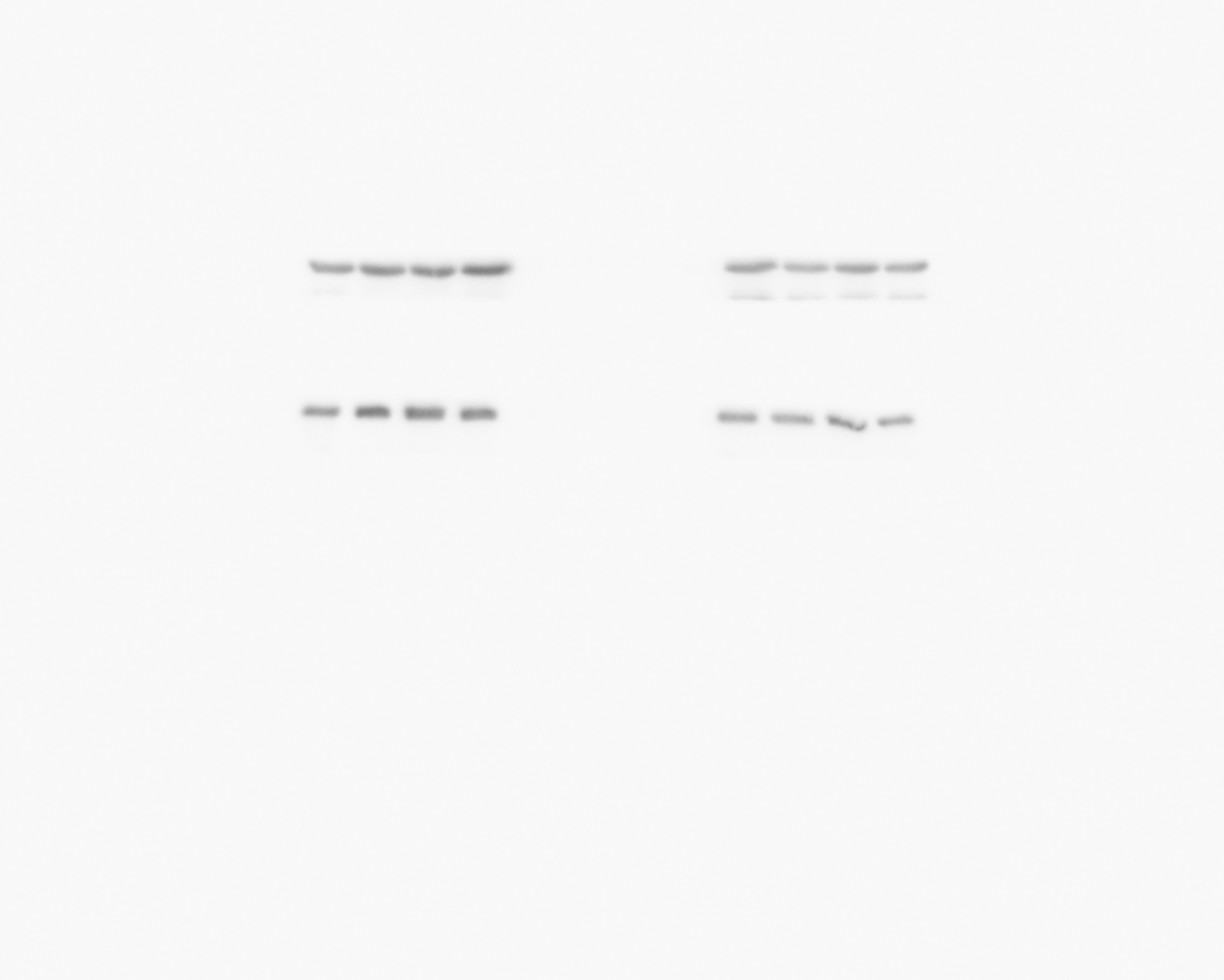


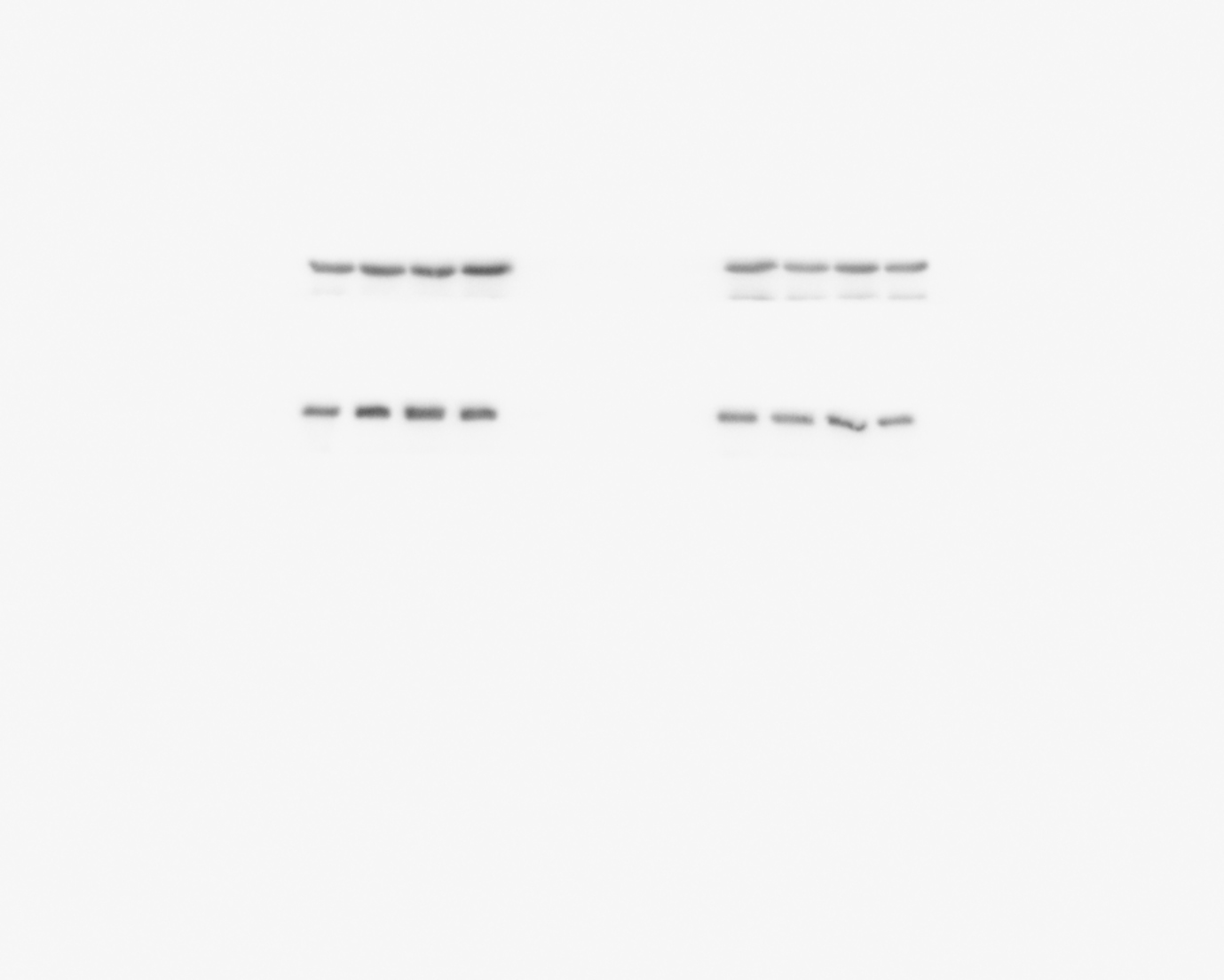


exposure equipment:


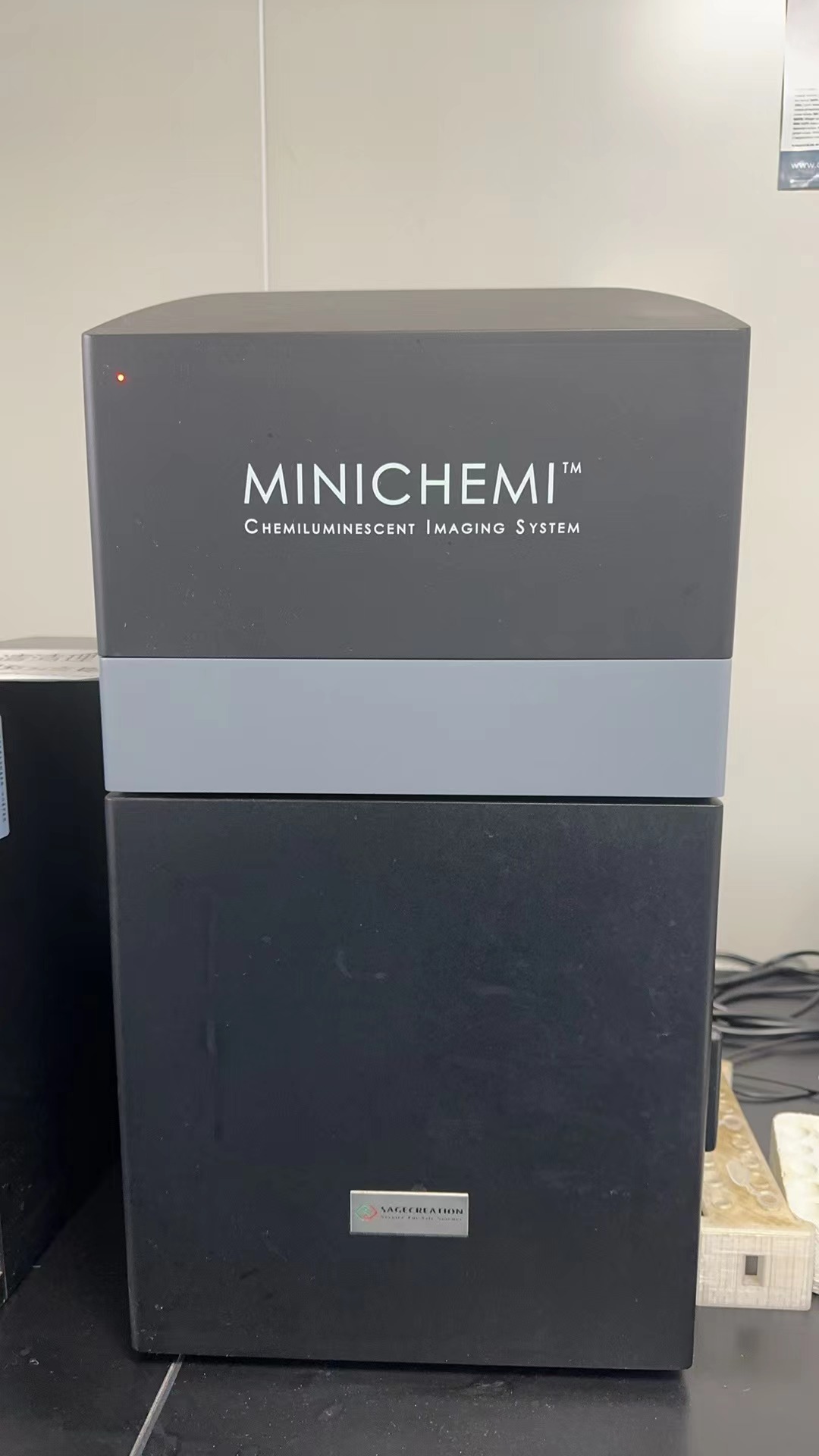

Supplement: Supplementary file 3 — Supplementary Information 3. [file 41598_2024_54722_MOESM3_ESM.doc]
